# Influence of energy deficiency on the subcellular processes of *Substantia Nigra Pars Compacta* cell for understanding Parkinsonian neurodegeneration

**DOI:** 10.1038/s41598-021-81185-9

**Published:** 2021-01-18

**Authors:** Vignayanandam Ravindernath Muddapu, V. Srinivasa Chakravarthy

**Affiliations:** Computational Neuroscience Lab, Department of Biotechnology, Bhupat and Jyoti Mehta School of Biosciences, Indian Institute of Technology Madras, Sardar Patel Road, Chennai, 600036 Tamil Nadu India

**Keywords:** Biochemical reaction networks, Cellular signalling networks, Computational models, Biophysical models

## Abstract

Parkinson’s disease (PD) is the second most prominent neurodegenerative disease around the world. Although it is known that PD is caused by the loss of dopaminergic cells in substantia nigra pars compacta (SNc), the decisive cause of this inexorable cell loss is not clearly elucidated. We hypothesize that “Energy deficiency at a sub-cellular/cellular/systems level can be a common underlying cause for SNc cell loss in PD.” Here, we propose a comprehensive computational model of SNc cell, which helps us to understand the pathophysiology of neurodegeneration at the subcellular level in PD. The aim of the study is to see how deficits in the supply of energy substrates (glucose and oxygen) lead to a deficit in adenosine triphosphate (ATP). The study also aims to show that deficits in ATP are the common factor underlying the molecular-level pathological changes, including alpha-synuclein aggregation, reactive oxygen species formation, calcium elevation, and dopamine dysfunction. The model suggests that hypoglycemia plays a more crucial role in leading to ATP deficits than hypoxia. We believe that the proposed model provides an integrated modeling framework to understand the neurodegenerative processes underlying PD.

## Introduction

More than 200 years after it was first described by Dr. James Parkinson as ‘shaking palsy’ we are still searching for a cure for Parkinson’s disease (PD), a neurodegenerative disorder characterized by the loss of dopaminergic cells in Substantia Nigra pars compacta (SNc)^1^. It is quite remarkable that the loss of cells in a small nucleus like SNc can have wide-ranging devastating effects in all the four major domains of brain function—sensory-motor, cognitive, affective, and autonomous^2^. While existing treatments manage the symptoms of PD—sometimes with miraculous effect—a genuine cure demands an understanding of the root cause of SNc cell loss. Recently, a new approach towards PD etiology—that metabolic deficiencies at subcellular/cellular/network level can be a major cause of SNc cell loss in PD—was gaining attention^3–6,7,57^.

In an earlier computational study, we have shown that metabolic deficiency at the systems/network level can lead to neurodegeneration of SNc neurons due to excitotoxicity caused by an overexcited Subthalamic Nucleus (STN)^3,7,8^. As a further step in understanding the PD pathophysiology, in the present study, we proposed to model the effects of metabolic deficiencies in SNc at the subcellular level. To this end, we need a comprehensive, holistic model of the SNc neuron with all the essential subcellular or molecular processes involved in PD pathogenesis. The model should include the standard molecular infrastructure like ion channels, active pumps, ion exchangers, dopamine (DA) turnover processes, energy metabolism pathways, and calcium buffering mechanisms and be able to simulate a rich vein of PD-related molecular processes such as alpha-synuclein aggregation, Lewy body formation, reactive oxygen species (ROS) production, levodopa (L-DOPA) uptake, and apoptotic pathways. Several researchers had tried to model parts of the extensive chemical network involved in subcellular PD pathogenesis^9–13^. From their modelling efforts, it was evident that developing such a comprehensive model of SNc neuron would be a significant leap in understanding the subcellular mechanisms underlying neurodegeneration in PD. A comprehensive literature survey on modelling efforts related to PD pathogenesis was recently published^14,15^.

The proposed computational study aims to see how deficits in the supply of energy substrates (glucose and oxygen) lead to a deficit in adenosine triphosphate (ATP), and furthermore, deficits in ATP are the common factor underlying the pathological changes in alpha-synuclein, ROS, calcium, and DA. Here, we propose a comprehensive computational model of SNc cell, which helps us in understanding the pathophysiology of neurodegeneration in PD. The model is expected to help resolve several outstanding questions of PD pathology, e.g., the recurrent confusion of cause and effect—is alpha-synuclein aggregation a cause or an effect of PD? If the hypothesis that the model set out to investigate ultimately proves to be true, it will be demonstrated that energy deficiency underlies all the molecular level manifestations of PD. Such a demonstration, naturally, requires extensive and directed experimentation, and the present model could perhaps serve as a blueprint for rolling out such an experimental program.

The model is developed as per the following stages. Firstly, each of the cellular processes in the model was calibrated by experimental data. Secondly, model responses to electrical and chemical stimulations were carried out to observe their effects on different vital molecular players in the SNc neuron. Finally, hypoglycemic and hypoxic conditions were simulated in the model to understand their adaptability to the energy crisis and to identify the different regimes, normal and pathological, in which the model operates.

## Methods

**Figure 1:**
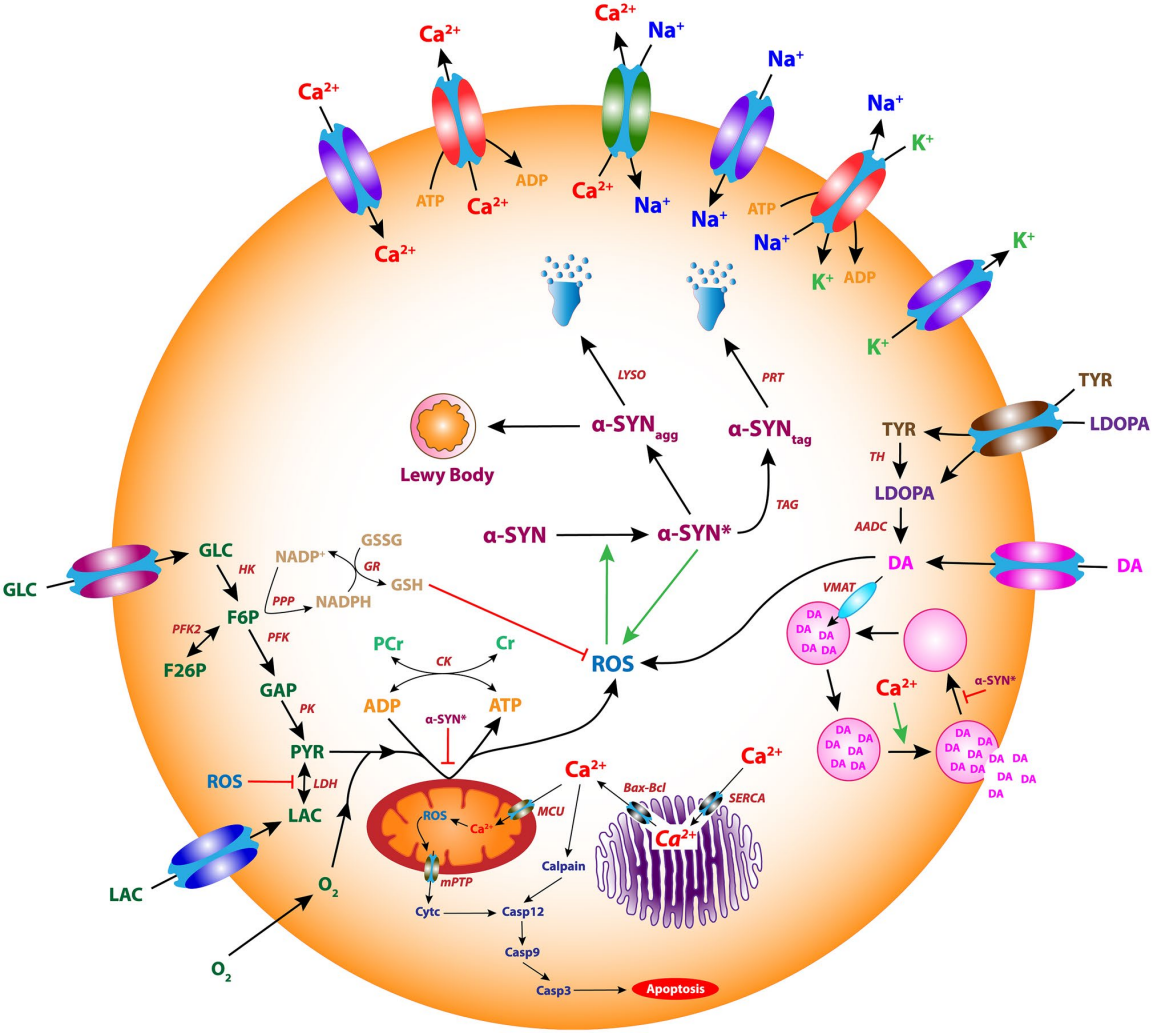
The proposed comprehensive model of the SNc neuron.

The proposed comprehensive single-cell model of SNc dopaminergic neurons consists of ion-channel dynamics^13^, calcium buffering mechanisms^13,16^, energy metabolism pathways^10,17^, DA turnover processes^9^, L-DOPA-uptake mechanisms^12^, apoptotic pathways^18^ and molecular pathways involved in PD pathology^10^ (Fig. 1).

### Ion channels

Modelling the behavior of a single neuron often requires detailed dynamics for a particular neuron type, since distinct electrophysiological and morphological features characterize each type of neuron. Dopaminergic neurons in substantia nigra exhibit two distinct firing patterns: low-frequency irregular tonic or background firing ($1 - 5 \,{\text{Hz}}$) and high-frequency regular phasic or burst firing^19^ ($\sim 20\,{\text{ Hz}}$). Dopaminergic neurons are autonomously active and produce a constant background firing pattern on which bursts may be superimposed.

We have adapted the single-compartmental biophysical model of SNc^13^, where ion-channel dynamics is dependent on ATP levels. Other previously published dopaminergic neuronal models are specified in Supplementary Table 1. The ionic currents which were considered in the soma (Supplementary Fig. 1) are voltage-dependent sodium currents ($I_{Na}$), voltage-dependent potassium currents $\left( {I_{K} } \right)$, voltage-dependent L-type calcium current $\left( {I_{CaL} } \right)$, calcium-dependent potassium current $\left( {I_{{K\left( {Ca} \right)}} } \right)$, leak current $\left( {I_{L} } \right)$, sodium–potassium ATPase $\left( {I_{NaK} } \right)$, calcium ATPase $\left( {I_{pmca} } \right)$ and sodium-calcium exchanger $\left( {I_{NaCaX} } \right)$.

The membrane potential equation for the SNc soma $\left( V \right)$ is given by,

1 $$\frac{d\left( V \right)}{{dt}} = \frac{{F \times vol_{cyt} }}{{C_{snc} \times AR_{pmu} }} \times \left[ { J_{m,Na} + 2 \times J_{m,Ca} + J_{m,K} + J_{inp} } \right],$$

where, $F$ is the Faraday’s constant, $C_{snc}$ is the SNc membrane capacitance, $vol_{cyt}$ is the cytosolic volume, $AR_{pmu}$ is the cytosolic area, $J_{m,Na}$ is the sodium membrane ion flux, $J_{m,Ca}$ is the calcium membrane ion flux, $J_{m,K}$ is the potassium membrane ion flux, and $J_{inp}$ is the overall input current flux.

### Plasma membrane ion channels

The intracellular calcium concentration dynamics $\left( {\left[ {Ca_{i} } \right]} \right)$ is given by,

2 $$\frac{{d\left( {\left[ {Ca_{i} } \right]} \right)}}{dt} = J_{m,Ca} ,$$

3 $$J_{m,Ca} = - \frac{1}{{z_{Ca} \times F \times vol_{cyt} }} \times \left( {I_{CaL} + 2 \times I_{pmca} - 2 \times I_{NaCaX} } \right),$$

where, $z_{Ca}$ is the valence of calcium ion, $I_{CaL}$ is the L-type calcium channel current, $I_{pmca}$ is the ATP-dependent calcium pump current, $I_{NaCaX}$ is the sodium–potassium exchanger current, $F$ is the Faraday’s constant, and $vol_{cyt}$ is the cytosolic volume.

The voltage-dependent L-type calcium channel current $\left( {I_{CaL} } \right)$ is given by,

4 $$I_{CaL} \left( V \right) = \left( {\overline{g}_{Ca,L} \times O_{Ca,L} } \right) \times \left( {\sqrt {\left[ {Ca_{i} } \right] \times \left[ {Ca_{e} } \right]} } \right) \times \left( {\frac{{\sinh \left( {V_{D} - V_{Ca} } \right)}}{{\left( {\frac{{\sinh \left( {V_{D} } \right)}}{{V_{D} }}} \right)}}} \right),$$

5 $$O_{Ca,L} = m_{Ca,L} \times h_{Ca,L} ,$$

where, $\overline{g}_{Ca,L}$ is the maximal conductance for calcium channel, $O_{Ca,L}$ is the gating variable of calcium channel, $m_{Ca,L}$ is the activation gate of the L-type calcium channel, $h_{Ca,L}$ is the inactivation gate of L-type calcium channel, $\left[ {Ca_{i} } \right]$ is the intracellular calcium concentration, $\left[ {Ca_{e} } \right]$ is the extracellular calcium concentration, $V_{Ca}$ is the reversal potential for calcium ion, and $V_{D}$ is the voltage defined thermodynamic entity.

6 $$\frac{{d\left( {m_{Ca,L} } \right)}}{dt} = \frac{{\frac{1}{{\left( {1 + e^{{\left( { - \frac{{\left( {V + 15} \right)}}{7}} \right)}} } \right)}} - m_{Ca,L} }}{{7.68 \times e^{{\left( { - \left[ {\frac{V + 65}{{17.33}}} \right]^{2} } \right)}} + 0.723}},$$

7 $$h_{Ca,L} = \frac{0.00045}{{0.00045 + \left[ {Ca_{i} } \right]}}.$$

The intracellular sodium concentration $\left( {\left[ {Na_{i} } \right]} \right)$ dynamics is given by,

8 $$\frac{{d\left( {\left[ {Na_{i} } \right]} \right)}}{dt} = J_{m,Na} ,$$

9 $$J_{m,Na} = - \frac{1}{{z_{Na} \times F \times vol_{cyt} }} \times \left( {I_{NaT} + 3 \times I_{NaK} + 3 \times I_{NaCaX} } \right),$$

where, $z_{Na}$ is the valence of sodium ion, $I_{NaT}$ is the total sodium channel current, $I_{NaK}$ is the ATP-dependent sodium–potassium pump current, $I_{NaCaX}$ is the sodium–potassium exchanger current, $F$ is the Faraday’s constant, and $vol_{cyt}$ is the cytosolic volume.

The total sodium channel current is given by,

10 $$I_{NaT} = I_{Na} + I_{NaHCN} + I_{Nalk} ,$$

where, $I_{Na}$ is the voltage-dependent sodium channel current, $I_{NaHCN}$ is the hyperpolarization-activated cyclic nucleotide-gated sodium channel current, and $I_{Nalk}$ is the leaky sodium channel current.

The voltage-dependent sodium channel current $\left( {I_{Na} } \right)$ is given by,

11 $$I_{Na} \left( V \right) = \left( {\overline{g}_{Na} \times O_{Na} } \right) \times \left( {\sqrt {\left[ {Na_{i} } \right] \times \left[ {Na_{e} } \right]} } \right) \times \left( {\frac{{\sinh \left( {\frac{1}{2} \times \left( {V_{D} - V_{Na} } \right)} \right)}}{{\left( {\frac{{\sinh \left( {\frac{1}{2} \times V_{D} } \right)}}{{\left( {\frac{1}{2} \times V_{D} } \right)}}} \right)}}} \right),$$

12 $$O_{Na} = m_{Na}^{3} \times h_{Na} ,$$

where, $\overline{g}_{Na}$ is the maximal conductance for sodium channel, $O_{Na}$ is the gating variable of sodium channel, $m_{Na}$ is the activation gate of the sodium channel, $h_{Na}$ is the inactivation gate of the sodium channel, $\left[ {Na_{i} } \right]$ is the intracellular sodium concentration, $\left[ {Na_{e} } \right]$ is the extracellular sodium concentration, $V_{Na}$ is the reversal potential for sodium ion, and $V_{D}$ is the voltage-defined thermodynamic entity.

13 $$\begin{aligned} \frac{{d\left( {m_{Na} } \right)}}{dt} & = 1.965 \times e^{{\left( {1.7127*V_{D} } \right)}} \times \left( {1 - m_{Na} } \right) \\ & \quad - 0.0424 \times e^{{\left( { - 1.5581*V_{D} } \right)}} \times \left( {m_{Na} } \right), \\ \end{aligned}$$

14 $$\begin{aligned} \frac{{d\left( {h_{Na} } \right)}}{dt} & = 0.00009566 \times e^{{\left( { - 2.4317 \times V_{D} } \right)}} \times \left( {1 - h_{Na} } \right) \\ & \quad - 0.5296 \times e^{{\left( {1.1868 \times V_{D} } \right)}} \times \left( {h_{Na} } \right). \\ \end{aligned}$$

The hyperpolarization-activated cyclic nucleotide (HCN) gated sodium channel current $\left( {I_{NaHCN} } \right)$ is given by,

15 $$I_{NaHCN} \left( V \right) = \left( {\overline{g}_{NaHCN} \times O_{NaHCN} } \right) \times \left( {\sqrt {\left[ {Na_{i} } \right] \times \left[ {Na_{e} } \right]} } \right) \times \left( {\frac{{\sinh \left( {\frac{1}{2} \times \left( {V_{D} - V_{Na} } \right)} \right)}}{{\left( {\frac{{\sinh \left( {\frac{1}{2} \times V_{D} } \right)}}{{\left( {\frac{1}{2} \times V_{D} } \right)}}} \right)}}} \right),$$

where, $\overline{g}_{NaHCN}$ is the maximal conductance for sodium HCN channel, $O_{NaHCN}$ is the gating variable of sodium HCN channel, $\left[ {Na_{i} } \right]$ is the intracellular sodium concentration, $\left[ {Na_{e} } \right]$ is the extracellular sodium concentration, $V_{Na}$ is the reversal potential for sodium ion,$V_{D}$ is the voltage defined thermodynamic entity, and $\left[ {cAMP} \right]$ is the cyclic adenosine monophosphate concentration.

16 $$\frac{{d\left( {O_{NaHCN} } \right)}}{dt} = k_{f,HCN} \times \left( {1 - O_{NaHCN} } \right) - k_{r,HCN} \times O_{NaHCN} ,$$

17 $$k_{f,HCN} = k_{f,free} \times P_{c} + k_{f,bnd} \times \left( {1 - P_{c} } \right),$$

18 $$k_{r,HCN} = k_{r,free} \times P_{o} + k_{r,bnd} \times \left( {1 - P_{o} } \right),$$

19 $$P_{c} = \frac{1}{{\left( {1 + \frac{{\left[ {cAMP} \right]}}{0.001163}} \right)}}; \quad P_{o} = \frac{1}{{\left( {1 + \frac{{\left[ {cAMP} \right]}}{0.0000145}} \right)}},$$

20 $$k_{f,free} = \frac{0.006}{{1 + e^{{\left( {\frac{V + 87.7}{{6.45}}} \right)}} }}; \quad k_{f,bnd} = \frac{0.0268}{{1 + e^{{\left( {\frac{V + 94.2}{{13.3}}} \right)}} }},$$

21 $$k_{r,free} = \frac{0.08}{{1 + e^{{\left( { - \frac{V + 51.7}{7}} \right)}} }}; \quad k_{r,bnd} = \frac{0.08}{{1 + e^{{\left( { - \frac{V + 35.5}{7}} \right)}} }}.$$

The leaky sodium channel current $\left( {I_{Nalk} } \right)$ is given by,

22 $$I_{Nalk} \left( V \right) = \left( {\overline{g}_{Nalk} } \right) \times \left( {\sqrt {\left[ {Na_{i} } \right] \times \left[ {Na_{e} } \right]} } \right) \times \left( {\frac{{\sinh \left( {\frac{1}{2} \times \left( {V_{D} - V_{Na} } \right)} \right)}}{{\left( {\frac{{\sinh \left( {\frac{1}{2} \times V_{D} } \right)}}{{\left( {\frac{1}{2} \times V_{D} } \right)}}} \right)}}} \right),$$

where, $\overline{g}_{Nalk}$ is the maximal conductance for leaky sodium channel, $\left[ {Na_{i} } \right]$ is the intracellular sodium concentration, $\left[ {Na_{e} } \right]$ is the extracellular sodium concentration, $V_{Na}$ is the reversal potential for sodium ion, and $V_{D}$ is the voltage defined thermodynamic entity.

The intracellular potassium concentration dynamics $\left( {\left[ {K_{i} } \right]} \right)$ is given by,

23 $$\frac{{d\left( {\left[ {K_{i} } \right]} \right)}}{dt} = J_{m,K} ,$$

24 $$J_{m,K} = - \frac{1}{{z_{K} \times F \times vol_{cyt} }} \times \left( {I_{KT} - 2 \times I_{NaK} } \right),$$

where, $z_{K}$ is the valence of potassium ion, $I_{KT}$ is the total potassium channel current, $I_{NaK}$ is the ATP-dependent sodium–potassium pump current, $F$ is the Faraday’s constant, and $vol_{cyt}$ is the cytosolic volume.

The total potassium channel current is given by,

25 $$I_{KT} = I_{Kdr} + I_{Kir} + I_{Ksk} ,$$

where, $I_{Kdr}$ is the voltage-dependent (delayed rectifying, DR) potassium channel current, $I_{Kir}$ is the voltage-dependent (inward rectifying, IR) potassium channel current, and $I_{Ksk}$ is the calcium-dependent (small conductance, SK) potassium channel current.

The voltage-dependent (delayed rectifying) potassium channel current $\left( {I_{Kdr} } \right)$ is given by,

26 $$I_{Kdr} \left( V \right) = \left( {\overline{g}_{Kdr} \times O_{Kdr} } \right) \times \left( {V - V_{K} \times V_{\tau } } \right),$$

27 $$O_{Kdr} = m_{Kdr}^{3} ,$$

where, $\overline{g}_{Kdr}$ is the maximal conductance for delayed rectifying potassium channel, $O_{Kdr}$ is the gating variable of voltage-dependent (delayed rectifying) potassium channel, $V_{K}$ is the reversal potential for potassium ion, and $V_{\tau }$ is the temperature defined thermodynamic entity.

28 $$\frac{{d\left( {m_{K,dr} } \right)}}{dt} = \frac{{\frac{1}{{\left( {1 + e^{{\left( { - \frac{{\left( {V + 25} \right)}}{12}} \right)}} } \right)}} - m_{K,dr} }}{{\frac{18}{{\left( {1 + e^{{\left( { - \left[ {\frac{V + 65}{{17.33}}} \right]^{2} } \right)}} } \right)}} + 1}}.$$

The voltage-dependent (inward rectifying) potassium channel current $\left( {I_{Kir} } \right)$ is given by,

29 $$I_{Kir} \left( V \right) = \left( {\overline{g}_{Kir} \times O_{Kir} } \right) \times \left( {V - V_{K} \times V_{\tau } } \right),$$

30 $$O_{Kir} = \frac{1}{{\left( {1 + e^{{\left( {\frac{V + 85}{{12}}} \right)}} } \right)}},$$

where, $\overline{g}_{Kir}$ is the maximal conductance for inward rectifying potassium channel, $O_{Kir}$ is the gating variable of voltage-dependent (inward rectifying) potassium channel, $V_{K}$ is the reversal potential for potassium ion, and $V_{\tau }$ is the temperature defined thermodynamic entity.

The calcium-dependent (small conductance) potassium channel current $\left( {I_{Ksk} } \right)$ is given by,

31 $$I_{Ksk} \left( V \right) = \left( {\overline{g}_{Ksk} \times O_{Ksk} } \right) \times \left( {\sqrt {\left[ {K_{i} } \right] \times \left[ {K_{e} } \right]} } \right) \times \left( {\frac{{\sinh \left( {\frac{1}{2} \times \left( {V_{D} - V_{K} } \right)} \right)}}{{\left( {\frac{{\sinh \left( {\frac{1}{2} \times V_{D} } \right)}}{{\left( {\frac{1}{2} \times V_{D} } \right)}}} \right)}}} \right),$$

32 $$O_{Ksk} = \frac{{\left[ {Ca_{i} } \right]^{4.2} }}{{\left[ {Ca_{i} } \right]^{4.2} + 0.00035^{4.2} }},$$

where, $\overline{g}_{Ksk}$ is the maximal conductance for small conductance potassium channel, $O_{Ksk}$ is the gating variable of calcium-dependent (small conductance) potassium channel, $\left[ {K_{i} } \right]$ is the intracellular potassium concentration, $\left[ {K_{e} } \right]$ is the extracellular potassium concentration, $\left[ {Ca_{i} } \right]$ is the intracellular calcium concentration, $V_{K}$ is the reversal potential for potassium ion, and $V_{D}$ is the voltage defined thermodynamic entity.

The overall synaptic input current flux $\left( {J_{syn} } \right)$ to SNc neuron is given by,

33 $$J_{syn} = - \frac{1}{{F \times vol_{cyt} }} \times \left( {I_{syn}^{ + } + I_{syn}^{ - } - I_{ext} } \right),$$

where, $I_{syn}^{ + }$ is the excitatory synaptic current, $I_{syn}^{ - }$ is the inhibitory synaptic current, $I_{ext}$ is the external current applied, $F$ is the Faraday’s constant, and $vol_{cyt}$ is the cytosolic volume. The different synaptic receptors were modeled similar to Destexhe et al.^20^, and details are specified in Supplementary Material 1.

### Plasma membrane ATPases

The plasma membrane sodium–potassium ATPase $\left( {I_{NaK} } \right)$ is given by,

34 $$I_{NaK} = K_{nak} \times \left[ {k_{1,nak} \times {\mathcal{P}}\left( {E_{1,nak}^{*} } \right) \times y_{nak} - k_{2,nak} \times {\mathcal{P}}\left( {E_{2,nak}^{*} } \right) \times \left( {1 - y_{nak} } \right)} \right],$$

35 $$\frac{{d\left( {y_{nak} } \right)}}{dt} = \beta_{nak} \times \left( {1 - y_{nak} } \right) - \alpha_{nak} \times y_{nak} ,$$

36 $$\beta_{nak} = k_{2,nak} \times {\mathcal{P}}\left( {E_{2,nak}^{*} } \right) + k_{4,nak} \times {\mathcal{P}}\left( {E_{2,nak}^{\# } } \right),$$

37 $$\alpha_{nak} = k_{1,nak} \times {\mathcal{P}}\left( {E_{1,nak}^{*} } \right) + k_{3,nak} \times {\mathcal{P}}\left( {E_{1,nak}^{\# } } \right),$$

38 $${\mathcal{P}}\left( {E_{1,nak}^{*} } \right) = \frac{1}{{\left[ {1 + \frac{{K_{nak,nai} }}{{\left[ {Na_{i} } \right]}} \times \left( {1 + \frac{{\left[ {K_{i} } \right]}}{{K_{nak,ki} }}} \right)} \right]}},$$

39 $${\mathcal{P}}\left( {E_{1,nak}^{\# } } \right) = \frac{1}{{\left[ {1 + \frac{{K_{nak,ki} }}{{\left[ {K_{i} } \right]}} \times \left( {1 + \frac{{\left[ {Na_{i} } \right]}}{{K_{nak,nai} }}} \right)} \right]}},$$

40 $${\mathcal{P}}\left( {E_{2,nak}^{*} } \right) = \frac{1}{{\left[ {1 + \frac{{K_{nak,nae} }}{{Na_{eff} }} \times \left( {1 + \frac{{\left[ {K_{e} } \right]}}{{K_{nak,ke} }}} \right)} \right]}},$$

41 $${\mathcal{P}}\left( {E_{2,nak}^{\# } } \right) = \frac{1}{{\left[ {1 + \frac{{K_{nak,ke} }}{{\left[ {K_{e} } \right]}} \times \left( {1 + \frac{{Na_{eff} }}{{K_{nak,nae} }}} \right)} \right]}},$$

42 $$Na_{eff} = \left[ {Na_{e} } \right] \times e^{{\left( { - 0.82*V_{D} } \right)}} ,$$

43 $$k_{1,nak} = \frac{0.37}{{1 + \frac{0.094}{{\left[ {ATP_{i} } \right]}}}},$$

where, $K_{nak}$ is the maximal conductance for sodium–potassium ATPase, $\left[ {Na_{i} } \right]$ is the intracellular concentration of sodium ion, $\left[ {Na_{e} } \right]$ is the extracellular concentration of sodium ion, $\left[ {K_{i} } \right]$ is the intracellular concentration of potassium ion, $\left[ {K_{e} } \right]$ is the extracellular concentration of potassium ion, $\left( {k_{1,nak} ,k_{2,nak} ,k_{3,nak} ,k_{4,nak} } \right)$ are the reaction rates, $\left( {K_{nak,nae} ,K_{nak,nai} ,K_{nak,ke} ,K_{nak,ki} } \right)$ are the dissociation constants, $\left[ {ATP_{i} } \right]$ is the intracellular concentration of adenosine triphosphate (ATP), and $V_{D}$ is the voltage defined thermodynamic entity.

The plasma membrane calcium ATPase $\left( {I_{pmca} } \right)$ is given by,

44 $$I_{pmca} = K_{pc} \times \left[ {k_{1,pc} \times {\mathcal{P}}\left( {E_{1,pc}^{*} } \right) \times y_{pc} - k_{2,pc} \times {\mathcal{P}}\left( {E_{2,pc}^{*} } \right) \times \left( {1 - y_{pc} } \right)} \right],$$

45 $$\frac{{d\left( {y_{pc} } \right)}}{dt} = \beta_{pc} \times \left( {1 - y_{pc} } \right) - \alpha_{pc} \times y_{pc} ,$$

46 $$\beta_{pc} = k_{2,pc} \times {\mathcal{P}}\left( {E_{2,pc}^{*} } \right) + k_{4,pc} \times {\mathcal{P}}\left( {E_{2,pc} } \right),$$

47 $$\alpha_{pc} = k_{1,pc} \times {\mathcal{P}}\left( {E_{1,pc}^{*} } \right) + k_{3,pc} \times {\mathcal{P}}\left( {E_{1,pc} } \right),$$

48 $${\mathcal{P}}\left( {E_{1,pc}^{*} } \right) = \frac{1}{{\left( {1 + \frac{{K_{pc,i} }}{{\left[ {Ca_{i} } \right]}}} \right)}};\quad {\mathcal{P}}\left( {E_{2,pc}^{*} } \right) = \frac{1}{{\left( {1 + \frac{{K_{pc,e} }}{{\left[ {Ca_{e} } \right]}}} \right)}},$$

49 $${\mathcal{P}}\left( {E_{1,pc} } \right) = 1 - {\mathcal{P}}\left( {E_{1,pc}^{*} } \right);\quad {\mathcal{P}}\left( {E_{2,pc} } \right) = 1 - {\mathcal{P}}\left( {E_{2,pc}^{*} } \right),$$

50 $$k_{1,pc} = \frac{1}{{1 + \frac{0.1}{{\left[ {ATP_{i} } \right]}}}},$$

51 $$K_{pc,i} = \left[ {\frac{173.6}{{1 + \frac{{\left[ {CaCam} \right]}}{{5 \times 10^{ - 5} }}}} + 6.4} \right] \times 10^{ - 5} ,$$

52 $$K_{pc} = k_{pmca} \times \left[ {\frac{{10.56 \times \left[ {CaCam} \right]}}{{\left[ {CaCam} \right] + 5 \times 10^{ - 5} }} + 1.2} \right],$$

where, $\left( {k_{1,pc} ,k_{2,pc} ,k_{3,pc} ,k_{4,pc} } \right)$ are the reaction rates, $k_{pmca}$ is the maximal conductance for calcium ATPase, $\left( {K_{pc,e} ,K_{pc,i} } \right)$ are the dissociation constants, $\left[ {ATP_{i} } \right]$ is the intracellular concentration of ATP, $\left[ {Ca_{i} } \right]$ is the intracellular calcium concentration, and $\left[ {CaCam} \right]$ is the intracellular calcium-bound calmodulin concentration.

### Plasma membrane exchangers

The plasma membrane sodium-calcium exchanger $\left( {I_{NaCaX} } \right)$ is given by,

53 $$I_{NaCaX} = k_{xm} \times \frac{{\left[ {Na_{i} } \right]^{3} \times \left[ {Ca_{e} } \right] \times exp^{{\left( {\delta_{xm} \times V_{D} } \right)}} - \left[ {Na_{e} } \right]^{3} \times \left[ {Ca_{i} } \right] \times e^{{\left( {\left( {\delta_{xm} - 1} \right) \times V_{D} } \right)}} }}{{\left( {1 + {\mathcal{D}}_{xm} \times \left[ {\left[ {Na_{i} } \right]^{3} \times \left[ {Ca_{e} } \right] + \left[ {Na_{e} } \right]^{3} \times \left[ {Ca_{i} } \right]} \right]} \right) \times \left( {1 + \frac{{\left[ {Ca_{i} } \right]}}{0.0069}} \right)}},$$

where, $k_{xm}$ is the maximal conductance for sodium-calcium exchanger, $\left[ {Na_{e} } \right]$ is the extracellular sodium concentration, $\left[ {Na_{i} } \right]$ is the intracellular sodium concentration, $\left[ {Ca_{e} } \right]$ is the extracellular calcium concentration, $\left[ {Ca_{i} } \right]$ is the intracellular calcium concentration, $\delta_{xm}$ is the energy barrier parameter, ${\mathcal{D}}_{xm}$ is the denominator factor, and $V_{D}$ is the voltage defined thermodynamic entity.

### Calcium buffering mechanisms

Intracellular calcium plays an essential role in the normal functioning of the cell. In order to maintain calcium homeostasis, the intracellular calcium levels are tightly regulated by calcium buffering mechanisms such as calcium-binding proteins, endoplasmic reticulum (ER), and mitochondria (MT)^21^ (Supplementary Fig. 2).

The intracellular calcium concentration dynamics $\left( {\left[ {Ca_{i} } \right]} \right)$ after including calcium buffering mechanisms^13,16^ (Fig. 3) is given by,

54 $$\frac{{d\left( {\left[ {Ca_{i} } \right]} \right)}}{dt} = J_{m,Ca} - J_{calb} - 4 \times J_{cam} - J_{serca,er} + J_{ch,er} + J_{leak,er} - J_{mcu,mt} + J_{out,mt} ,$$

where, $J_{m,Ca}$ is the flux of calcium ion channels, $J_{calb}$ is the calcium buffering flux by calbindin, $J_{cam}$ is the calcium buffering flux by calmodulin, $J_{serca,er}$ is the calcium buffering flux by ER uptake of calcium through sarco/endoplasmic reticulum calcium-ATPase (SERCA), $J_{ch,er}$ is the calcium efflux from ER by calcium-induced calcium release (CICR) mechanism, $J_{leak,er}$ is the calcium leak flux from ER, $J_{mcu,mt}$ is the calcium buffering flux by MT uptake of calcium through mitochondrial calcium uniporters (MCUs), and $J_{out,mt}$ is the calcium efflux from MT through sodium-calcium exchangers, mitochondrial permeability transition pores (mPTPs), and non-specific leak flux.

The calcium buffering flux by calbindin $\left( {J_{calb} } \right)$ is given by,

55 $$J_{calb} = k_{1,calb} \times \left[ {Ca_{i} } \right] \times \left[ {Calb} \right] - k_{2,calb} \times \left[ {CaCalb} \right],$$

56 $$\left[ {CaCalb} \right] = \left[ {Calb_{tot} } \right] - \left[ {Calb} \right],$$

57 $$\frac{{d\left( {\left[ {Calb} \right]} \right)}}{dt} = - J_{calb} ,$$

where, $\left( {k_{1,calb} , k_{2,calb} } \right)$ are the calbindin reaction rates, $\left[ {Ca_{i} } \right]$ is the intracellular calcium concentration, $\left[ {Calb} \right]$ is the calbindin concentration, $\left[ {CaCalb} \right]$ is the calcium-bound calbindin concentration, and $\left[ {Calb_{tot} } \right]$ is the total cytosolic calbindin concentration.

The calcium buffering flux by calmodulin $\left( {J_{cam} } \right)$ is given by,

58 $$J_{cam} = \alpha_{cam} \times \left[ {Cam} \right] - \beta_{cam} \times \left[ {CaCam} \right],$$

59 $$\left[ {CaCam} \right] = \left[ {Cam_{tot} } \right] - \left[ {Cam} \right],$$

60 $$\frac{{d\left( {\left[ {Cam} \right]} \right)}}{dt} = - J_{cam} ,$$

61 $$\alpha_{cam} = K_{cam}^{cb} \times K_{cam}^{nb} \times \left[ {\frac{1}{{K_{cam}^{cb} + k_{cam}^{nd} }} + \frac{1}{{k_{cam}^{cd} + k_{cam}^{nd} }}} \right],$$

62 $$\beta_{cam} = k_{cam}^{cd} \times k_{cam}^{nd} \times \left[ {\frac{1}{{K_{cam}^{cb} + k_{cam}^{nd} }} + \frac{1}{{k_{cam}^{cd} + k_{cam}^{nd} }}} \right],$$

63 $$K_{cam}^{cb} = k_{cam}^{cb} \times \left[ {Ca_{i} } \right]^{2} ; \quad K_{cam}^{nb} = k_{cam}^{nb} \times \left[ {Ca_{i} } \right]^{2} ,$$

where, $\left( {k_{cam}^{nd} , k_{cam}^{cd} ,k_{cam}^{cb} ,k_{cam}^{cb} } \right)$ are the calmodulin reaction rates, $\left[ {Ca_{i} } \right]$ is the intracellular calcium concentration, $\left[ {Cam} \right]$ is the calmodulin concentration, $\left[ {CaCam} \right]$ is the calcium-bound calmodulin concentration, and $\left[ {Cam_{tot} } \right]$ is the total cytosolic calmodulin concentration.

The calcium buffering flux by ER uptake of calcium through SERCA $\left( {J_{serca,er} } \right)$ is given by,

64 $$J_{serca,er} = k_{serca,er} \times \left[ {Ca_{i} } \right] \times \left[ {ATP_{i} } \right],$$

where, $k_{serca,er}$ is the maximal rate constant of SERCA, $\left[ {Ca_{i} } \right]$ is the intracellular calcium concentration, and $\left[ {ATP_{i} } \right]$ is the intracellular ATP concentration.

The calcium efflux from ER by CICR $\left( {J_{cicr,er} } \right)$ is given by,

65 $$J_{ch,er} = k_{cicr,er} \times \left( {\frac{{\left[ {Ca_{i} } \right]^{2} }}{{K_{cicr,er}^{2} + \left[ {Ca_{i} } \right]^{2} }}} \right) \times \left( {\left[ {Ca_{er} } \right] - \left[ {Ca_{i} } \right]} \right),$$

where, $k_{ch,er}$ is the maximal permeability of calcium channels in the ER membrane, $K_{ch,er}$ is the half-saturation for calcium, $\left[ {Ca_{i} } \right]$ is the intracellular calcium concentration, and $\left[ {Ca_{er} } \right]$ is the ER calcium concentration.

The calcium leak flux from ER $\left( {J_{leak,er} } \right)$ is given by,

66 $$J_{leak,er} = k_{leak,er} \times \left( {\left[ {Ca_{er} } \right] - \left[ {Ca_{i} } \right]} \right),$$

where, $k_{leak,er}$ is the maximal rate constant for calcium leak flux through the ER membrane, $\left[ {Ca_{i} } \right]$ is the intracellular calcium concentration, and $\left[ {Ca_{er} } \right]$ is the ER calcium concentration.

The ER calcium concentration $\left( {\left[ {Ca_{er} } \right]} \right)$ dynamics is given by,

67 $$\frac{{d\left( {\left[ {Ca_{er} } \right]} \right)}}{dt} = \frac{{\beta_{er} }}{{\rho_{er} }} \times \left( {J_{serca,er} - J_{ch,er} - J_{leak,er} } \right),$$

where, $\beta_{er}$ is the ratio of free calcium to total calcium concentration in the ER, $\rho_{er}$ is the volume ratio between the ER and cytosol, $J_{serca,er}$ is the calcium buffering flux by ER uptake of calcium through SERCA, $J_{ch,er}$ is the calcium efflux from ER by CICR mechanism, and $J_{leak,er}$ is the calcium leak flux from ER.

The calcium buffering flux by MT uptake of calcium through MCUs $\left( {J_{mcu,mt} } \right)$ is given by,

68 $$J_{mcu,mt} = k_{mcu,mt} \times \left( {\frac{{\left[ {Ca_{i} } \right]^{8} }}{{K_{mcu,mt}^{8} + \left[ {Ca_{i} } \right]^{8} }}} \right),$$

where, $k_{mcu,mt}$ is the maximal permeability of mitochondrial membrane calcium uniporters, $K_{mcu,mt}$ is the half-saturation for calcium, and $\left[ {Ca_{i} } \right]$ is the intracellular calcium concentration.

The calcium efflux from MT through sodium-calcium exchangers, mPTPs, and non-specific leak flux $\left( {J_{out,mt} } \right)$ is given by,

69 $$J_{out,mt} = \left( {k_{out,mt} \times \left( {\frac{{\left[ {Ca_{i} } \right]^{2} }}{{K_{out,mt}^{2} + \left[ {Ca_{i} } \right]^{2} }}} \right) + k_{leak,mt} } \right) \times \left[ {Ca_{mt} } \right],$$

where, $k_{out,mt}$ is the maximal rate of calcium flux through sodium-calcium exchangers and mitochondrial permeability transition pores, $K_{out,mt}$ is the half-saturation for calcium, $k_{leak,mt}$ is the maximal rate constant for calcium leak flux through the MT membrane, $\left[ {Ca_{i} } \right]$ is the intracellular calcium concentration, and $\left[ {Ca_{mt} } \right]$ is the MT calcium concentration.

The MT calcium concentration $\left( {\left[ {Ca_{mt} } \right]} \right)$ dynamics is given by,

70 $$\frac{{d\left( {\left[ {Ca_{mt} } \right]} \right)}}{dt} = \frac{{\beta_{mt} }}{{\rho_{mt} }} \times \left( {J_{mcu,mt} - J_{out,mt} } \right),$$

where, $\beta_{mt}$ is the ratio of free calcium to total calcium concentration in the ER, $\rho_{mt}$ is the volume ratio between the MT and cytosol, $J_{mcu,mt}$ is the calcium buffering flux by MT uptake of calcium through MCUs, and $J_{out,mt}$ is the calcium efflux from MT through sodium-calcium exchangers, mPTPs, and non-specific leak flux.

The total instantaneous concentration of calcium $\left( {\left[ {Ca_{tot} } \right]} \right)$ in the SNc cell at a given time $t$ is given by,

71 $$\left[ {Ca_{tot} } \right]\left( t \right) = \left[ {Ca_{i} } \right]\left( t \right) + \frac{{\rho_{er} }}{{\beta_{er} }} \times \left[ {Ca_{er} } \right]\left( t \right) + \frac{{\rho_{mt} }}{{\beta_{mt} }} \times \left[ {Ca_{mt} } \right]\left( t \right) + \left[ {CaCalb} \right]\left( t \right) + \left[ {CaCam} \right]\left( t \right),$$

where, $\beta_{er}$ is the ratio of free calcium to total calcium concentration in the ER, $\rho_{er}$ is the volume ratio between the ER and cytosol, $\beta_{mt}$ is the ratio of free calcium to total calcium concentration in the ER, $\rho_{mt}$ is the volume ratio between the MT and cytosol, $\left[ {Ca_{i} } \right]\left( t \right)$, $\left[ {Ca_{er} } \right]\left( t \right)$, $\left[ {Ca_{mt} } \right]\left( t \right)$, $\left[ {CaCalb} \right]\left( t \right)$, and $\left[ {CaCam} \right]\left( t \right)$ are the instantaneous concentration of intracellular (cytoplasmic) calcium, ER calcium, MT calcium, calcium-bound calbindin, and calcium-bound calmodulin, respectively.

### Energy metabolism pathways

The energy metabolism pathways which were included in the comprehensive model of SNc were adapted from Cloutier & Wellstead energy metabolism model^17^ (Supplementary Fig. 3). Extracellular glucose ($GLC_{e}$) is taken up into the neuron through glucose transporters and phosphorylated into fructose-6-phosphate (F6P) by hexokinase (HK) enzyme using adenosine triphosphate (ATP). F6P is broken down into glyceraldehyde-3-phosphate (GAP) by phosphofructokinase (PFK) enzyme using ATP. At steady state, F6P (fructose-2,6-bisphosphate (F26P)) is phosphorylated (dephosphorylated) to F26P (F6P) by dephosphorylating (phosphorylating) ATP (ADP) using phosphofructokinase-2 (PFK2) enzyme. GAP is dephosphorylated into pyruvate (PYR) by producing ATP using pyruvate kinase (PK). MT produces ATP through oxidative phosphorylation (OP) by utilizing PYR and oxygen ($O_{2}$). Parallel to glycolysis, F6P is utilized to produce Nicotinamide adenine dinucleotide phosphate hydrogen (NADPH) through pentose phosphate pathway. Synthesized NADPH is used to produce glutathione (GSH) by glutathione reductase (GR), which scavenges reactive oxygen species (ROS). ATP is replenished by oxidative phosphorylation independent pathway where phosphocreatine is broken to produce ATP and creatine by creatine kinase (CK).

The following equations give a concise view of all metabolite dynamics in the energy metabolism pathway:

72 $${\text{Fructose - }}6{\text{ - phosphate}}:{ }\frac{{d\left( {\left[ {F6P} \right]} \right)}}{dt} = J_{hk} - \left( {J_{pfk} - V_{pfk2} } \right) - \left( {\frac{1}{6} \times J_{ppp} } \right),$$

73 $${\text{Fructose - }}2,6{\text{ - biphosphate}}:{ }\frac{{d\left( {\left[ {F26P} \right]} \right)}}{dt} = J_{pfk2} ,$$

74 $${\text{Glyceraldehyde - }}3{\text{ - phosphate}}:{ }\frac{{d\left( {\left[ {GAP} \right]} \right)}}{dt} = J_{pfk} - J_{pk} ,$$

75 $${\text{Pyruvate}}:{ }\frac{{d\left( {\left[ {PYR} \right]} \right)}}{dt} = J_{pk} - \left( {J_{op} + J_{ldh} } \right),$$

76 $${\text{Lactate}}:{ }\frac{{d\left( {\left[ {LAC} \right]} \right)}}{dt} = 2.25 \times J_{ldh} + J_{lac} ,$$

77 $${\text{Adenosine}}\,{\text{ triphosphate}}:{ }\frac{{d\left( {\left[ {ATP_{i} } \right]} \right)}}{dt} = \left( {2 \times J_{pk} + 15 \times \eta_{op} \times J_{op} + J_{ck} - \left( {J_{hk} + J_{pfk} + J_{pfk2} + J_{ATPase} } \right)} \right) \times \left( {1 - dAMP\_dATP} \right)^{ - 1} ,$$

78 $${\text{Phosphocreatine}}:{ }\frac{{d\left( {\left[ {PCr} \right]} \right)}}{dt} = - J_{ck} ,$$

79 $${\text{Nicotinamide }}\,{\text{adenine }}\,{\text{dinucleotide }}\,{\text{phosphate }}\,{\text{hydrogen}}\frac{{d\left( {\left[ {NADPH} \right]} \right)}}{dt} = 2 \times J_{ppp} - J_{gr} ,$$

80 $${\text{Glutathione}}:\frac{{d\left( {\left[ {GSH} \right]} \right)}}{dt} = 2 \times J_{gr} - 2 \times J_{dox} ,$$

where, $J_{hk}$ is the irreversible flux of hexokinase enzyme where glucose was phosphorylated to F6P by using ATP, $J_{pfk}$ is the irreversible flux of phosphofructokinase enzyme where F6P was broken down to GAP using ATP, $J_{pfk2}$ is the reversible flux of phosphofructokinase-2 enzyme where F6P (F26P) was phosphorylated (dephosphorylated) to F26P (F6P) by dephosphorylating (phosphorylating) ATP (ADP), $J_{ppp}$ is the irreversible flux of the pentose phosphate pathway where NADP+ was reduced to NADPH, $J_{pk}$ is the irreversible flux of pyruvate kinase enzyme where GAP was dephosphorylated to PYR by phosphorylating adenosine diphosphate (ADP), $J_{op}$ is the irreversible flux of the oxidative phosphorylation pathway where PYR was utilized to produce ATP, $\eta_{op}$ is the electron transport chain efficiency, $J_{ldh}$ is the reversible flux of lactate dehydrogenase where LAC (PYR) was dehydrogenase (hydrogenase) to PYR (LAC), $J_{lac}$ is the reversible flux of monocarboxylate transporters where LAC from extracellular (intracellular) was transported into (out of) the cell, $J_{ck}$ is the reversible flux of creatine kinase where PCr (creatine (Cr)) was dephosphorylated (phosphorylated) to Cr (PCr) by phosphorylating (dephosphorylating) ADP (ATP), $J_{gr}$ is the irreversible flux of glutathione reductase where glutathione disulfide (GSSG) was reduced to GSH, $J_{dox}$ is the irreversible flux of anti-oxidative pathway where reactive oxygen species (ROS) was reduced to water, and $J_{ATPase}$ is the irreversible flux of ATPases where ion equilibrium was maintained by utilizing ATP.

The flux of hexokinase $\left( {J_{hk} } \right)$ is given by,

81 $$J_{hk} = \frac{{\overline{v}_{hk} \times \left[ {GLC_{e} } \right] \times \left( {\frac{{\left[ {ATP_{i} } \right]}}{{\left[ {ATP_{i} } \right] + K_{m,ATP,hk} }}} \right)}}{{\left( {1 + \left( {\frac{{\left[ {F6P} \right]}}{{K_{i,F6P} }}} \right)^{4} } \right)}},$$

where, $\overline{v}_{hk}$ is the maximal hexokinase flux, $\left[ {ATP_{i} } \right]$ is the intracellular ATP concentration, $\left[ {F6P} \right]$ is the F6P concentration, $K_{m,ATP,hk}$ is the affinity constant for ATP, $K_{i,F6P}$ is the inhibition constant for F6P, and $\left[ {GLC_{e} } \right]$ is the extracellular glucose concentration.

The flux of phosphofructokinase $\left( {J_{pfk} } \right)$ is given by,

82 $$J_{pfk} = \overline{v}_{pfk} \times \left( {\frac{{\left[ {F6P} \right]}}{{\left[ {F6P} \right] + K_{m,F6P,pfk} }}} \right) \times \left( {\frac{{\left[ {ATP_{i} } \right]}}{{\left[ {ATP_{i} } \right] + K_{m,ATP,pfk} }}} \right) \times \left( {\frac{{\left[ {F26P} \right]}}{{\left[ {F26P} \right] + K_{m,F26P,pfk} }}} \right) \times ATP_{inh} \times AMP_{act} ,$$

83 $$AMP_{act} = \left( {\frac{{1 + \left( {\frac{{\left[ {AMP} \right]}}{{K_{a,AMP,pfk} }}} \right)}}{{1 + nAMP \times \left( {\frac{{\left[ {AMP} \right]}}{{K_{a,AMP,pfk} }}} \right)}}} \right)^{4} ,$$

84 $$ATP_{inh} = \left( {\frac{{1 + nATP \times \left( {\frac{{\left[ {ATP_{i} } \right]}}{{K_{i,ATP} }}} \right)}}{{1 + \left( {\frac{{\left[ {ATP_{i} } \right]}}{{K_{i,ATP} }}} \right)}}} \right)^{4} ,$$

85 $$\left[ {AMP} \right] = \left[ {ANP} \right] - \left( {\left[ {ATP_{i} } \right] + \left[ {ADP} \right]} \right),$$

86 $$\left[ {ADP} \right] = \left( {\frac{{\left[ {ATP} \right]}}{2}} \right) \times \left( { - Q_{adk} + \sqrt {uADP} } \right),$$

87 $$uADP = Q_{adk}^{2} + 4 \times Q_{adk} \times \left( {\frac{{\left[ {ANP} \right]}}{{\left[ {ATP} \right]}} - 1} \right),$$

88 $$dAMP\_dATP = - 1 + \left( {\frac{{Q_{adk} }}{2}} \right) - \left( {0.5 \times \sqrt {uADP} } \right) + \left( {Q_{adk} \times \frac{ANP}{{\left[ {ATP} \right] \times \sqrt {uADP} }}} \right),$$

where, $\overline{v}_{pfk}$ is the maximal phosphofructokinase flux, $\left[ {ATP_{i} } \right]$ is the intracellular ATP concentration, $\left[ {F6P} \right]$ is the F6P concentration, $\left[ {F26P} \right]$ is the F26P concentration, $K_{m,F6P,pfk}$ is the affinity constant for F6P, $K_{m,ATP,pfk}$ is the affinity constant for ATP, $K_{m,F26P,pfk}$ is the affinity constant for F26P, $\left[ {AMP} \right]$ is the adenosine monophosphate (AMP) concentration, $\left[ {ADP} \right]$ is the adenosine diphosphate (ADP) concentration, $\left[ {ANP} \right]$ is the total energy shuttle’s (ANP) concentration, $K_{a,AMP,pfk}$ is the activation constant for AMP, $K_{i,ATP}$ is the inhibition constant for ATP, $nAMP$ is the coefficient constant for AMP, $nATP$ is the coefficient constant for ATP, and $Q_{adk}$ is the coefficient constant for ADP.

The flux of phosphofructokinase-2 $\left( {J_{pfk2} } \right)$ is given by,

89 $$J_{pfk2} = \overline{v}_{pfk2,f} \times \left( {\frac{{\left[ {F6P} \right]}}{{\left[ {F6P} \right] + K_{m,F6P,pfk2} }}} \right) \times \left( {\frac{{\left[ {ATP_{i} } \right]}}{{\left[ {ATP_{i} } \right] + K_{m,ATP,pfk2} }}} \right) \times AMP_{pfk2} - \overline{v}_{pfk2,r} \times \left( {\frac{{\left[ {F26P} \right]}}{{\left[ {F26P} \right] + K_{m,F26P,pfk2} }}} \right),$$

90 $$AMP_{pfk2} = \frac{{\left( {\frac{{\left[ {AMP} \right]}}{{K_{a,AMP,pfk2} }}} \right)^{2} }}{{1 + \left( {\frac{{\left[ {AMP} \right]}}{{K_{a,AMP,pfk2} }}} \right)^{2} }},$$

where, $\overline{v}_{pfk2,f}$ is the maximal phosphofructokinase-2 forward flux, $\overline{v}_{pfk2,r}$ is the phosphofructokinase-2 maximal reverse flux, $\left[ {ATP_{i} } \right]$ is the intracellular ATP concentration, $\left[ {F6P} \right]$ is the F6P concentration, $\left[ {F26P} \right]$ is the F26P concentration, $\left[ {AMP} \right]$ is the AMP concentration, $K_{m,F6P,pfk2}$ is the affinity constant for F6P, $K_{m,ATP,pfk2}$ is the affinity constant for ATP, $K_{m,F26P,pfk2}$ is the affinity constant for F26P, and $K_{a,AMP,pfk2}$ is the activation constant for AMP.

The flux of pyruvate kinase $\left( {J_{pk} } \right)$ is given by,

91 $$J_{pk} = \overline{v}_{pk} \times \left( {\frac{{\left[ {GAP} \right]}}{{\left[ {GAP} \right] + K_{m,GAP,pk} }}} \right) \times \left( {\frac{{\left[ {ADP} \right]}}{{\left[ {ADP} \right] + K_{m,ADP,pk} }}} \right) \times ATP_{inh} ,$$

where, $\overline{v}_{pk}$ is the pyruvate kinase maximal flux, $\left[ {GAP} \right]$ is the GAP concentration, $\left[ {ADP} \right]$ is the ADP concentration, $K_{m,GAP,pk}$ is the affinity constant for GAP, $K_{m,ADP,pk}$ is the affinity constant for ADP, and $ATP_{inh}$ is the ATP inhibition term.

The flux of the oxidative phosphorylation pathway $\left( {J_{op} } \right)$ is given by,

92 $$J_{op} = \overline{v}_{op} \times \left( {\frac{{\left[ {PYR} \right]}}{{\left[ {PYR} \right] + K_{m,PYR,op} }}} \right) \times \left( {\frac{{\left[ {ADP} \right]}}{{\left[ {ADP} \right] + K_{m,ADP,op} }}} \right) \times \left( {\frac{1}{{1 + 0.1 \times \left( {\frac{{\left[ {ATP_{i} } \right]}}{{\left[ {ADP} \right]}}} \right)}}} \right),$$

where, $\overline{v}_{op}$ is the oxidative phosphorylation pathway maximal flux, $\left[ {PYR} \right]$ is the PYR concentration, $\left[ {ADP} \right]$ is the ADP concentration, $\left[ {ATP_{i} } \right]$ is the ATP concentration, $K_{m,PYR,op}$ is the affinity constant for PYR, and $K_{m,ADP,op}$ is the affinity constant for ADP.

In the absence of protein aggregation, the electron transport chain efficiency is given by,

93 $$\eta_{op} = \overline{\eta }_{op} .$$

Moreover, in the presence of protein aggregation, the electron transport chain efficiency is given by,

94 $$\eta_{op} = \overline{\eta }_{op} - \beta_{{op,asyn_{mis} }} \times \left( {\frac{1}{{1 + \left( {\frac{{K_{{asyn_{mis} }} }}{{\left[ {ASYN_{mis} } \right]}}} \right)^{4} }}} \right),$$

where, $\overline{\eta }_{op}$ is the maximal electron transport chain efficiency, $\beta_{{op,asyn_{mis} }}$ is the maximum fractional decrease in the oxidative phosphorylation efficiency through misfolded alpha-synuclein $\left( {ASYN_{mis} } \right)$, $\left[ {ASYN_{mis} } \right]$ is the misfolded alpha-synuclein concentration, and $K_{{asyn_{mis} }}$ is the threshold concentration for mitochondrial damage by $ASYN_{mis}$.

The flux of lactate dehydrogenase $\left( {J_{ldh} } \right)$ is given by,

95 $$J_{ldh} = \eta_{ldh} \times \left( {k_{ldh,f} \times \left[ {PYR} \right] - k_{ldh,r} \times \left[ {LAC} \right]} \right),$$

where, $\eta_{ldh}$ is the lactate fermentation efficiency, $\left[ {PYR} \right]$ is the PYR concentration, $\left[ {LAC} \right]$ is the LAC concentration, $k_{ldh,f}$ is the forward reaction constant of lactate dehydrogenase (LDH), and $k_{ldh,r}$ is the reverse reaction constant of lactate dehydrogenase.

In the absence of oxidative stress, the lactate fermentation efficiency is given by,

96 $$\eta_{ldh} = \overline{\eta }_{ldh} ,$$

Moreover, in the presence of oxidative stress, the lactate fermentation efficiency is given by,

97 $$\eta_{ldh} = \overline{\eta }_{ldh} - \beta_{ldh,ROS} \times \left( {\frac{1}{{1 + \left( {\frac{{K_{ldh,ROS} }}{{\left[ {ROS} \right]}}} \right)^{4} }}} \right),$$

where, $\overline{\eta }_{ldh}$ is the maximal lactate fermentation efficiency, $\beta_{ldh,ROS}$ is the maximum fractional decrease in the lactate fermentation efficiency through reactive oxygen species $\left( {ROS} \right)$, $K_{ldh,ROS}$ is the threshold concentration for lactate fermentation damage by $\left[ {ROS} \right]$, and $\left[ {ROS} \right]$ is the ROS concentration.

The flux of monocarboxylate transporters $\left( {J_{lac} } \right)$ is given by,

98 $$J_{lac} = \overline{v}_{lac} \times \left( {1 + v_{stim} \times K_{lac,inf} } \right) - K_{lac,eff} \times \left[ {LAC} \right],$$

where, $\overline{v}_{lac}$ is the monocarboxylate transporters (MCTs) maximal inward flux, $\left[ {LAC} \right]$ is the LAC concentration, $v_{stim}$ is the stimulation pulse, $K_{lac,inf}$ is the coefficient constant for the inward flux of MCT, $K_{lac,eff}$ is the reaction constant for lactate efflux.

The flux of ATPases $\left( {J_{ATPase} } \right)$ is given by,

99 $$J_{ATPase} = \overline{v}_{ATPase} \times \left( {\frac{{\left[ {ATP_{i} } \right]}}{{\left[ {ATP_{i} } \right] + K_{m,ATP} }}} \right) \times \left( {1 + v_{stim} } \right),$$

where, $\overline{v}_{ATPase}$ is the ATPase maximal flux, $\left[ {ATP_{i} } \right]$ is the intracellular ATP concentration, $K_{m,ATP}$ is the affinity constant for ATP, and $v_{stim}$ is the stimulation pulse.

The flux of the pentose phosphate pathway $\left( {J_{ppp} } \right)$ is given by,

100 $$J_{ppp} = \overline{v}_{ppp} \times \frac{{\left( {\frac{{\left[ {F6P} \right]}}{{\left[ {F6P} \right] + K_{m,F6P,pfk} }}} \right)}}{{\left( {1 + \left( {\frac{{\left( {\frac{{\left[ {NADPH} \right]}}{{\left[ {NADP} \right]}}} \right)}}{{K_{i,NADPH} }}} \right)} \right)}},$$

101 $$\left[ {NADP} \right] = \left[ {NADPH_{tot} } \right] - \left[ {NADPH} \right],$$

where, $\overline{v}_{ppp}$ is the pentose phosphate pathway (PPP) maximal flux, $\left[ {F6P} \right]$ is the F6P concentration, $\left[ {NADPH} \right]$ is the NADPH concentration, $\left[ {NADP} \right]$ is the nicotinamide adenine dinucleotide phosphate (NADP) concentration, $\left[ {NADPH_{tot} } \right]$ is the total NADPH and NADP concentration, $K_{m,F6P,pfk}$ is the affinity constant for F6P, and $K_{i,NADPH}$ is the inhibition constant of PPP by NADPH to NADP ratio.

The flux of glutathione reductase $\left( {J_{gr} } \right)$ is given by,

102 $$J_{gr} = k_{gr,f} \times \left[ {GSSG} \right] \times \left[ {NADPH} \right] - k_{gr,r} \times \left[ {GSH} \right] \times \left[ {NADP} \right],$$

103 $$\left[ {GSSG} \right] = \left[ {GSH_{tot} } \right] - \left[ {GSH} \right],$$

where, $k_{gr,f}$ is the forward reaction constant of glutathione reductase, $k_{gr,r}$ is the reverse reaction constant of glutathione reductase, $\left[ {NADPH} \right]$ is the NADPH concentration, $\left[ {NADP} \right]$ is the NADP concentration, $\left[ {GSH} \right]$ is the GSH concentration, $GSSG$ is the GSSG concentration, and $\left[ {GSH_{tot} } \right]$ is the total GSH and GSSG concentration together.

The flux of anti-oxidative pathway $\left( {J_{dox} } \right)$ is given by,

104 $$J_{dox} = K_{dox,ROS} \times \left[ {GSH} \right] \times \left[ {ROS} \right],$$

where, $K_{dox,ROS}$ is the reaction constant for ROS reduction by glutathione, $\left[ {GSH} \right]$ is the GSH concentration, and $\left[ {ROS} \right]$ is the ROS concentration.

The flux of creatine kinase $\left( {J_{ck} } \right)$ is given by,

105 $$J_{ck} = \left( {k_{ck,f} \times \left[ {PCr} \right] \times \left[ {ADP} \right]} \right) - \left( {k_{ck,r} \times \left[ {Cr} \right] \times \left[ {ATP_{i} } \right]} \right),$$

106 $$\left[ {Cr} \right] = \left[ {PCr_{tot} } \right] - \left[ {PCr} \right],$$

where, $k_{ck,f}$ is the forward reaction constant of creatine kinase, $k_{ck,r}$ is the reverse reaction constant of creatine kinase, $\left[ {PCr} \right]$ is the PCr concentration, $\left[ {Cr} \right]$ is the Cr concentration, $\left[ {PCr_{tot} } \right]$ is the total PCr and Cr concentration, $\left[ {ADP} \right]$ is the ADP concentration, and $\left[ {ATP_{i} } \right]$ is the intracellular ATP concentration.

### Dopamine turnover processes

The DA turnover process has been modelled as a three-compartment biochemical model based on Michaelis–Menten kinetics^9^. The three compartments are intracellular compartment representing cytosol, extracellular compartment representing extracellular space (ECS), and vesicular compartment representing a vesicle. Previously published dopaminergic terminal models are specified in Supplementary Table 2. In DA turnover processes, l-tyrosine (TYR) is converted into l-3,4-dihydroxyphenylalanine or L-DOPA by tyrosine hydroxylase (TH), which in turn is converted into DA by aromatic l-amino acid decarboxylase (AADC) (Supplementary Fig. 4.1). The cytoplasmic DA ($DA_{c}$) is stored into vesicles by vesicular monoamine transporter 2 (VMAT-2) (Supplementary Fig. 4.2). Upon arrival of action potential, vesicular DA ($DA_{v}$) is released into extracellular space (Supplementary Fig. 4.3). Most of the extracellular DA ($DA_{e}$) is taken up into the terminal through DA plasma membrane transporter (DAT) (Supplementary Fig. 4.4) and remaining extracellular DA is metabolized by catechol-*O*-methyltransferase (COMT) and monoamine oxidase (MAO) into homovanillic acid (HVA) (Supplementary Fig. 4.5). The DA that enters the terminal is again packed into vesicles, and the remaining cytoplasmic DA is metabolized by COMT and MAO enzymes (Supplementary Fig. 4.5). It is known that a DA neuron self-regulates its firing, neurotransmission and synthesis by autoreceptors^22,23^. In the present model, we included autoreceptors that regulate the synthesis and release of DA (Supplementary Figs. 4.6, 4.7). Along with TYR, external L-DOPA compete for transporting into the terminal through aromatic L-amino acid transporter (AAT) (Supplementary Fig. 4.8).

#### Modelling extracellular DA in the ECS

The major three mechanisms that determine the dynamics of extracellular DA $\left( {\left[ {DA_{e} } \right]} \right)$ in the ECS given by,

107 $$\frac{{d\left( {\left[ {DA_{e} } \right]} \right)}}{dt} = J_{rel} - J_{DAT} - J_{eda}^{o} ,$$

where, $J_{rel}$ represents the flux of calcium-dependent DA release from the DA terminal, $J_{DAT}$ represents the unidirectional flux of DA translocated from the extracellular compartment (ECS) into the intracellular compartment (cytosol) via DA plasma membrane transporter (DAT), and $J_{eda}^{o}$ represents the outward flux of DA degradation, which clears DA from ECS.

#### Calcium-dependent DA release flux

Assuming that calcium-dependent DA release occurs within less than a millisecond after the calcium channels open, the flux of DA release $\left( {J_{rel} } \right)$ from the DA terminal is given by,

108 $$J_{rel} = \psi \times n_{RRP} \times P_{rel} \left( {\left[ {Ca_{i} } \right]} \right),$$

where, $\left[ {Ca_{i} } \right]$ is the intracellular calcium concentration in the DA terminal, $P_{rel}$ is the release probability as a function of intracellular calcium concentration, $n_{RRP}$ is the average number of readily releasable vesicles, and $\psi$ is the average release flux per vesicle within a single synapse.

The flux of calcium-dependent DA release depends on extracellular DA concentration, and intracellular ATP acts as a feedback mechanism, assuming this regulation as extracellular DA and intracellular ATP controls the number of vesicles in the readily releasable vesicle pool $\left( {n_{RRP} } \right)$ which is given by,

109 $$n_{RRP} = \frac{{\eta_{nrrp} \times e^{{\left( {\frac{{\left[ {ATP_{i} } \right]}}{{K_{a,RRP} }}} \right)}} }}{{\left( {1 + e^{{\left[ {\frac{{ - \left( {\left[ {DA_{v} } \right] - \left[ {DA_{{v_{o} }} } \right]} \right)}}{{DA_{{v_{s} }} }}} \right]}} } \right) \times \left( {1 + e^{{\left[ {\frac{{\left[ {DA_{e} } \right] - DA_{{R_{a} }} }}{{DA_{{R_{s} }} }}} \right]}} } \right)}},$$

110 $$\eta_{nrrp} = \overline{\eta }_{nrrp} - \beta_{{nrrp,asyn_{mis} }} \times \left( {\frac{1}{{1 + \left( {\frac{{K_{{asyn_{mis} }} }}{{\left[ {ASYN_{mis} } \right]}}} \right)^{4} }}} \right),$$

where, $\left[ {DA_{{v_{o} }} } \right]$ is the initial vesicular DA concentration, $DA_{{v_{s} }}$ is the sensitivity to vesicular concentration, $DA_{{R_{a} }}$ is the high-affinity state for DA binding to receptors and $DA_{{R_{s} }}$ is the binding sensitivity, $\left[ {ATP_{i} } \right]$ is the intracellular ATP concentration, $K_{a,RRP}$ is the activation constant for ATP, $\eta_{nrrp}$ is the effect of misfolded alpha-synuclein on vesicle recycling^24^, $\overline{\eta }_{nrrp}$ is the maximal vesicle recycling efficiency, $\beta_{{nrrp,asyn_{mis} }}$ is the maximum fractional decrease in the vesicle recycling efficiency through $ASYN_{mis}$, $K_{{asyn_{mis} }}$ is the threshold concentration for damage by $ASYN_{mis}$, and $\left[ {ASYN_{mis} } \right]$ is the misfolded alpha-synuclein concentration.

The release probability of DA as a function of intracellular calcium concentration is given by,

111 $$P_{rel} \left( {\left[ {Ca_{i} } \right]} \right) = \overline{P}_{rel} \times \frac{{\left[ {Ca_{i} } \right]^{4} }}{{\left[ {Ca_{i} } \right]^{4} + K_{rel}^{4} }},$$

where, $\overline{P}_{rel}$ is the maximum release probability and $K_{rel}$ is the sensitivity of calcium concentration, and $\left[ {Ca_{i} } \right]$ is the intracellular calcium concentration.

#### Unidirectional reuptake flux of DA

The unidirectional reuptake flux of extracellular DA into the presynaptic terminal is given by,

112 $$J_{DAT} = \overline{V}_{eda} \times \frac{{\left[ {DA_{e} } \right]}}{{K_{eda} + \left[ {DA_{e} } \right]}},$$

where, $\overline{V}_{eda}$ is the maximal velocity of DA transporter (DAT), $K_{eda}$ is the DA concentration at half-maximal velocity, and $\left[ {DA_{e} } \right]$ is the extracellular DA concentration.

#### Outward extracellular flux

The flux of extracellular DA enzymatic degradation in the synaptic cleft (ECS) is given by,

113 $$J_{eda}^{o} = k_{comt} \times \left[ {DA_{e} } \right],$$

where, $k_{comt}$ is the rate at which extracellular DA cleared from ECS, and $\left[ {DA_{e} } \right]$ is the extracellular DA concentration.

##### Modelling intracellular DA in the terminal

The intracellular DA dynamics $\left( {\left[ {DA_{i} } \right]} \right)$ is determined as the sum of DA concentration in cytosolic and vesicular compartments and is given by,

114 $$\frac{{d\left( {\left[ {DA_{i} } \right]} \right)}}{dt} = \frac{{d\left( {\left[ {DA_{c} } \right]} \right)}}{dt} + \frac{{d\left( {\left[ {DA_{v} } \right]} \right)}}{dt}.$$

The cytosolic DA dynamics $\left( {\left[ {DA_{c} } \right]} \right)$ is given by,

115 $$\frac{{d\left( {\left[ {DA_{c} } \right]} \right)}}{dt} = J_{DAT} - J_{VMAT} - J_{cda}^{o} + J_{ldopa} ,$$

where, $J_{DAT}$ represents the unidirectional flux of DA translocated from ECS into the cytosol through DAT, $J_{VMAT}$ represents the flux of cytosolic DA into vesicle through VMAT-2, $J_{ida}^{o}$ represents the outward flux of DA degradation, which clears DA from the cytosol, and $J_{ldopa}$ represents the flux of synthesized cytosol DA from L-DOPA.

The vesicular DA dynamics $\left( {\left[ {DA_{v} } \right]} \right)$ is given by,

116 $$\frac{{d\left( {\left[ {DA_{v} } \right]} \right)}}{dt} = J_{VMAT} - J_{rel} ,$$

where, $J_{rel}$ represents the flux of calcium-dependent DA release from the DA terminal, $J_{VMAT}$ represents the flux of cytosolic DA into a vesicle.

#### L-DOPA synthesis flux

The flux of synthesized L-DOPA whose velocity is the function of intracellular calcium concentration and L-DOPA synthesis is regulated by the substrate (TYR) itself, extracellular DA (via autoreceptor) and intracellular DA concentrations are given by,

117 $$J_{synt} = \frac{{V_{synt} }}{{1 + \frac{{K_{TYR} }}{{\left[ {TYR} \right]}} \times \left( {1 + \frac{{\left[ {DA_{c} } \right]}}{{K_{i,cda} }} + \frac{{\left[ {DA_{e} } \right]}}{{K_{i,eda} }}} \right)}},$$

where, $V_{synt}$ is the velocity of synthesizing L-DOPA, $\left[ {TYR} \right]$ is the tyrosine concentration in terminal bouton, $K_{TYR}$ is the tyrosine concentration at which half-maximal velocity was attained, $K_{i,cda}$ is the inhibition constant on $K_{TYR}$ due to cytosolic DA concentration, $K_{i,eda}$ is the inhibition constant on $K_{TYR}$ due to extracellular DA concentration, $\left[ {DA_{c} } \right]$ is the cytoplasmic DA concentration, and $\left[ {DA_{e} } \right]$ is the extracellular DA concentration.

In Chen et al.^25^, neuronal stimulation was linked to DA synthesis through an indirect event, which starts with calcium influx into the terminal bouton. In this model, the velocity of L-DOPA synthesis as a function of calcium levels in the terminal bouton is expressed as,

118 $$V_{synt} \left( {Ca_{i} } \right) = \overline{V}_{synt} \times \frac{{\left[ {Ca_{i} } \right]^{4} }}{{K_{synt}^{4} + \left[ {Ca_{i} } \right]^{4} }},$$

where, $K_{synt}$ is the calcium sensitivity, $\overline{V}_{synt}$ is the maximal velocity for L-DOPA synthesis, and $\left[ {Ca_{i} } \right]$ is the intracellular calcium concentration.

#### Storage flux of DA into the vesicle

The flux of transporting DA in the cytosol into the vesicles, which depends on the intracellular ATP is given by,

119 $$J_{VMAT} = V_{cda,ATP} \times \frac{{\left[ {DA_{c} } \right]}}{{K_{cda} + \left[ {DA_{c} } \right]}},$$

120 $$V_{cda,ATP} = \overline{V}_{cda} \times \alpha_{vmat} \times e^{{\left( {\beta_{vmat} \times \left[ {ATP_{i} } \right]} \right)}} ,$$

where, $K_{cda}$ is the cytosolic DA concentration at which half-maximal velocity was attained, $\overline{V}_{cda}$ is the maximal velocity with which DA was packed into vesicles, $\left[ {DA_{c} } \right]$ is the cytosolic DA concentration, $\alpha_{vmat}$ is the scaling factor for VMAT-2, $\beta_{vmat}$ is the scaling factor for $ATP_{i}$, and $\left[ {ATP_{i} } \right]$ is the intracellular ATP concentration.

#### Outward intracellular flux

The flux of intracellular DA enzymatic degradation in synaptic bouton (cytosol) is given by,

121 $$J_{cda}^{o} = k_{mao} \times \left[ {DA_{c} } \right]$$

where, $k_{mao}$ is the rate at which intracellular DA cleared from the cytosol, and $\left[ {DA_{c} } \right]$ is the cytosolic DA concentration.

#### L-DOPA to DA conversion flux

The flux of L-DOPA conversion to DA by AADC^12^ is given by,

122 $$J_{ldopa} = \overline{V}_{aadc} \times \frac{{\left[ {LDOPA} \right]}}{{K_{aadc} + \left[ {LDOPA} \right]}},$$

where, $K_{aadc}$ is the L-DOPA concentration at which half-maximal velocity was attained, $\overline{V}_{aadc}$ is the maximal velocity with which L-DOPA was converted to DA, $\left[ {LDOPA} \right]$ is the L-DOPA concentration.

#### Transport flux of exogenous L-DOPA into the terminal

The flux of exogenous L-DOPA transported into the terminal through AAT while competing with other aromatic amino acids^12^ is given by,

123 $$J_{aat} = \overline{V}_{aat} \times \frac{{\left[ {LDOPA_{e} } \right]}}{{\left( {K_{{ldopa_{e} }} \times \left( {1 + \left( {\frac{{\left[ {TYR_{e} } \right]}}{{K_{{tyr_{e} }} }}} \right) + \left( {\frac{{\left[ {TRP_{e} } \right]}}{{K_{{trp_{e} }} }}} \right)} \right) + \left[ {LDOPA_{e} } \right]} \right)}},$$

where, $K_{{ldopa_{e} }}$ is the extracellular L-DOPA concentration at which half-maximal velocity was attained, $\overline{V}_{aat}$ is the maximal velocity with which extracellular L-DOPA was transported into the cytosol, $\left[ {LDOPA_{e} } \right]$ is the extracellular L-DOPA concentration, $\left[ {TYR_{e} } \right]$ is the extracellular TYR concentration, $\left[ {TRP_{e} } \right]$ is the extracellular tryptophan (TRP) concentration, $K_{{tyr_{e} }}$ is the affinity constant for $\left[ {TYR_{e} } \right]$, $K_{{trp_{e} }}$ is the affinity constant for $\left[ {TRP_{e} } \right]$.

When L-DOPA drug therapy is initiated,

124 $$\left[ {LDOPA_{e} } \right] = \left[ {sLD} \right].$$

When no L-DOPA drug therapy is initiated,

125 $$LDOPA_{e} = 0.$$

The L-DOPA concentration $\left( {\left[ {LDOPA} \right]} \right)$ dynamics inside the terminal is given by,

126 $$\frac{{d\left( {\left[ {LDOPA} \right]} \right)}}{dt} = J_{aat} - J_{ldopa} + J_{synt} ,$$

where, $J_{aat}$ represents the flux of exogenous L-DOPA transported into the cytosol, $J_{ldopa}$ represents the conversion flux of exogenous L-DOPA into DA, $J_{synt}$ represents the flux of synthesized LDOPA from tyrosine, and $\left[ {sLD} \right]$ is the serum L-DOPA concentration.

### Molecular pathways involved in PD pathology

The molecular pathways in PD pathology were adapted from Cloutier & Wellstead model^10^ and incorporated in the comprehensive model of SNc cell. ROS formation occurs due to leakage from mitochondria during oxidative phosphorylation for ATP production, auto-oxidation of excess freely available DA in the cytoplasm, and misfolded alpha-synuclein ($ASYN_{mis}$). In the present model, excess ROS is scavenged by glutathione. Under pathological conditions such as elevated ROS levels, normal alpha-synuclein ($ASYN$) undergoes conformation changes into misfolded alpha-synuclein. The misfolded alpha-synuclein is tagged ($ASYN_{tag}$) and degraded by the ubiquitous-proteasome pathway using ATP. Excess misfolded alpha-synuclein forms aggregates, which in turn gets degraded by the lysosomal degradation pathway using ATP. In some scenarios, these alpha-synuclein aggregates ($ASYN_{agg}$) form Lewy bodies ($LBs$) (Supplementary Fig. 5).

The model consists of ROS formation from different processes, including ROS scavenging mechanism, alpha-synuclein aggregation, proteasomal and lysosomal degradation of damaged protein, etc. The following equations give a concise view of all metabolite dynamics in the PD pathology pathways,

127 $${\text{Reactive }}\,{\text{oxygen }}\,{\text{species}}:{ }\frac{{d\left( {\left[ {ROS} \right]} \right)}}{dt} = J_{leak} + J_{env} + J_{dopa} - J_{cat} - J_{dox} ,$$

128 $${\text{Alpha - synuclein}}:{ }\frac{{d\left( {\left[ {ASYN} \right]} \right)}}{dt} = J_{syn} - J_{ox} - J_{to} ,$$

129 $${\text{Misfolded}}\,{\text{ alpha - synuclein}}:{ }\frac{{d\left( {\left[ {ASYN_{mis} } \right]} \right)}}{dt} = J_{ox} - J_{agg} - J_{tag} ,$$

130 $${\text{Tagged alpha - synuclein}}:{ }\frac{{d\left( {\left[ {ASYN_{tag} } \right]} \right)}}{dt} = J_{tag} - J_{prt} ,$$

131 $${\text{Aggregated alpha - synuclein}}:{ }\frac{{d\left( {\left[ {ASYN_{agg} } \right]} \right)}}{dt} = J_{agg} - J_{lyso} - J_{lb} ,$$

132 $${\text{Lewy}}\,{\text{ bodies}}:{ }\frac{{d\left( {\left[ {LB} \right]} \right)}}{dt} = J_{lb} ,$$

where, $J_{leak}$ is the flux of oxidative stress due to mitochondrial leakage, $J_{env}$ is the flux of external oxidative stress (includes environmental toxins, inflammatory responses, etc.), $J_{dopa}$ is the flux of oxidative stress due to excess cytoplasmic DA, $J_{cat}$ is the catabolizing flux of ROS by catalase enzyme, $J_{dox}$ is the flux of GSH-dependent ROS scavenging pathway (Eq. 104), $J_{syn}$ is the synthesizing flux of alpha-synuclein protein, $J_{ox}$ is the flux of alpha-synuclein misfolding due to ROS, $J_{to}$ is the usage flux of alpha-synuclein in other processes, $J_{agg}$ is the flux of alpha-synuclein aggregation, $J_{tag}$ is the flux of ATP-dependent ubiquitination of damaged protein for proteasomal degradation, $J_{prt}$ is the flux of ATP-dependent breakdown of damaged protein through proteasomal degradation, $J_{lyso}$ is the flux of ATP-dependent breakdown of aggregated protein through lysosomal degradation, and $J_{lb}$ is the flux of LBs formation.

The flux of oxidative stress due to mitochondrial leakage $\left( {J_{leak} } \right)$ is given by,

133 $$J_{leak} = \left( {\frac{{K_{a,leak} }}{{\left[ {ATP_{i} } \right]}}} \right) \times \left( {1 - \eta_{op} } \right) \times J_{op} ,$$

where, $J_{op}$ is the flux of the oxidative phosphorylation pathway, $\eta_{op}$ is the electron transport chain efficiency, $\left[ {ATP_{i} } \right]$ is the intracellular ATP concentration, and $K_{a,ATP}$ is the activation constant for ATP.

The flux of oxidative stress due to excess DA in the cytoplasm $\left( {J_{dopa} } \right)$ is given by,

134 $$J_{dopa} = k_{dopa} \times \frac{{\left[ {DA_{c} } \right]}}{{\left[ {DA_{c} } \right] + \left[ {K_{dopa} } \right]}},$$

where, $k_{dopa}$ is the reaction constant for ROS production by excess DA, $\left[ {DA_{c} } \right]$ is the cytoplasmic DA concentration, and $K_{dopa}$ is the affinity constant for $\left[ {DA_{c} } \right]$.

The catabolizing flux of ROS by catalase enzyme $\left( {J_{cat} } \right)$ is given by,

135 $$J_{cat} = k_{cat} \times \left[ {ROS} \right],$$

where, $k_{cat}$ is the reaction constant for catalase, and $\left[ {ROS} \right]$ is the ROS concentration.

The synthesizing flux of alpha-synuclein protein $\left( {J_{syn} } \right)$ is given by,

136 $$J_{syn} = k_{syn} ,$$

where, $k_{syn}$ is the reaction constant for alpha-synuclein synthesis.

The flux of alpha-synuclein misfolding due to ROS $\left( {J_{ox} } \right)$ is given by,

137 $$J_{ox} = k_{ox} \times \left[ {ASYN} \right] \times \left[ {ROS} \right],$$

where, $k_{ox}$ is the reaction constant for alpha-synuclein oxidation, $\left[ {ASYN} \right]$ is the alpha-synuclein concentration, and $\left[ {ROS} \right]$ is the ROS concentration.

The usage flux of alpha-synuclein in other processes $\left( {J_{to} } \right)$ is given by,

138 $$J_{to} = k_{to} \times \left[ {ASYN} \right],$$

where, $k_{to}$ is the reaction constant for alpha-synuclein consumption, and $\left[ {ASYN} \right]$ is the alpha-synuclein concentration.

The flux of alpha-synuclein aggregation $\left( {J_{agg} } \right)$ is given by,

139 $$J_{agg} = k_{agg} \times \left[ {ASYN_{mis} } \right] \times \frac{{\left[ {ASYN_{mis} } \right]^{6} }}{{\left[ {ASYN_{mis} } \right]^{6} + K_{agg}^{6} }},$$

where, $k_{agg}$ is the reaction constant for alpha-synuclein aggregation, $\left[ {ASYN_{mis} } \right]$ is the misfolded alpha-synuclein concentration, and $K_{agg}$ is the affinity constant for $\left[ {ASYN_{mis} } \right]$.

The flux of ATP-dependent ubiquitination of damaged protein for proteasomal degradation $\left( {J_{tag} } \right)$ is given by,

140 $$J_{tag} = k_{tag} \times ASYN_{mis} \times \left[ {Ub} \right] \times \left[ {ATP_{i} } \right],$$

141 $$\left[ {Ub} \right] = \left[ {Ub_{tot} } \right] - \left[ {ASYN_{tag} } \right],$$

where, $k_{tag}$ is the reaction constant for ubiquitination of damaged protein, $\left[ {ASYN_{mis} } \right]$ is the misfolded alpha-synuclein concentration, $\left[ {Ub} \right]$ is the ubiquitin concentration, $\left[ {ATP_{i} } \right]$ is the intracellular ATP concentration, $\left[ {Ub_{tot} } \right]$ is the total ubiquitin concentration, and $\left[ {ASYN_{tag} } \right]$ is the tagged alpha-synuclein concentration.

The flux of ATP-dependent breakdown of damaged protein through proteasomal degradation $\left( {J_{prt} } \right)$ is given by,

142 $$J_{prt} = k_{prt} \times \left[ {ASYN_{tag} } \right] \times \left[ {ATP_{i} } \right] \times \left( {1 - \beta_{prt} \times \left( {\frac{{\left[ {ASYN_{agg} } \right]^{4} }}{{\left[ {ASYN_{agg} } \right]^{4} + K_{prt}^{4} }}} \right)} \right),$$

where, $k_{prt}$ is the reaction constant for damaged protein disposal by the proteasome, $\left[ {ASYN_{tag} } \right]$ is the tagged alpha-synuclein concentration, $\left[ {ATP_{i} } \right]$ is the intracellular ATP concentration, $\left[ {ASYN_{agg} } \right]$ is the aggregated alpha-synuclein concentration, $K_{prt}$ is the affinity constant for $\left[ {ASYN_{agg} } \right]$, and $\beta_{prt}$ is the fraction reduction of proteasome activity by $\left[ {ASYN_{agg} } \right]$.

The flux of ATP-dependent breakdown of aggregated protein through lysosomal degradation $\left( {J_{lyso} } \right)$ is given by,

143 $$J_{lyso} = k_{lyso} \times \left[ {ASYN_{agg} } \right] \times \left[ {ATP_{i} } \right],$$

where, $k_{lyso}$ is the reaction constant for $\left[ {ASYN_{agg} } \right]$ disposal by the lysosome, and $\left[ {ATP_{i} } \right]$ is the intracellular ATP concentration.

The flux of LB formation $\left( {J_{lb} } \right)$ is given by,

144 $$V_{lb} = k_{lb} \times \left[ {ASYN_{agg} } \right] \times \frac{{\left[ {ASYN_{agg} } \right]^{6} }}{{\left[ {ASYN_{agg} } \right]^{6} + K_{lb}^{6} }},$$

where, $k_{lb}$ is the reaction constant for Lewy bodies from $\left[ {ASYN_{agg} } \right]$, $\left[ {ASYN_{agg} } \right]$ is the aggregated alpha-synuclein concentration, and $K_{prt}$ is the affinity constant for $\left[ {ASYN_{agg} } \right]$.

### Apoptotic pathways

The apoptotic pathways were adapted from Hong et al.^18^ and incorporated in the comprehensive model of SNc cell. The model consists of ER stress-induced apoptotic activation and mitochondrial ROS-induced apoptotic activation^26^ (Supplementary Fig. 6).

Under stress conditions, calcium from ER efflux and intracellular calcium $\left( {Ca_{i} } \right)$ builds up in the cytoplasm of SNc neurons, which activates calcium-dependent calpain $\left( {Calpain} \right)$ protease through ER stress-induced pathway^27^. Activated calpain $\left( {Calpain^{*} } \right)$ proteases procaspase-12 $\left( {Casp12} \right)$ to caspase-12 $\left( {Casp12^{*} } \right)$ through calpain-dependent activation of caspase-12^28^. Activated caspase-12 cleaves procaspase-9 $\left( {Casp9} \right)$ into caspase-9 $\left( {Casp9^{*} } \right)$ through cytochrome c-independent pathway^29^, caspase-9, in turn, activates procaspase-3 $\left( {Casp3} \right)$ into caspase-3 $\left( {Casp3^{*} } \right)$^30^. Activated caspase-3 eventually induces apoptotic mediators $\left( {Apop} \right)$^31^.

Under stress conditions, the mitochondrial permeability increases through mitochondrial permeability transition pore complex $\left( {PTP_{mit} } \right),$ which leads to release of pro-apoptotic factors into the cytosol^32^ results in cytochrome c-dependent $\left( {Cytc} \right)$ activation of apoptotic mediator caspase-9^33^. Activated caspase-9, in turn, activates procaspase-3 $\left( {Casp3} \right)$ into caspase-3 $\left( {Casp3^{*} } \right)$^30^. Activated caspase-3 eventually induces apoptotic mediators $\left( {Apop} \right)$^31^.

#### ER stress-induced apoptosis

145 $$\frac{{d\left( {\left[ {Calpain} \right]} \right)}}{dt} = - k_{1}^{ + } \left[ {Ca_{i} } \right]\left[ {Calpain} \right] + k_{1}^{ - } \left[ {Ca_{i} .Calpain} \right],$$

146 $$\frac{{d\left( {\left[ {Ca_{i} .Calpain} \right]} \right)}}{dt} = k_{1}^{ + } \left[ {Ca_{i} } \right]\left[ {Calpain} \right] - \left( {k_{1}^{ - } + k_{2}^{ + } } \right)\left[ {Ca_{i} .Calpain} \right],$$

147 $$\frac{{d\left( {\left[ {Calpain^{*} } \right]} \right)}}{dt} = k_{2}^{ + } \left[ {Ca_{i} } \right]\left[ {Calpain} \right] - k_{3}^{ + } \left[ {Calpain^{*} } \right]\left[ {Casp12} \right] + k_{3}^{ - } \left[ {Calpain^{*} .Casp12} \right],$$

148 $$\frac{{d\left( {\left[ {Casp12} \right]} \right)}}{dt} = - k_{3}^{ + } \left[ {Calpain^{*} } \right]\left[ {Casp12} \right] + k_{3}^{ - } \left[ {Calpain^{*} .Casp12} \right],$$

149 $$\frac{{d\left( {\left[ {Calpain^{*} .Casp12} \right]} \right)}}{dt} = k_{3}^{ + } \left[ {Calpain^{*} } \right]\left[ {Casp12} \right] - \left( {k_{3}^{ - } + k_{4}^{ + } } \right)\left[ {Calpain^{*} .Casp12} \right],$$

150 $$\frac{{d\left( {\left[ {Casp12^{*} } \right]} \right)}}{dt} = k_{4}^{ + } \left[ {Calpain^{*} .Casp12} \right] - k_{5}^{ + } \left[ {Casp12^{*} } \right]\left[ {Casp9} \right] + k_{5}^{ - } \left[ {Casp12^{*} .Casp9} \right].$$

#### MT-induced apoptosis

151 $$\frac{{d\left( {\left[ {ROS_{mit} } \right]} \right)}}{dt} = k_{13}^{ + } \left[ {Stress} \right]\left[ {Mit} \right],$$

152 $$\frac{{d\left( {\left[ {PTP_{mit}^{*} } \right]} \right)}}{dt} = k_{14}^{ + } \left[ {ROS_{mit} } \right]\left[ {PTP_{mit} } \right],$$

153 $$\frac{{d\left( {\left[ {Cytc_{mit} } \right]} \right)}}{dt} = - k_{15}^{ + } \left[ {PTP_{mit}^{*} } \right]\left[ {Cytc_{mit} } \right],$$

154 $$\frac{{d\left( {\left[ {Cytc} \right]} \right)}}{dt} = - k_{16}^{ + } \left[ {Cytc} \right]\left[ {Casp9} \right] + k_{16}^{ - } \left[ {Cytc.Casp9} \right] + k_{15}^{ + } \left[ {PTP_{mit}^{*} } \right]\left[ {Cytc_{mit} } \right],$$

#### Common pathways for both apoptotic signaling pathways

155 $$\frac{{d\left( {\left[ {Cytc.Casp9} \right]} \right)}}{dt} = k_{16}^{ + } \left[ {Cytc} \right]\left[ {Casp9} \right] - k_{16}^{ - } \left[ {Cytc.Casp9} \right] - k_{10}^{ + } \left[ {Cytc.Casp9} \right],$$

156 $$\frac{{d\left( {\left[ {Casp9} \right]} \right)}}{dt} = - k_{5}^{ + } \left[ {Casp12^{*} } \right]\left[ {Casp9} \right] + k_{5}^{ - } \left[ {Casp12^{*} .Casp9} \right],$$

157 $$\frac{{d\left( {\left[ {Casp12^{*} .Casp9} \right]} \right)}}{dt} = k_{5}^{ + } \left[ {Casp12^{*} } \right]\left[ {Casp9} \right] - \left( {k_{5}^{ - } + k_{6}^{ + } } \right)\left[ {Casp12^{*} .Casp9} \right],$$

158 $$\frac{{d\left( {\left[ {Casp9^{*} } \right]} \right)}}{dt} = k_{6}^{ + } \left[ {Casp12^{*} .Casp9} \right] - k_{7}^{ + } \left[ {Casp9^{*} } \right]\left[ {Casp3} \right] + k_{7}^{ - } \left[ {Casp9^{*} .Casp3} \right] + k_{10}^{ + } \left[ {Cytc.Casp9} \right] - k_{11}^{ + } \left[ {Casp9^{*} } \right]\left[ {IAP} \right] + k_{11}^{ - } \left[ {Casp9^{*} .IAP} \right],$$

159 $$\frac{{d\left( {\left[ {Casp3} \right]} \right)}}{dt} = - k_{7}^{ + } \left[ {Casp9^{*} } \right]\left[ {Casp3} \right] + k_{7}^{ - } \left[ {Casp9^{*} .Casp3} \right],$$

160 $$\frac{{d\left( {\left[ {Casp9^{*} .Casp3} \right]} \right)}}{dt} = k_{7}^{ + } \left[ {Casp9^{*} } \right]\left[ {Casp3} \right] - \left( {k_{7}^{ - } + k_{8}^{ + } } \right)\left[ {Casp9^{*} .Casp3} \right],$$

161 $$\frac{{d\left( {\left[ {Casp3^{*} } \right]} \right)}}{dt} = k_{8}^{ + } \left[ {Casp9^{*} .Casp3} \right] - k_{9}^{ + } \left[ {Casp9^{*} } \right]\left[ {Casp3^{*} } \right] - k_{12}^{ + } \left[ {Casp3^{*} } \right]\left[ {IAP} \right] + k_{12}^{ - } \left[ {Casp3^{*} .IAP} \right],$$

162 $$\frac{{d\left( {\left[ {Apop} \right]} \right)}}{dt} = k_{9}^{ + } \left[ {Casp9^{*} } \right]\left[ {Casp3^{*} } \right].$$

#### Inhibitor of apoptosis (IAP) proteins

163 $$\frac{{d\left( {\left[ {IAP} \right]} \right)}}{dt} = - k_{11}^{ + } \left[ {Casp9^{*} } \right]\left[ {IAP} \right] + k_{11}^{ - } \left[ {Casp9^{*} .IAP} \right] - k_{12}^{ + } \left[ {Casp3^{*} } \right]\left[ {IAP} \right] + k_{12}^{ - } \left[ {Casp3^{*} .IAP} \right],$$

164 $$\frac{{d\left( {\left[ {Casp9^{*} .IAP} \right]} \right)}}{dt} = k_{11}^{ + } \left[ {Casp9^{*} } \right]\left[ {IAP} \right] - k_{11}^{ - } \left[ {Casp9^{*} .IAP} \right],$$

165 $$\frac{{d\left( {\left[ {Casp3^{*} .IAP} \right]} \right)}}{dt} = k_{12}^{ + } \left[ {Casp3^{*} } \right]\left[ {IAP} \right] - k_{12}^{ - } \left[ {Casp3^{*} .IAP} \right].$$

### Energy consumption

The approximate ATP consumption in the propagation of action potential and recovery of membrane potential $\left( {uATP_{ep} } \right)$ is given by,

166 $$\frac{{d\left( {uATP_{ep} } \right)}}{dt} = \lambda_{ep} \times \left( {I_{NaK} + I_{pmca} } \right),$$

167 $$\lambda_{ep} = \frac{1}{{F \times vol_{cyt} }}.$$

where, $\lambda_{ep}$ is the scaling factor for electrical processes, $I_{NaK}$ is the sodium–potassium pump current, $I_{pmca}$ is the calcium pump current, $F$ is the Faraday’s constant, and $vol_{cyt}$ is the cytosolic volume.

The approximate ATP consumption in synaptic recycling and neurotransmitter packing into vesicles $\left( {uATP_{sp} } \right)$ is given by,

168 $$\frac{{d\left( {uATP_{sp} } \right)}}{dt} = \left( {\lambda_{sr} \times J_{rel} } \right) + \left( {\lambda_{np} \times J_{VMAT} } \right),$$

where, $\lambda_{sr}$ is the scaling factor for synaptic recycling, $\lambda_{np}$ is the scaling factor for neurotransmitter packing, $J_{rel}$ is the DA release flux from the terminal, and $J_{VMAT}$ is the DA packing flux into the vesicles.

The approximate ATP consumption in calcium influx into the endoplasmic reticulum $\left( {uATP_{er} } \right)$ is given by,

169 $$\frac{{d\left( {uATP_{er} } \right)}}{dt} = \lambda_{er} \times \left( {J_{serca,er} } \right),$$

170 $$\lambda_{er} = \frac{{\beta_{er} }}{{\rho_{er} }},$$

where, $\lambda_{er}$ is the scaling factor for endoplasmic reticulum processes, $J_{serca,er}$ is the calcium influx into ER through SERCA, $\beta_{er}$ is the ratio of free calcium to total calcium concentration in the ER, and $\rho_{er}$ is the volume ratio between the ER and cytosol.

The approximate ATP consumption in damaged protein disposal mechanisms $\left( {uATP_{dm} } \right)$ is given by,

171 $$\frac{{d\left( {uATP_{dm} } \right)}}{dt} = \left( {\lambda_{prt} \times J_{prt} } \right) + \left( {\lambda_{tag} \times J_{tag} } \right) + \left( {\lambda_{lyso} \times J_{lyso} } \right),$$

where, $\lambda_{prt}$ is the scaling factor for proteasomal degradation of damaged protein, $\lambda_{tag}$ is the scaling factor for ubiquitination of damaged protein, $\lambda_{lyso}$ is the scaling factor for lysosomal degradation of damaged protein, $J_{prt}$ is the flux of ATP-dependent breakdown of damaged protein through proteasomal degradation, $J_{tag}$ is the flux of ATP-dependent ubiquitination of damaged protein for proteasomal degradation, and $J_{lyso}$ is the flux of ATP-dependent breakdown of aggregated protein through lysosomal degradation. All the initial values of the differential equations were taken as zero. All parametric and steady-state values are given in Supplementary Table 3.

## Results

**Figure 2:**
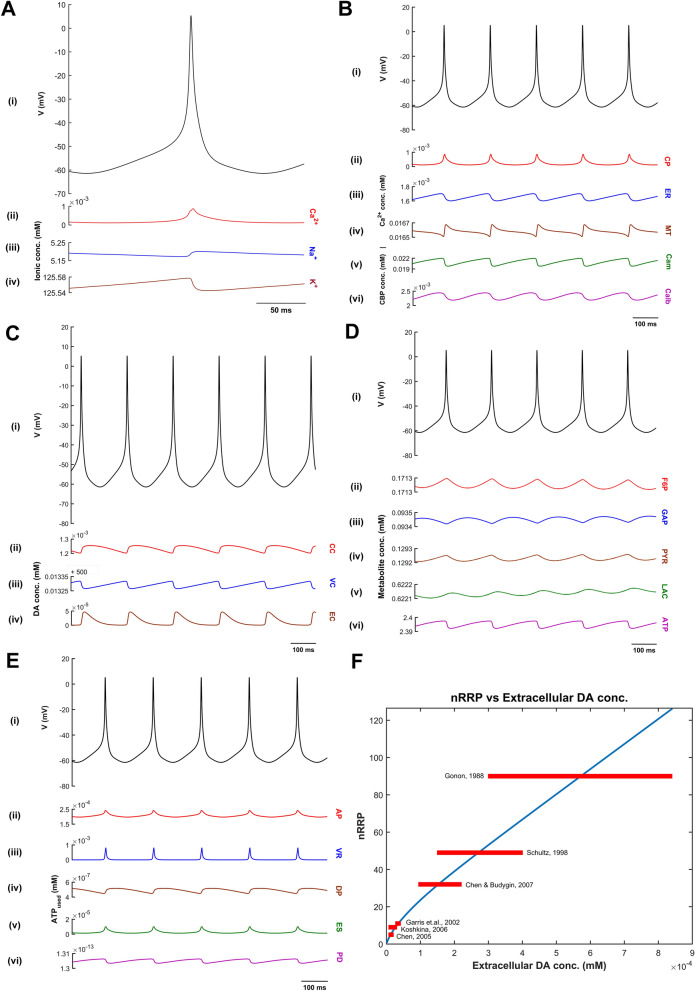
Oscillations in intracellular molecular concentrations in relation to the oscillations of the membrane potential. (**A**) Oscillations in the membrane potential (V) and the corresponding variations of intracellular sodium (Na^+^), potassium (K^+^) and calcium (Ca^2+^) concentrations, (**B**) Oscillations in cytoplasmic (CP), endoplasmic reticulum (ER) and mitochondrial (MT) calcium concentrations and calcium-binding proteins (CBP—Cam & Calb) concentration in relation to the variation of the membrane potential, (**C**) Oscillations in cytoplasmic (CC), vesicular (VC) and extracellular (EC) dopamine (DA) concentrations in relation to the membrane potential, (**D**) Oscillations in fructose-6-phosphate (F6P), glyceraldehyde-3-phosphate (GAP), pyruvate (PYR), lactate (LAC) and adenosine triphosphate (ATP) concentrations in relation to the membrane potential, (**E**) Energy consumption by different cellular processes in the SNc cell, (**F**) Range bar plot of extracellular DA concentration with respect to nRRP. *Cam* Calmodulin, *Calb* calbindin, *conc* concentration, *mM* millimolar, *mV* millivolt, *ATP* adenosine triphosphate, *AP* action potential propagation, *VR* vesicle recycling, *DP *DA packing, *DA* dopamine, *ES* endoplasmic reticulum calcium sequestering, *PD* protein degradation, *nRRP* number of readily releasable vesicle pool.

We developed a comprehensive model of SNc neuron, which exhibits characteristic ionic dynamics (Fig. 2A), calcium dynamics (Fig. 2B), DA dynamics (Fig. 2C), and energy metabolite dynamics (Fig. 2D). The model also exhibits energy consumption by different cellular processes (Fig. 2E) and varying DA released extracellularly based on nRRP (Fig. 2F).

**Figure 3:**
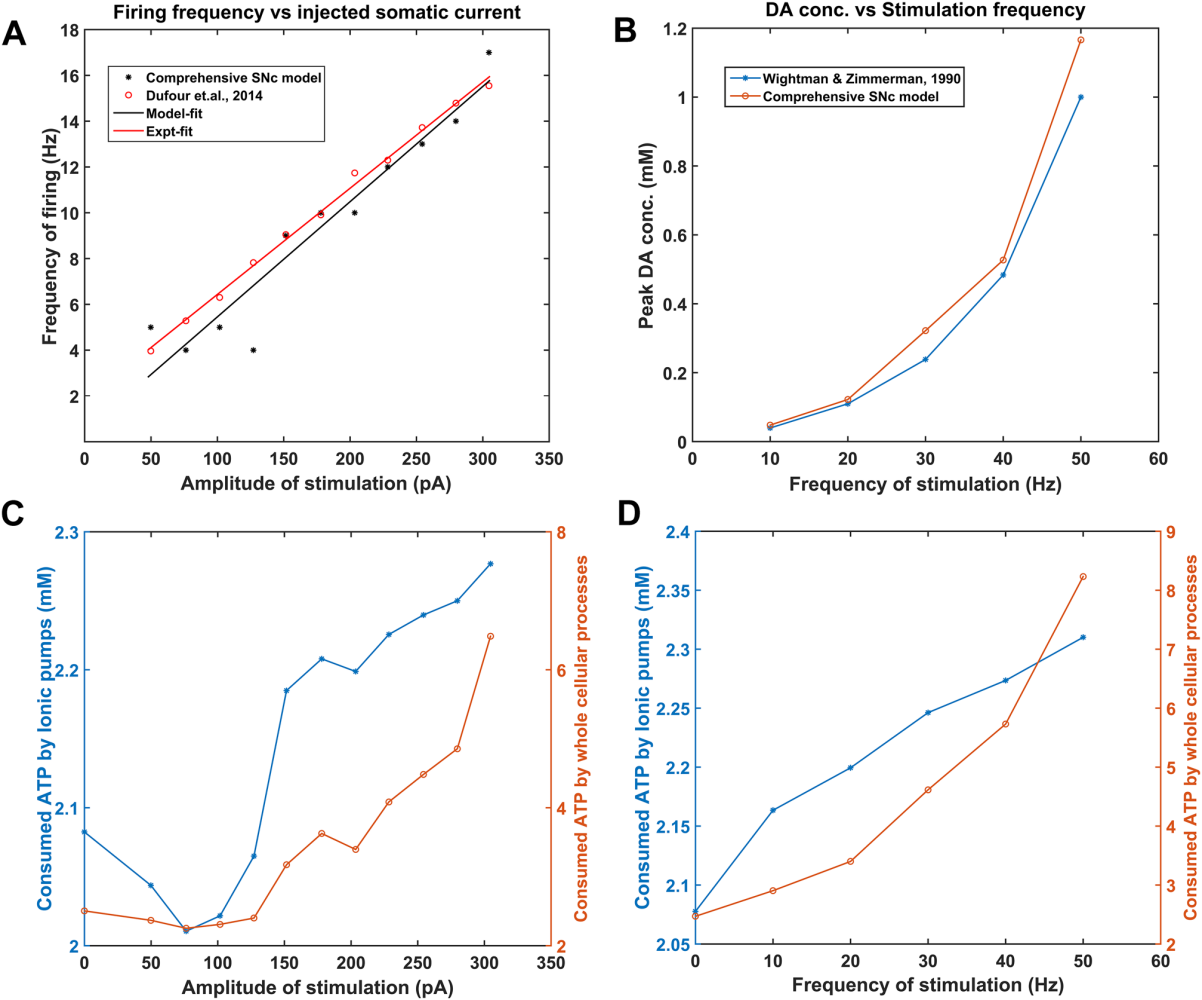
Model response to electrical stimulation. Frequency of firing (**A**) and Energy consumption (**C**) by ionic pumps (blue trace) and all other (whole) cellular processes (orange trace) of the model concerning the amplitude of stimulating depolarized current (1 s), Extracellular dopamine concentration (**B**) and Energy consumption (**D**) by ionic pumps (blue trace) and all other (whole) cellular processes (orange trace) of the model concerning the frequency of stimulating depolarized current (2 secs). *ATP* adenosine triphosphate, *Hz* Hertz, *pA* picoampere, *mM* millimolar.

**Figure 4:**
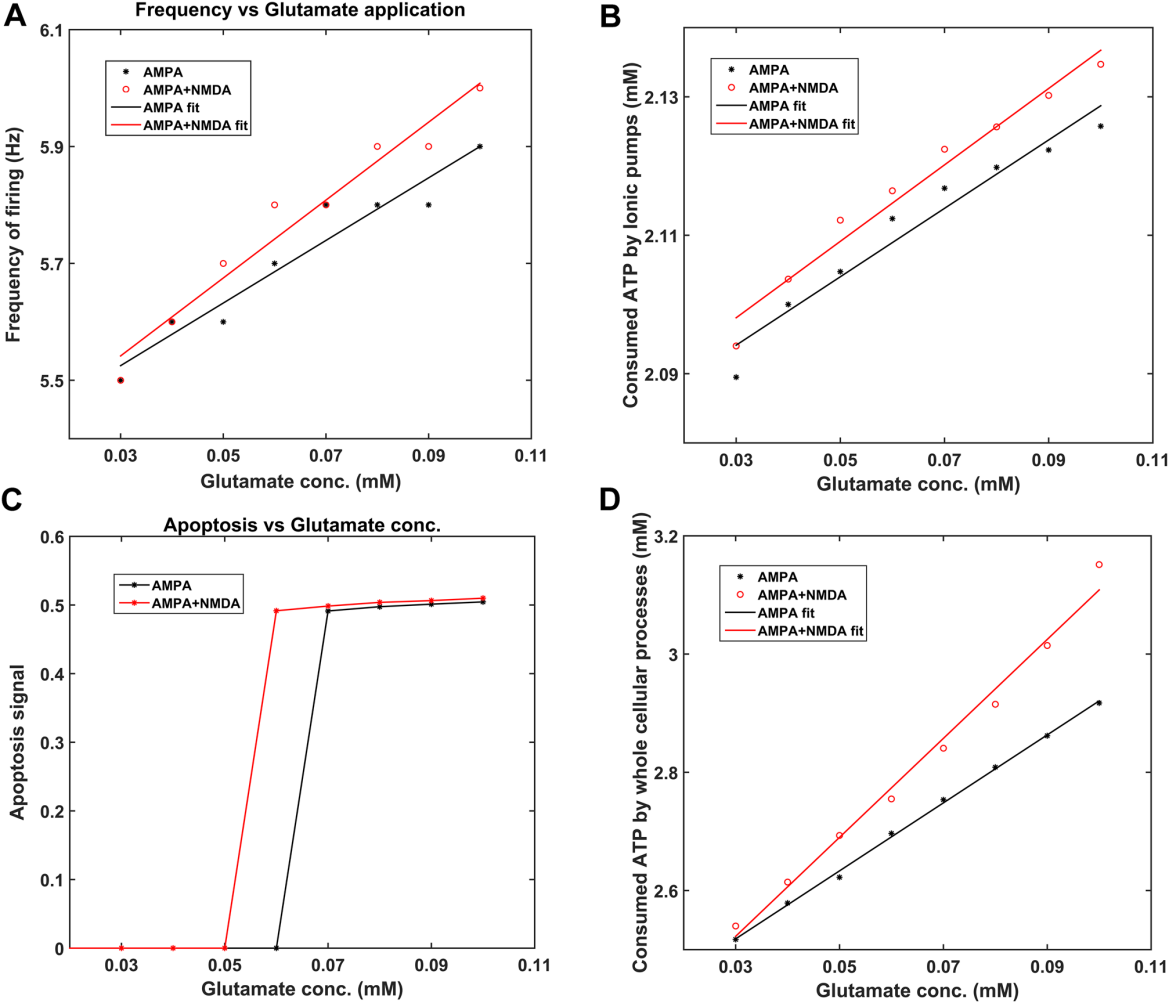
Model response to chemical stimulation (glutamate). Frequency of firing (**A**), Apoptosis signal (**C**) due to excess stimulation, Energy consumption by ionic pumps (**B**) and all other (whole) cellular processes (**D**) of the model concerning the concentration of glutamate application (1 s). *ATP* Adenosine triphosphate, *AMPA* alpha-amino-3-hydroxy-5-Methyl-4-isoxazole propionic acid, *NMDA* *N*-methyl-d-aspartic acid, *Hz* Hertz, *mM* millimolar.

**Figure 5:**
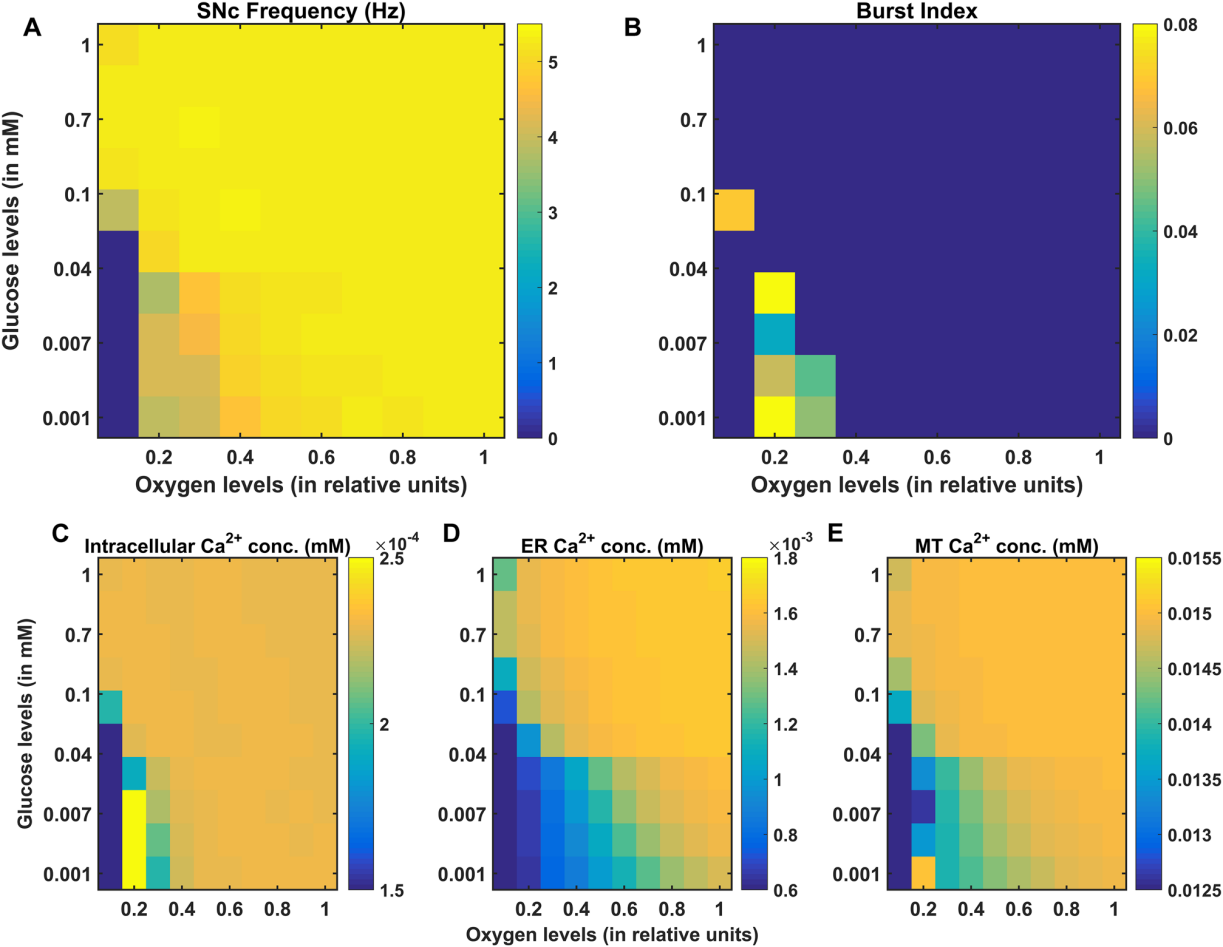
Model response to hypoglycemia and hypoxia conditions. Average frequency of firing (**A**), Bursting (**B**), average intracellular calcium (Ca^2+^) concentration (**C**), average endoplasmic reticulum (ER) calcium concentration (**D**), and average mitochondrial (MT) calcium concentration (**E**) of the model for varying glucose and oxygen concentrations. *SNc* Substantia Nigra pars compacta, *conc* concentration, *mM* millimolar, *Hz* Hertz.

**Figure 6:**
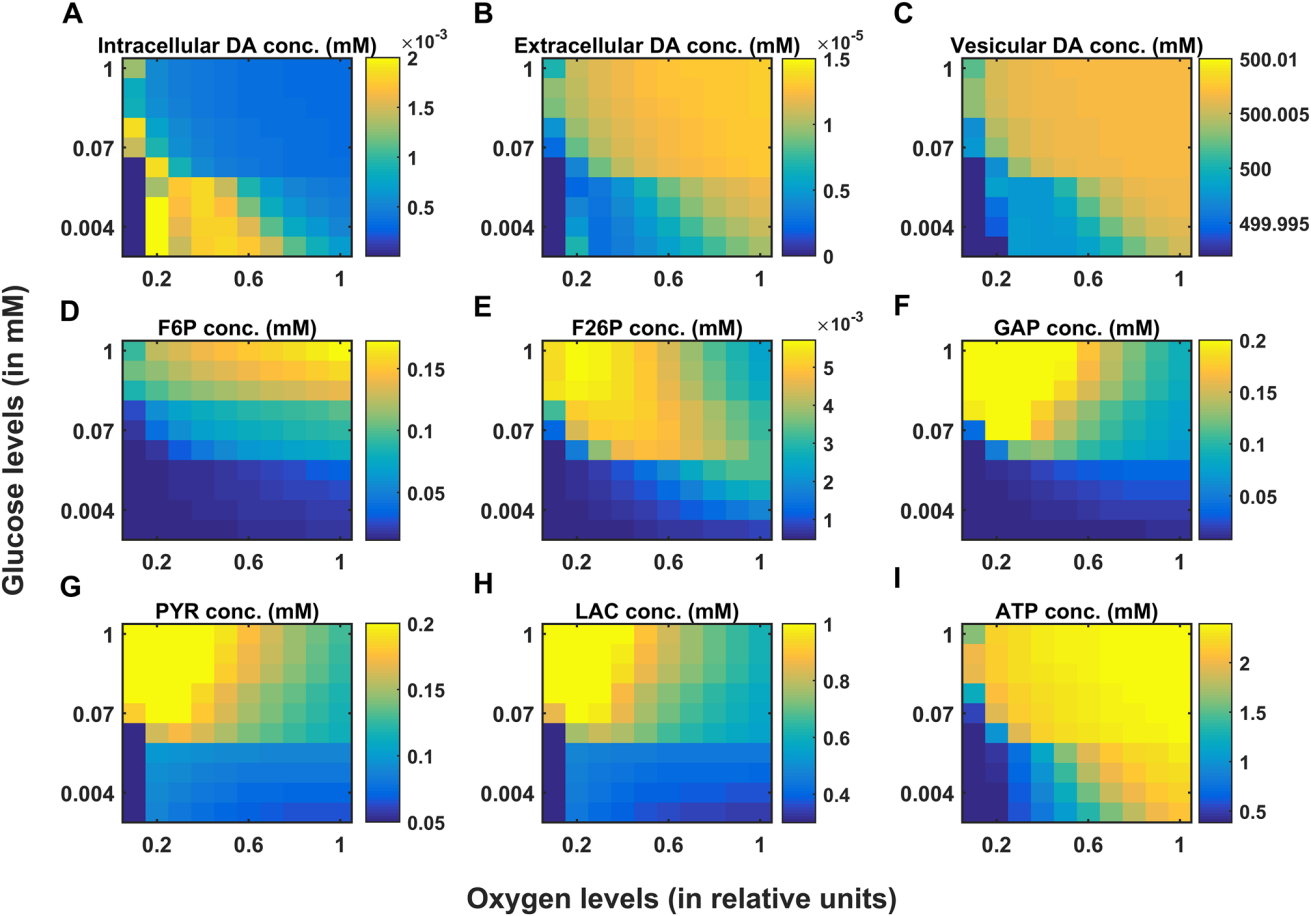
Model response to hypoglycemia and hypoxia conditions. Average intracellular dopamine (DA) concentration (**A**), average extracellular DA concentration (**B**), average vesicular DA concentration (**C**), average fructose-6-phosphate (F6P) concentration (**D**), average fructose-2,6-biphosphate (F26P) concentration (E), average glyceraldehyde-3-phosphate (GAP) concentration (**F**), average pyruvate (PYR) concentration (**G**), average lactate (LAC) concentration (**H**), average adenosine triphosphate (ATP) concentration (**I**) of the model for varying glucose and oxygen concentrations. conc, concentration; mM, millimolar.

**Figure 7:**
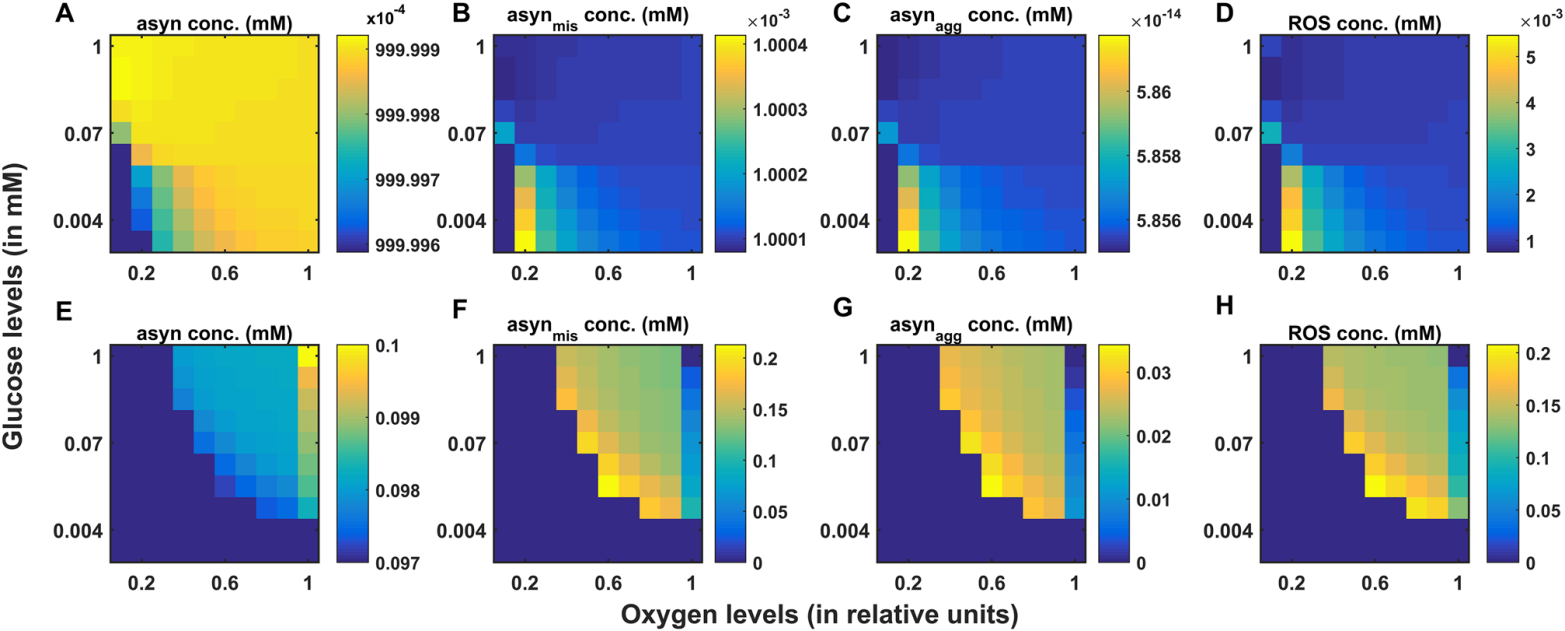
Responses of whole (**A–D**) and reduced (**E–H**) models to hypoglycemia and hypoxia conditions. Average normal alpha-synuclein (asyn) concentration (**A**,**E**), average misfolded alpha-synuclein (asyn_mis_) concentration (**B**,**F**), average aggregated alpha-synuclein (asyn_agg_) concentration (**C**,**G**), and average reactive oxygen species (ROS) concentration (**D**,**H**) of the fast and slow dynamic models for varying glucose and oxygen concentrations. *conc* concentration, *mM* millimolar.

Then, we studied the effect of electrical (Fig. 3) and chemical (Fig. 4) stimulation on the proposed model. Finally, we showed model responses to energy deficiency conditions (Figs. 5, 6, 7).

### Characteristic ionic dynamics of the SNc neuron

The proposed comprehensive model of SNc exhibits the basal firing rate of $5 \,{\text{Hz}}$, which is in the range of $3 \,{\text{to}} \,8 {\text{Hz}}$ observed experimentally^19^ (Fig. 2). The bursting type of firing also observed in the proposed model with a different range of synaptic inputs^19^ (not shown here). The ionic flux concentrations, which drive membrane potential, were in the range of values used in previous models^13,34^. The intracellular calcium concentration during resting state was $\sim 1 \times 10^{ - 4} \,{\text{ mM}}$, which can rise to values greater than $1 \times 10^{ - 3} \,{\text{mM}}$ upon arrival of the action potential^35–37^ (Fig. 2B(ii)). The calcium concentration in the ER was ~ 1000 times higher than in the cytoplasm^35^ (Fig. 2B(iii)). In general, the calcium concentration in the MT will be lesser than the cytoplasm, but due to the higher mitochondrial density^4^ and higher calcium loading in the SNc cells^38,39^, the SNc mitochondrial calcium concentration was much higher than other cells (Fig. 2B(iv)). Accompanying slow calcium buffering mechanisms, calcium-binding proteins such as calbindin and calmodulin act as rapid calcium buffering mechanisms^40^ (mobile calcium buffers), which are present near calcium hotspots and bind rapidly to excess cytoplasmic calcium (Fig. 2B(v, vi)).

### Characteristic dopamine dynamics of the SNc neuron

The link between membrane potential, which was driven by the exchange of ionic concentrations, and extracellular release of DA, which was driven by that membrane potential was described in Tello-Bravo model of DA neuron^9^ (Fig. 2C). The extracellular DA was $\sim 45 \times 10^{ - 6} \,{\text{ mM}}$ which was in the range of $\left( {34 - 48} \right) \times 10^{ - 6} \,{\text{mM}}$ ^41^ (Fig. 2C(iv)) for a number of vesicles in the readily releasable pool $\left( {nRRP = 10} \right)$. The amount of extracellular DA concentration after the quantal release was dependent on the nRRP parameter (Fig. 2F). The cytoplasmic DA concentration was $\sim 12 \times 10^{ - 4} \,{\text{ mM}}$ which was in the range of $10^{ - 4} \,{\text{to}} \,10^{ - 3} \, {\text{mM}}$ ^42^ (Fig. 2C(ii)). The vesicular DA concentration was $\sim 500 \,{\text{mM}}$ which was $10^{3} - 10^{5}$ greater than cytoplasmic DA concentration^36^.

### Characteristic energy metabolite dynamics of the SNc neuron

Active pumps and exchangers maintained the ionic equilibrium across the cell membrane, where ATP drives the sodium–potassium and calcium pumps. Utilizing glucose and oxygen, ATP was produced in the cell through stages of processes such as glycolysis and oxidative phosphorylation (Fig. 2D). The average basal ATP concentration in the SNc cell was $\sim 2.4 \,{\text{mM}},$ which was in the range of $2 - 4 \,{\text{mM}}$^43^ (Fig. 2D(vi)). Other intermediate metabolites in the energy metabolism were in the range similar to Cloutier et al. models^10,17^ (Fig. 2D).

### Energy consumption by different cellular processes of the SNc neuron

The energy consumption in the SNc neuron by different cellular processes, namely action potential propagation, vesicle recycling, DA packing, ER calcium sequestration, and protein degradation was estimated using the proposed model (Fig. 2E). The peak instantaneous ATP consumption for action potential propagation and synaptic transmission (vesicle recycling and DA packing) were $\sim 2.42 \times 10^{ - 4} \,{\text{mM}}$ and $\sim 8.16 \times 10^{ - 3} \,{\text{ mM}}$. The ratio of ATP consumption for action potential propagation to the synaptic transmission was $1:3$ which was similar to Sengupta et al.^44^.

### Model responses to electrical stimulation

In order to study the effect of increased electrical stimulation on firing frequency and DA release, electrical stimulation was carried on the proposed SNc neuronal model. Upon electrical stimulation (pulse width $= 10 \,{\text{ms}}$, frequency $= 20 \,{\text{Hz}}$ and duration $= 1 \,{\text{s}}$) with varying amplitude of stimulation from $50\, {\text{pA}}$ to $300 \,{\text{pA}}$ with similar step size to Dufour et al.^45^, there was not much change in the firing frequency till $130 \,{\text{pA}}$ but increased linearly with increasing stimulation amplitude from $150 \,{\text{pA}}$ onwards (Fig. 3A). Upon electrical stimulation, there was a sharp increase in consumed ATP by ionic pumps at $150 \,{\text{pA}}$ (Fig. 3C, blue trace) clearly correlating with increased firing frequency (Fig. 3A). There was not much change in the consumed ATP by all other cellular processes till $130 \,{\text{pA}}$ but starts to increase with the increase in stimulation amplitude from $150 \,{\text{pA}}$ onwards (Fig. 3C, orange trace) correlating with increased firing frequency (Fig. 3A).

Upon electrical stimulation (pulse width $= 10 \,{\text{ms}}$, amplitude $= 144 \,{\text{pA}}$ and duration $= 2\, {\text{s}}$) with varying frequency of stimulation from $10 \,{\text{Hz}}$ to $50 \,{\text{Hz}}$ with similar step size to Wightman and Zimmerman^46^, there was an increase in peak DA concentration with increased frequency of stimulation (Fig. 3B, orange trace) similar to Wightman and Zimmerman^46^ (Fig. 3B, blue trace). The consumed ATP by ionic pumps, and all other cellular processes increased with increased frequency of stimulation (Fig. 3D).

### Model responses to chemical stimulation

In order to study the effect of glutamate application on the different properties such as firing frequency, energy consumption, and apoptotic signal, chemical stimulation was carried on the proposed SNc neuronal model. Upon chemical application (duration of stimulation $\left( {1 \,{\text{s}}} \right)$) with varying glutamate concentration from $0.03 \,{\text{mM}}$ to $0.1 \,{\text{mM}}$, there was a greater increase in the firing frequency in the presence of both alpha-amino-3-hydroxy-5-Methyl-4-isoxazole propionic acid (AMPA) and *N*-Methyl-d-aspartic acid (NMDA) receptors than AMPA receptor alone (Fig. 4A). A similar trend was observed in the ATP consumption by ionic pumps and all other cellular processes, it was higher for both AMPA and NMDA receptors than AMPA receptor alone (Fig. 4B,D). The apoptosis occurs at lower concentration of glutamate in the SNc neurons with both AMPA and NMDA receptors as opposed to neurons with AMPA receptors alone^39,47^ (Fig. 4C).

### Hypoglycemia and hypoxia conditions

By introducing energy deficiency in the form of hypoxia and hypoglycemia, we now studied the effect of hypoglycemia and hypoxia on the various critical molecular players in the SNc neuron. The energy deficiency conditions were implemented by varying glucose and oxygen levels in the proposed comprehensive model of SNc. The firing frequency of the model decreases (Fig. 5A), and the firing pattern changes from spiking to bursting (Fig. 5B) under severe hypoglycemia (low glucose) and hypoxia (low oxygen) conditions. The average cytoplasmic calcium concentration was higher, which might be due to the reduced outward flux of calcium by active calcium pump and sodium-calcium exchangers as a result of lesser ATP availability at higher extent of hypoglycemia and hypoxia conditions (Fig. 5C). The average ER and mitochondrial calcium concentrations were low, which might be due to reduced sequestration of calcium into ER and MT, which in turn happens due to lesser ATP availability under more severe hypoglycemia and hypoxia conditions (Fig. 5D,E).

The average cytoplasmic DA concentration was higher, which might be due to reduced DA packing into the vesicles as a result of lesser ATP availability under more severe hypoglycemia and hypoxia conditions (Fig. 6A). The average extracellular and vesicular DA concentrations were low, which might be due to reduced readily releasable vesicle pool as a result of lesser ATP availability, which might affect the DA packing into the vesicles under more severe hypoglycemia and hypoxia conditions (Fig. 6B,C).

The average F6P concentration was more affected by reduced glucose than reduced oxygen, and F6P concentration becomes very low for glucose concentration reduced beyond $4 \times 10^{ - 2} \,{\text{mM}}$ (Fig. 6D). The average F26P accumulation was higher during high glucose and low oxygen, which was an integrator of metabolic stress^17^ (Fig. 6E). The average GAP, average PYR, and average LAC concentrations were higher during high glucose and low oxygen due to GAP and PYR being the intermediate metabolites in the glycolytic pathway and LAC being the by-product of anaerobic respiration (in the absence of oxygen) (Fig. 6F,G,H). The average ATP concentration under normal condition was $\sim 2.4 \,{\text{mM}}$ which was in the range of $2 - 4\,{\text{ mM}}$^43^, and ATP concentration becomes significantly low for glucose concentration reduced beyond $4 \times 10^{ - 2} \,{\text{mM}}$ (Fig. 6I). At low glucose and low oxygen, ATP level reaches a point where SNc neuron adapts and starts bursting (Fig. 5A) to transmit maximum information with minimal usage of energy^48,49^ (Fig. 6I). At low glucose $( < 5 \times 10^{ - 2} \,{\text{mM}})$ and very low oxygen $( < 0.2)$ (relative units) levels, the SNc neuron undergoes degeneration (Fig. 6).

In the whole (fast dynamics) model simulation, the healthy alpha-synuclein protein (asyn) was misfolded, and the available healthy alpha-synuclein protein was low at low glucose and low oxygen (Fig. 7A,E). Under low glucose and low oxygen conditions, the accumulation of misfolded alpha-synuclein (asyn_mis_) and alpha-synuclein aggregates (asyn_agg_) was higher due to lesser ATP availability, which leads to reduced proteolysis or protein degradation (Fig. 7B,C). The average ROS concentration was increased at low glucose and low oxygen levels due to misfolded alpha-synuclein, thereby inducing further release of ROS by hindering mitochondrial functioning (Fig. 7D). For a better representation of molecular markers under pathological conditions, the reduced (slow dynamics) model was simulated, which was obtained by assuming fast substrates reaching their steady states rapidly, and associated differential equations were transformed into functions (that is, at steady-state values). The average normal alpha-synuclein concentration decreases with a decrease in glucose and oxygen levels due to increased ROS-induced misfolding of alpha-synuclein (Fig. 7E). The deleterious effect of ROS/asyn_mis_ leads to a vicious cycle where the formation of ROS and asyn_mis_ is mutually reinforced^10^, which was evident from simulation results also. The average ROS concentration during normal condition was in the range of $1 \times 10^{ - 3} - 5 \times 10^{ - 3} \,{\text{ mM}}$ and during hypoglycemia and hypoxia conditions it reached beyond the concentrations $\left( {0.01 - 0.015 \,{\text{mM}}} \right)$^50^*,* which was observed in the disease state (Fig. 7H). Due to higher ROS concentration, alpha-synuclein misfolding and aggregation were prominent, and the concentrations are reaching values similar to high-stress conditions^10^ (Fig. 7F,G).

## Discussion

The central objective of this computational study is to show that metabolic deficiency is the root cause that connects various molecular level pathological manifestations of PD in SNc cells. More importantly, we want to investigate the hypothesis that metabolic deficit is perhaps the root cause of SNc cell loss in PD. The proposed model is one of its kind, which explains how deficits in supply of energy substrates (glucose and oxygen) can lead to the pathological molecular changes, including alpha-synuclein aggregation, ROS production, calcium elevation, and DA deficiency. The proposed model is compared to other models, that at least had more than one cellular process modeled together (Table 1).

**Table 1:** Comparison of the proposed model with previously published models.

Model	Ion channels	Calcium buffering	Energy metabolism	Dopamine turnover	Levodopa uptake	ROS/α-syn	Apoptosis
Tello-Bravo^9^	**✓**	**✗**	**✗**	**✓**	**✗**	**✗**	**✗**
Reed et al.^12^	**✗**	**✗**	**✗**	**✓**	**✓**	**✗**	**✗**
Cloutier and Wellstead^10^	**✗**	**✗**	**✓**	**✗**	**✗**	**✓**	**✗**
Francis et al.^13^	**✓**	**✓**	**✗**	**✗**	**✗**	**✗**	**✗**
Cullen and Wong-Lin^11^	*	**✗**	**✗**	**✓**	**✗**	**✗**	**✗**
Proposed model	**✓**	**✓**	**✓**	**✓**	**✓**	**✓**	**✓**

*No ion channel dynamics but has spiking behavior (Izhikevich neuronal model^51^).

### Different regimes with varying energy substrates

**Figure 8:**
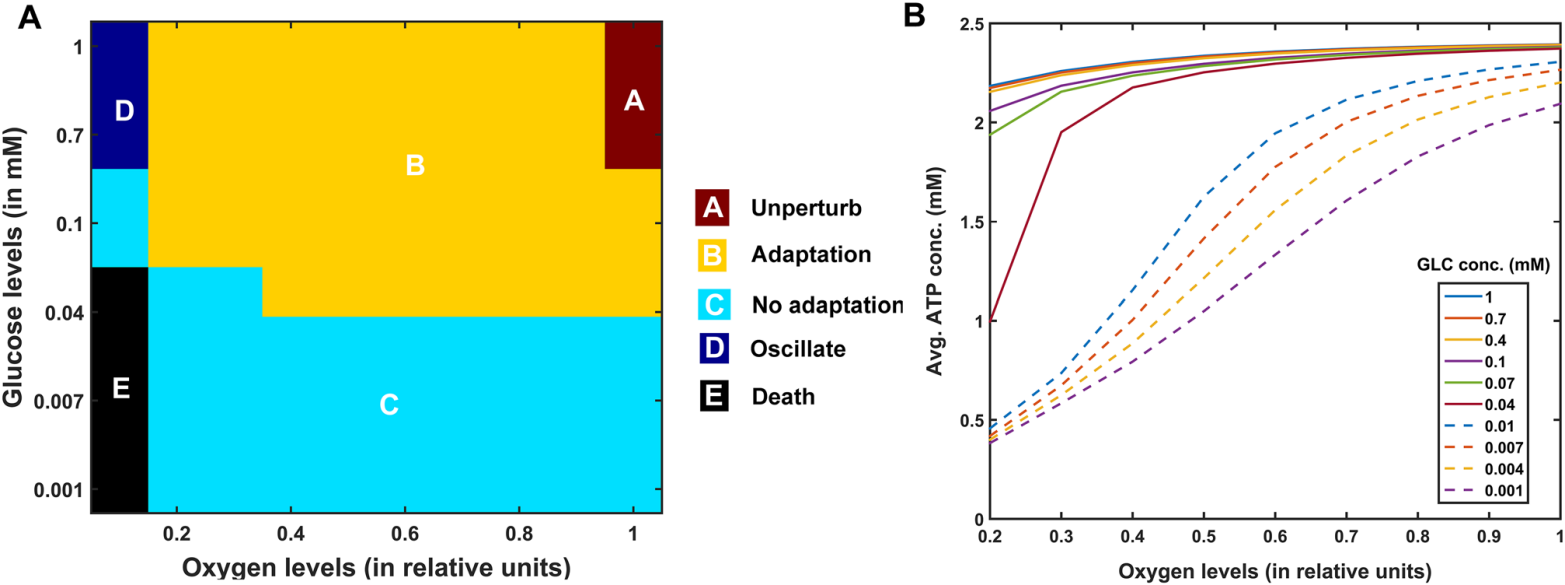
Model responses to hypoglycemia and hypoxia conditions. (**A**) Different regimes of the model response to hypoglycemia and hypoxia conditions, (**B**) average ATP concentration for different initial glucose concentration concerning oxygen concentration. *conc* concentration, *mM* millimolar, *GLC* glucose, *ATP* adenosine triphosphate.

The proposed model with its biophysical framework shows four regimes of ATP dynamics as a function of glucose and oxygen levels: (A) Unperturbed (no change in Basal ATP Concentration (BAC)), (B) adaptation (initial drop and a subsequent return to initial BAC)^52^, (C) no adaptation (initial drop and stabilized at a lower BAC, however, generally astrocytes and other energy sources (glycogen, glutamine) will restore ATP levels^53^), and (D) oscillating (BAC fluctuates, where anaerobic respiration might occur^54^) and other regimes in which neuron undergoes degeneration (Fig. 8A). The basal ATP concentration patterns for different dynamic regimes were shown in Supplementary Fig. 10. The model also suggests that hypoglycemia plays a more crucial role in leading to ATP deficits than hypoxia (Fig. 8B). From the modelling results, the relative levels of ATP consumption in different cellular processes can be described as: synaptic transmission > action potential propagation > endoplasmic reticulum calcium sequestration > protein degradation^55,56^.

In PD, energy deficiency occurs in a targeted fashion over a long period of time, which first affects the most vulnerable neurons and spreads to less vulnerable neurons in the brain. So, when compared to the glutamatergic neurons, SNc neurons are one of the most vulnerable and energy-consuming neuronal clusters, due to their structural and functional properties^57^ such as complex axonal arborization^4,5^, pacemaking ion channels^58^ (auto-rhythmicity), presence of reactive neuromodulator^59^ (dopamine), excitotoxicity^3,7^, calcium loading and higher basal metabolic rates associated with chronically elevated ROS production^4^. Taking out all these plausible factors, SNc cells prone to be the most susceptible to energy deficiency.

### Excitotoxicity precipitated by energy deficiency

During chemical stimulation or synaptic evoked action potential, glutamate concentration varies from $0.03\,{\text{ mM}}$ to $0.1 \,{\text{mM}},$ which was in the range observed in the synaptic cleft $\left( {2 \times 10^{ - 3} - 1 \,{\text{mM}}} \right)$ and the binding affinities of NMDA $\left( {2 \times 10^{ - 3} - 3 \times 10^{ - 3} \,{\text{mM}}} \right)$ and AMPA $\left( {0.4 - 0.5 \,{\text{mM}}} \right)$ receptors^60^. From the proposed model, the SNc neurons with both AMPA and NMDA receptors are more prone to apoptosis than SNc neurons with AMPA receptor alone^39,47^ (Fig. 4C). Thus, the long-term influence of NMDA activation (longer time constant than that of AMPA) in the SNc neuron plays an important role in PD pathogenesis^39,61^. Under energy deficit conditions, SNc neurons undergo apoptosis due to overexcitation with even physiological concentrations of glutamate when compared to normal conditions^52^ (not shown here). We suggest that the excitotoxic loss of SNc neurons in PD might be precipitated by energy deficiency^3^. Any therapeutic interventions that can reduce ionic flux through these glutamatergic receptors or enhance energy production can be neuroprotective in nature^62–64^.

### SNc vulnerability in PD

PD can be caused due to damage to glutaminergic neurons as a result of energy deficiency (which is caused by ischemic stroke). However, PD is a slowly evolving disease unlike sudden ischemic stroke which leads to a sudden drop in energy substrates. In PD, energy deficiency occurs in a targeted fashion over a long period of time which first affects the most vulnerable neurons and spreads to less vulnerable neurons in the brain. So, when compared to the glutamatergic neurons, SNc neurons are one of the most vulnerable and energy consuming neuronal clusters, due to their structural and functional properties. We list out some of the plausible factors which make SNc cells to be most susceptible.

- *Complex axonal arbors* Large axonal arborisation that requires large amounts of energy to drive currents along these axons^5,65^,
- *Reactive neurotransmitter* When a reactive neurotransmitter like DA is present in excess, it would readily oxidize with proteins, nucleic acids and lipids^38^ eventually leading to neurodegeneration. One of the mechanisms for sequestration of excess cytosolic DA is packing of DA into synaptic vesicles through VMAT-2 using H^+^ concentration gradient which is maintained by H^+^-ATPase. In addition, in case of substantia nigra, the expression of VMAT-2 is lower than in the ventral tegmental area (VTA)^59,66^ which likely causes DA-mediated oxidative stress in SNc cells,
- *Auto-rhythmicity* Use of L-type calcium channels for maintaining pace-making type of firing which in turn requires higher amounts of energy to maintain calcium homeostasis^67^ and lower expression of calcium-binding proteins (lower capacity of calcium buffering mechanism) adds additional burden on the SNc cell’s metabolic activity^68^,
- *NMDA synaptic activation* Due to pacemaker type of firing, magnesium blockage of NMDA receptors is ineffective, resulting in substantial NMDA receptor currents even with weak glutamatergic inputs resulting in additional burden to maintain calcium homeostasis; the resulting energy deficiency leads to excitotoxicity^39,69^,
- *Prone to neuroinflammation* Astrocytes play a modulatory role in microglial activation^70–72^ and any miscommunication between them results in neuroinflammation which eventually leads to neurodegeneration^73,74^. The risk of inflammation in SNc neurons is high due to the small proportion of astrocytes regulating the huge population of microglia in this region^75,76^. It has been reported that neuromelanin can induce microglial activation^77,78^. SNc neurons are more susceptible to neuro-melanin induced inflammation compared to VTA neurons due to their high neuro-melanin biosynthesis as a result of underexpression of VMAT-2^66^.
- *Weak microvasculature* SNc neurons are more prone to environmental toxins due to weak surrounding cerebral microvasculature^79^.

Since the metabolic demands of SNc neurons are particularly high when compared to any other neuronal types^38^ including neurons of other dopaminergic systems^4,5,80^, any sustained insufficiency in the supply of energy can result in cellular degeneration, characteristic of PD^81^.

The effect of glutamate released from glutaminergic neurons onto SNc neurons can be considered as toxic, in addition to its regular action of neurotransmission during energy deficit conditions^82,83^. During the pacemaking activity of SNc, the magnesium block of NMDA receptors on SNc neurons becomes ineffective. As a result, a slightly increased glutamate stimulation can create a calcium storm in SNc neurons^39^. This direct mechanism of toxicity is possible in case of acute neurological disorders such as ischemic/hypoxic damage to the brain (which was termed as ‘strong excitotoxicity’) but not in slowly evolving chronic diseases such as PD^39^. However, under energy deficit conditions, even physiological levels of glutamate are toxic as a result of increased intracellular calcium concentration, which leads to oxidative stress through a mechanism known as indirect excitotoxicity or weak excitotoxicity^84^. It was reported that the glutamatergic excitation of SNc neurons by STN neurons^85^ under the conditions of bioenergetic deficiency might lead to aggravation of degeneration processes^3,52,69^.

### Insights into the various phenotypes of PD (determinants at different levels)

In genetics, the phenotype of an organism depends on the underlying genotype^86^. Similarly, the occurrence of different phenotypes of a disease can be driven by underlying dysfunctions occurring at different levels in the hierarchy, such as molecular, cellular, and systems levels^87,88^. In PD, the loss of dopaminergic neurons in SNc results in the manifestation of PD symptoms, and the cause of the SNc cell loss is still not clearly elucidated. The PD phenotypes are distinct, and this specificity might be arising out of a combination of interactions between key determinants at the same or different levels.

**Figure 9:**
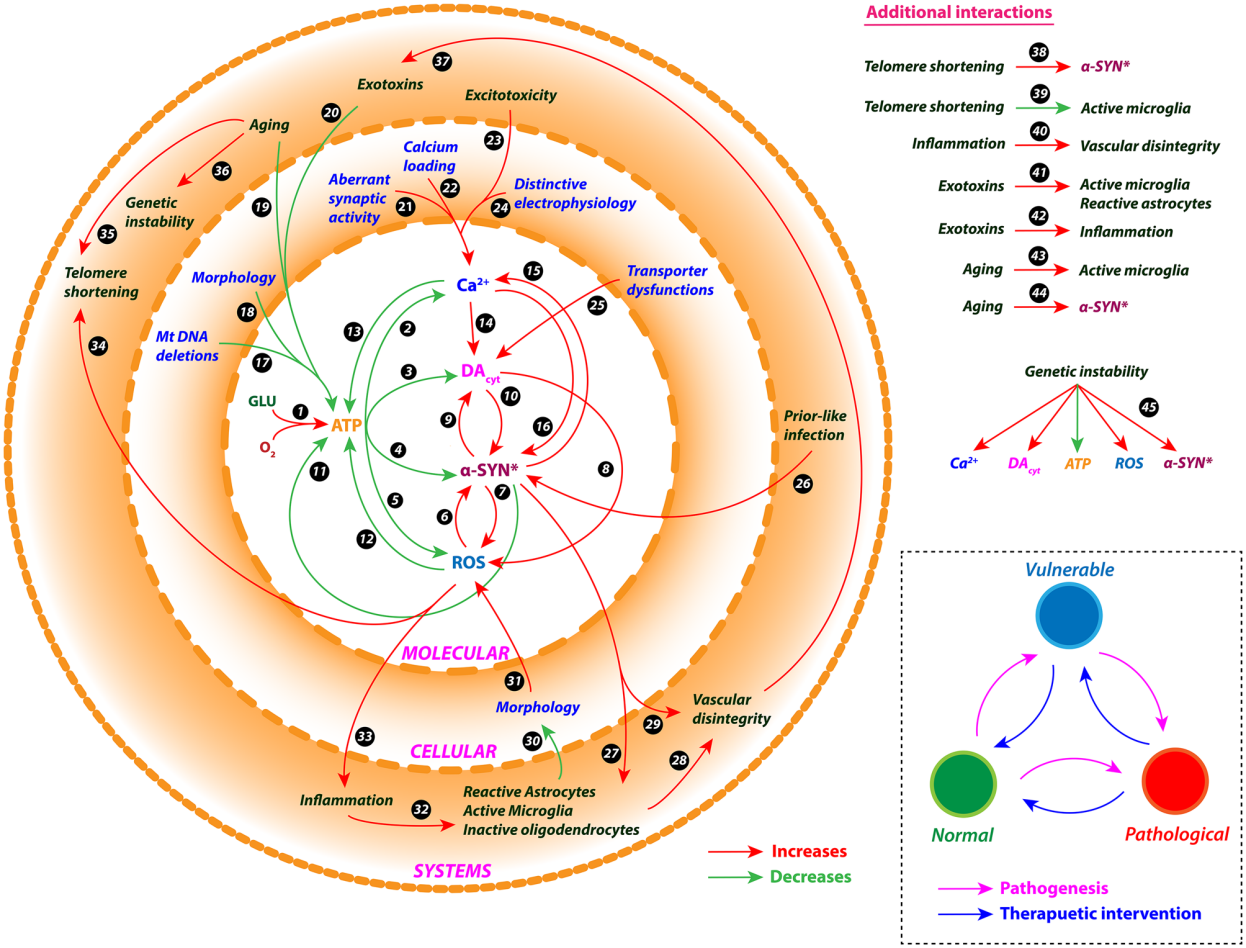
Interactions among the determinants at different levels of hierarchy. See Box-1 for description of the Figure.

At the molecular level, the interactions among divergent key determinants such as ATP, cytoplasmic DA (DA_c_), alpha-synuclein (ASYN), ROS, and cytoplasmic calcium (Ca^2+^) converges to common pathologies or pathways such as oxidative stress, mitochondrial impairment, and protein mishandling^89–91^. The dysfunction causing interactions among different molecular determinants^91–93^ was elaborated in Fig. 9.

At the cellular level, the determinants that might contribute to differential PD phenotypes are complex morphology^4,5,65^ (due to large axonal arborization and numerous synaptic connectivity), lesser mitochondrial mass^94,95^ (due to higher level of mitochondrial DNA deletions), high levels of reactive cytosolic DA^66,96,97^ (due to underexpression of vesicular monoamine transporter 2 and overexpression of DA transporter), distinctive electrophysiology^98–100^ (due to broad spikes and pacemaking activity), calcium loading^97,101,102^ (due to presence of Ca_v_1.3 calcium channels and low calcium buffering) and aberrant excitatory synaptic activity^39,103^ (due to ineffective magnesium blockage of NMDA receptors and increased NMDA receptor subunit NR1). These cellular determinants individually or collectively would result in higher basal metabolic rate and increased oxidative stress^4^, which in turn converges to common pathologies^104^ (Fig. 9).

At the systems level, the determinants that might contribute to differential PD phenotypes are excitotoxicity^3,105^ (due to overexcitation by STN or pedunculopontine nucleus), aging^106,107^ (due to proteostatic dysfunction, mitochondrial dysfunction, genetic mutations or telomere shortening), genetic instability^108–110^ (due to changes in nucleic acid sequences, chromosomal rearrangements or aneuploidy), environmental toxins^111,112^ (due to exposure to insecticides, commercial solvents, metal exposure or traumatic head injury), neuroinflammation^113,114^ (due to traumatic head injury, exotoxins or immune dysfunctions), prion-like infection^114,115^ (bacteria or viruses), telomere shortening^116,117^ (due to aging or oxidative stress), glial dysfunction^118–120^ (due to phagocytic or inflammatory impairments, enteric glial dysfunction) and vascular dysfunction^121,122^ (due to endothelial dysfunction or cardiovascular autonomic dysfunction). These systems-level determinants interact among themselves and also across different levels in the hierarchy resulting in different PD phenotypes (Fig. 9).
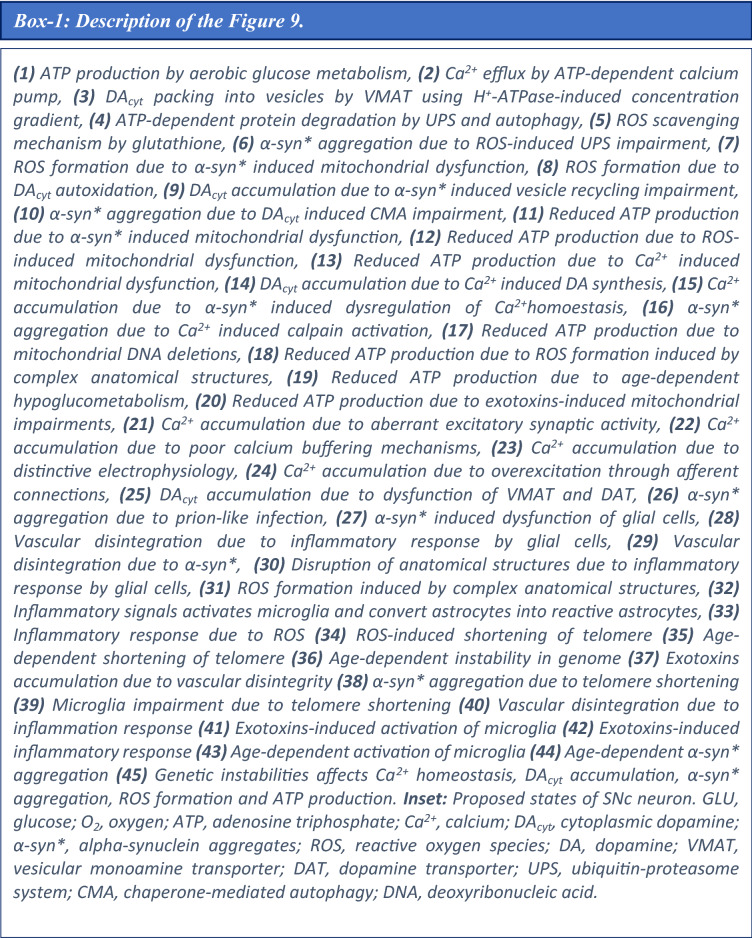


Dysfunctions at any level of hierarchy would make SNc cells move from normal state to pathological state directly or indirectly via an intermediate (vulnerable) state (Fig. 9, inset). Any therapeutics that can bring back SNc neurons from a pathological or vulnerable state to normal state can be beneficiary for the survival of SNc neurons.

### Role of oxidative stress in PD neurodegeneration

In Pavlin et al.^123^, the authors talk about neurodegeneration occurring in two possible pathways at the molecular level^123^. Firstly, the insoluble amyloid plaques prevent the vesicular transport functioning which leads to progressive neurodegeneration. These amyloid plaques are formed as a result of enhanced interaction between alpha-synuclein and oxidized heavy atom ions (increased ROS oxidizes heavy atom ions). Secondly, the dyshomeostasis occurring due to loss of lipid bilayer membrane permeability of the mitochondrial wall or cellular membrane leads loss of electronic gradients in turn resulting in loss of resting potential and neurodegeneration. The membrane permeability is disturbed due to increased interaction between ROS and methylene groups of lipid bilayer. In the present study, the ROS formation is contributed by respiratory chain complexes (Eq. 133), external oxidative stress factors (which includes environmental toxins, extracellular inflammatory responses etc.) and DA autooxidation (Eq. 134). The ROS is scavenged by catalase (Eq. 135) and glutathione (Eq. 104).

Apart from these factors, the ROS formation is contributed by DA metabolized by MAO B enzyme, heavy metal ions and inflammatory responses (late stages of the disease)^123^ also need to be considered. However, it should be mentioned that there are several factors that aggravate or mitigate the effect of ROS, incorporating all these factors will increase the complexity of the model whereas our main focus was to study the effect of energy deficiency on the major molecular players such as calcium, DA, ATP and the membrane voltage (Supplementary Fig. 9). The interaction among the various important players (such as calcium, DA, spike frequency and ATP) was illustrated and along with both positive and negative feedback loops in the Supplementary Fig. 9. In normal conditions, ATP maintains low levels of calcium in the cytoplasm by efflux of excess calcium into the extracellular space and sequestrating the excess calcium into the endoplasmic reticulum. ATP also regulates the vesicular DA levels by maintaining H^+^ concentration gradient which in turn stabilizes the amount of DA released extracellularly. As cytoplasmic calcium increases, the extracellular DA release also increases. However, on continued release of extracellular DA, the cytoplasmic calcium subsequently decreases by the feedback, regulatory action of DA via DA autoreceptors^22^.

The deficiency in the supply of energy substrates results in reduced levels of ATP which in turn affects the homeostasis of cytoplasmic calcium and the amount of extracellular DA released. As ATP decreases, cytoplasmic calcium increases as a result of reduced efflux of calcium from the cytoplasm which in turn maintains the cell in depolarization state (highly excitable) which eventually leads to excitotoxicity (excitotoxicity due to energy deficiency is termed as ‘weak excitotoxicity’^124^). As ATP decreases, extracellular DA released also decreases as a result of reduced packing of DA into the vesicles in turn leading to excess DA build up in cytoplasm which eventually results in oxidative stress.

As we started to develop the proposed model, we looked into several factors that contribute to neurodegeneration and we tried to incorporate the primary factors that affect the neuronal survivability. We agree that the missing factors which contribute towards ROS formation should be incorporated in the future studies which we believe will enhance the scope of the model.

### Role of levodopa in PD neurodegeneration

Levodopa (L-DOPA), a precursor of DA, is used as a symptom-relieving treatment for PD^125^. The usage of L-DOPA for PD is still debated due to its side-effects with long-term treatment^126–128^. Several researchers suggested that L-DOPA might be harmful to SNc cells by a mechanism that probably involves oxidative stress^129–131^. However, several others proposed that L-DOPA might not accentuate neurodegeneration of SNc neurons^127,132,133^ and sometimes acts a neuroprotective agent^127,134,135^ or promote recovery of dopaminergic markers in the striatum^136,137^. After several studies, it is still not clear whether L-DOPA is toxic^128,138–142^.

However, if PD is considered as a metabolic disorder then the mechanism behind L-DOPA-induced toxicity in SNc neurons can be postulated. As the disease progresses, the effect of L-DOPA starts to wear off. Therefore, in order to have the same symptom-relieving effect, the dosage of L-DOPA needs to be increased. When L-DOPA concentration is optimal, L-DOPA might not lead to loss of SNc neurons, and its therapeutic benefits can be maximized. However, when the available concentration of L-DOPA is high, the loss of dopaminergic neurons occurs due to L-DOPA-induced toxicity. This might occur due to higher cytoplasmic DA levels as a result of higher influx of L-DOPA into SNc neurons along with lower vesicular packing of DA (due to energy deficiency) and L-DOPA-induced stimulation of DA metabolism^59^ result in DA-mediated oxidative stress in the SNc neurons^143,144^. Due to higher DA levels and energy deficiency, DA in SNc neurons causes oxidative stress, which leads to SNc neuronal loss. It has been suggest that adjunct therapies such as antioxidants^142,145–148^ and other potential therapies such as D2 agonists^149^, glycogen synthase kinase 3 inhibitors^150^, calcium-binding protein drugs^151^, etc. co-administrated along with L-DOPA might evade LDOPA toxicity in all stages of PD. Thus, the beneficial or toxic effects of L-DOPA needs to be investigated with more thorough experiments performed at preclinical and clinical levels.

### Role of DA transporters on DA availability

Synaptic transmission requires the presynaptic release of neurotransmitter from synaptic vesicles (SVs) onto the postsynaptic neuron. Vesicular neurotransmitter transporter proteins, which use a V-ATPase-generated proton gradient, play a crucial role in packaging neurotransmitter into SVs. The vacuolar H + ‐adenosine triphosphatases (vATPases) acidify multiple intracellular organelles, including SVs and secretory granules. Acidification of SVs represents a critical point during the SV cycle: without acidification, neurotransmitters cannot be loaded into SVs^152,153^. So, the acidic interior of SVs is maintained by ATP in normal conditions. However, during energy deficiency conditions, H + concentration gradient is not maintained which leads to improper packing of DA into SVs result in increased cytoplasmic DA. Excess cytoplasmic DA undergoes non-enzymatic autoxidative reaction (as pH value in the cytoplasm is about 7) giving rise to a superoxide anion that further decomposes to reactive oxygen species result in oxidative stress^154,155^. In the proposed model, vesicular packing of DA is regulated by ATP availability which is described in Eq. (109), where decreased ATP levels leads to decreased vesicular DA levels due to inefficient packing of DA into vesicles. So, the effect of pH on DA availability can be studied indirectly in the proposed model where decreased ATP leads to increase pH in SVs (imbalanced H + concentration gradient) which in turn increases cytoplasmic DA (as a result inefficient packing of DA into SVs) resulting in DA-autooxidation mediated oxidative stress.

Dopaminergic neurons of substantia nigra exhibit broad action potentials (> 2 ms) and two distinct firing patterns: low-frequency irregular tonic or background firing (1–5 Hz)^156^ and high-frequency regular phasic or burst firing (~ 20 Hz)^157^. Dopaminergic neurons are autonomously active and produce a constant background firing pattern on which bursts may be superimposed. The pacemaking type of behavior is necessary to maintain a constant DA level to their innervating regions, such as striatum in case of SNc. Tonic DA levels preferentially activate high affinity D2-type DA receptors, while phasic DA release saturates D2-type receptors and activates low affinity D1-type DA receptors^158^. Tonic and phasic signaling are both required for the execution of motivated behaviors and work together to reinforce advantageous outcomes while reducing disadvantageous behaviors. The amount of autoxidized DA and therewith associated ROS production is proportional to the level of cytoplasmic DA. DA levels in the synaptic gap, cytoplasm and extracellular space increases as a result of VMAT-2 inhibition by amphetamine or reserpine and DAT inhibition by amphetamine or cocaine^159^. Both cocaine and amphetamine acutely elevate tonic DA levels, but result in reduced basal extracellular DA levels as measured by microdialysis 18 h following extended access self-administration, possibly as a compensatory response to chronic drug-induced DA elevations^159^.

One possible mechanism for reductions in basal DA levels following cocaine or amphetamine self-administration is that increased synaptic DA levels, due to drug-induced inhibition of uptake, are subject to enzymatic degradation rather than repacking into vesicles, thus, release may be reduced, and be made more dependent on DA synthesis. In support of this hypothesis, it has been observed that reductions in electrically evoked DA release following extended access cocaine self-administration, suggesting that intracellular DA levels are reduced^160^. In contrast, following extended access amphetamine self-administration, intracellular and extracellular DA levels appear to be differently affected, whereby extracellular levels are decreased and electrically evoked DA release is increased. Both cocaine and amphetamine acutely augment the amplitude and frequency of phasic DA signals which likely results in enhanced phasic DA responses to environmental stimuli when cocaine or amphetamine are on board^160^. Thus, the differential effect of cocaine and amphetamine can be accounted through their effect on DAT only and VMAT-2 and DAT, respectively^159^.

### Effect of MAO on DA availability

MAO is an enzyme found everywhere in the body inside the cells. There are two types of MAOs: MAOs in the intestines are predominantly type A, while most of the MAOs in the brain are type B. In the brain, MAO-B plays an important role in the breakdown of neurotransmitters (chemical messengers) like DA. MAO inhibitors (MAOI) such as selegiline, rasagiline etc. block the action of the enzyme MAO B^161^. Rasagiline is about 10 times more potent in the inhibition of MAO-B than selegiline as demonstrated^162,163^. This higher potency of rasagiline is corrected in the clinic with dose adjustments (approved daily dose 1 and 5–10 mg for rasagiline and selegiline, respectively)^164^. MAO inhibition increases the amount of DA available for release while COMT inhibition does not cause a change in the dynamics of DA, thus MAO plays an important role in DA availability^165^. In the proposed model, DA metabolism by MAO-B in synaptic bouton and extracellular space was simplified where the excess cytoplasmic DA after packing into vesicles is metabolized (see Eq. 121) and excess extracellular DA after reuptake is metabolized (see Eq. 113). In the proposed model, the inhibitory effect of selegiline and rasagiline can be implemented by regulating the kinetic rate constants of Eqs. (113) and (121) where decreasing these rate constants will increase DA levels. However, to understand the potency of rasagiline over selegiline, we should be formulating the metabolism of DA in greater detail which can be incorporated in the future studies so the differential effect of MAO inhibitors can be elucidated.

### Improvements from previous dopaminergic synapse model

The dopaminergic synapse which was proposed by Best et al.^166^ is nearly similar to the dopaminergic synapse in the proposed model in terms of richness of molecular details. However, there are some factors which gives the dopaminergic synapse in the proposed model an edge over the dopaminergic synapse proposed by Best et al.^166^. The following aspects of the dopaminergic synapse in the proposed model are listed below:

- *Calcium-dependent DA release*
  When an action potential arrives at the nerve terminal, it induces membrane depolarization, causing the opening of voltage-gated ion channels. The probability of release of DA storage vesicle in response to the nerve impulse depends on the conductance of calcium through N-type channels into the active zone^167^. Assuming that intracellular calcium concentration transients are identical at all DA release sites, we model intracellular calcium in the proposed as described in Eq. (54). Following the ideas in Lee et al.^168^ and assuming that calcium dependent DA release occurs within less than a millisecond after the calcium channels open^168,169^, the flux of DA release (Eq. 108) from the synapse is equal to the average release flux per vesicle (ψ) times the average number of vesicles in the readily releasable vesicle pool (nRRP, Eq. 109) multiplied by the release probability function which is a function of intracellular calcium as described in Eq. (111). Hence, the dopaminergic synapse in the proposed model was effortlessly integrated to dopaminergic soma model. In this integrated model, calcium oscillations in the soma are driven by ion channel activity, that modulates the DA release from the synapse which is not possible with dopaminergic synapse model proposed by Best et al.^166^.
- *Calcium-dependent DA synthesis*
  DA synthesis originates from the concentration of TYR located in the terminal bouton and is divided into two steps. Each of the steps depends on a specific enzyme that acts as a catalyst for that step. The first, a rate limiting step, is the catalysation of the hydroxylation of TYR by the enzyme TH to L-DOPA involving biopterin as its cofactor (Eq. 117). The activity of TH is regulated by a balance among cytosolic DA that acts as an end product inhibitor by competing with its cofactor, by extracellular DA that acts as an inhibitor via the binding with synthesis modulating autoreceptors located on the nerve terminals, and by neuronal activity as a stimulator^170,171^. The second step in the synthesis process is the catalysation of L-DOPA by AADC to DA (Eq. 122). As stated earlier, the activity of TH is regulated by neuronal activity. In the proposed model of dopaminergic synapse, neuronal stimulation is linked to the synthesis of DA as described in Eq. (118).
- *DA-modulated autoreceptors*
  There are four types of DA autoreceptors on the SNc neurons, where they regulate neuronal activity and control DA synthesis, release, and uptake^22^. When these autoreceptors get activated, they result in reduced neuronal activity, DA synthesis, release, and uptake. In the present SNc model, we have considered autoreceptors that regulate DA synthesis and release and excluded one regulating DA uptake (unable to find specific data regarding DA-mediated activation of autoreceptors which regulates DA reuptake) and neuronal activity. However, DA regulating neuronal activity can be incorporated in network model of SNc neurons where DA regulates the lateral connections (collaterals).

### Potential experimental setup to validate predictions from the proposed model

We suggest some experimental approaches to evaluate the behavior of dopaminergic neurons at single-cell or network level by capturing the dynamics of critical molecular players in various conditions. During energy-deficient conditions, monitoring important intracellular players such as ATP, glucose, AMP-activated protein kinase (AMPK), and lactate using single-cell imaging studies gives an insight into the progressive adaptation of dopaminergic neurons to the energy crisis by activating various compensatory mechanisms^52,172^. Also, we can determine all the cellular processes that are compromised during energy crisis. Mitochondria play a major role in maintaining cellular energy levels^173^, and monitoring its functioning capacity provides insights into cellular energy production. Using cellular models^174^, monitoring the mitochondrial calcium, ATP, NADPH, pH, membrane potential, oxygen consumption rate, ROS production, and morphology gives a better understanding of mitochondrial bioenergetic function in the neuron under energy deficits, oxidative stress, and excitotoxicity^4,174–176^. During progressive energy deficiency, DA and its metabolites can be measured to check for production of ROS leading to oxidative stress in the neuron using toxin-induced animal pathological models^177,178^.

### Future directions

In the proposed model, ketone metabolism^179^ can be incorporated to make the model more robust to utilize different substrates as an energy source and understand the role of ketone bodies in PD pathogenesis^180,181^. Apart from ketone bodies, astrocytes also play an important role in maintaining neuronal energy demands^182^. Therefore, combining the SNc neuronal model with astrocyte model will provide a better understanding of compensation due to astrocyte involvement in energy deficit conditions^183^. The ischemic condition was implemented by modulating glucose and oxygen levels, which can be extended by introducing the vascular module^184^, where ischemia condition can be simulated more realistically by varying cerebral blood flow. Cancer cells survive in low oxygen and acidic conditions^185^, where pH plays a vital role in the functioning of cellular processes^186^; thus, considering potentiometric properties in formulating cellular processes could be more biologically realistic (pH plays an essential role in mitochondrial functioning).

## Conclusions

In conclusion, we believe that the proposed model provides an integrated modelling framework to understand the neurodegenerative processes underlying PD^14^. From the simulation results, it was observed that under conditions of energy starvation, intracellular calcium, DA (cytoplasmic), alpha-synuclein, and ROS concentrations significantly deviated from normal values (equilibrium). There is a positive feedback loop formed with increased intracellular calcium, or DA levels lead to oligomerization of alpha-synuclein, while alpha-synuclein oligomers increased intracellular calcium and DA levels^91^. Any therapeutics that can reduce these key toxicity mediators can be beneficial for the survival of SNc neurons^59,91,187^. To this end, it is desirable to develop a therapeutic computational testbench for PD, wherein the proposed model of SNc will be the center of a larger framework, which will also be integrated to behavioral model^188^. This type of framework will help in providing personalized medicine for PD patients^189^ rather than the currently employed trial and error approaches.

## Supplementary Information

Supplementary Information.

## Data Availability

The comprehensive SNc model code (http://modeldb.yale.edu/265591) is available in ModelDB database^190^.
