# Supplementary material for: Influence of energy deficiency on the subcellular processes of Substantia Nigra Pars Compacta cell for understanding Parkinsonian neurodegeneration
Source: Sci Rep. 2021 Jan 18;11:1754. doi: 10.1038/s41598-021-81185-9 (PMC7814067; doi:10.1038/s41598-021-81185-9)
Supplement: Supplementary file 1 — Supplementary Information. [file 41598_2021_81185_MOESM1_ESM.pdf]

Supplementary Information

Influence of Energy Deficiency on the Subcellular Processes of *Substantia Nigra Pars Compacta* Cell for Understanding Parkinsonian Neurodegeneration

Vignayanandam Ravindernath. Muddapu[1], V. Srinivasa Chakravarthy[1 *];

[1]Computational Neuroscience Lab, Department of Biotechnology, Bhupat and Jyoti Mehta School of Biosciences, Indian Institute of Technology Madras, Chennai, India.

***Correspondence:** V. Srinivasa Chakravarthy: schakra@ee.iitm.ac.in

SUPPLEMENTARY FIGURES

Supplementary Figure-1

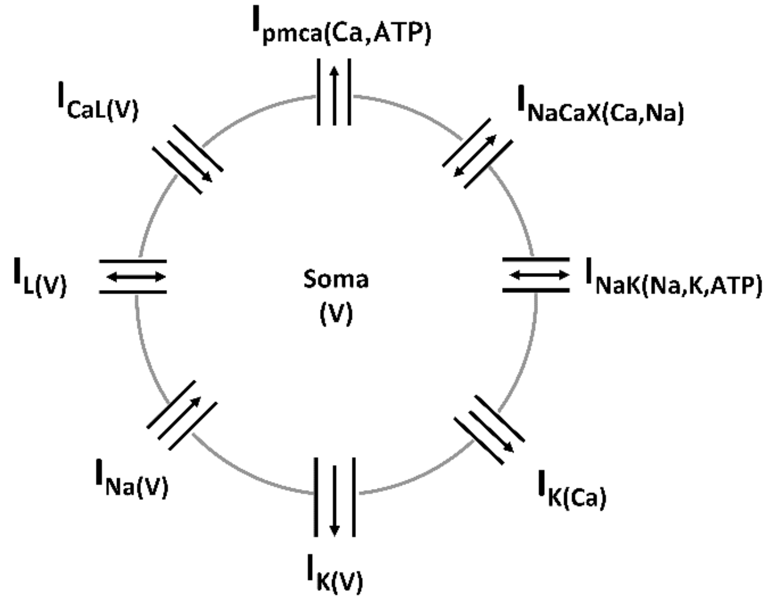

SF-1: Schematic of the single-compartment DA neuron model demonstrating the various ion currents in the proposed model of SNc cell[1]. *See main article for description of the figure.*

Supplementary Figure-2

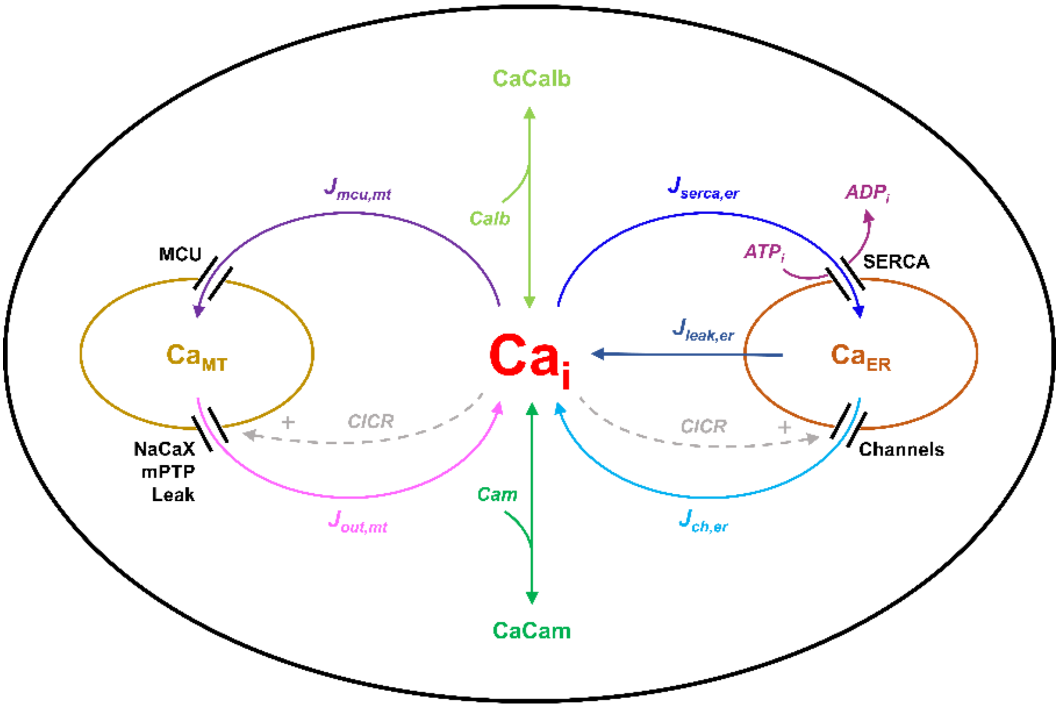

SF-2: Schematic of calcium buffering mechanisms in the proposed model of SNc cell*[1,2]*. *See main article for description of the figure.*

Supplementary Figure-3

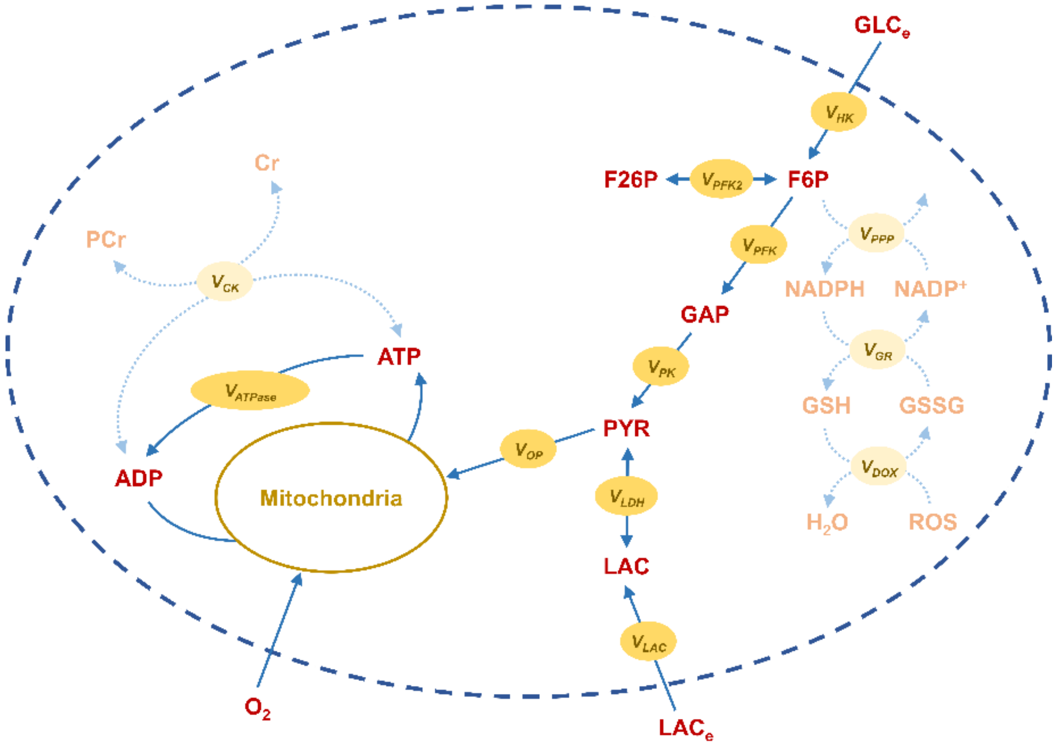

SF-3: Schematic of energy mechanism pathways in the proposed model of SNc cell*[3,4]*. *See main article for description of the figure.*

Supplementary Figure-4

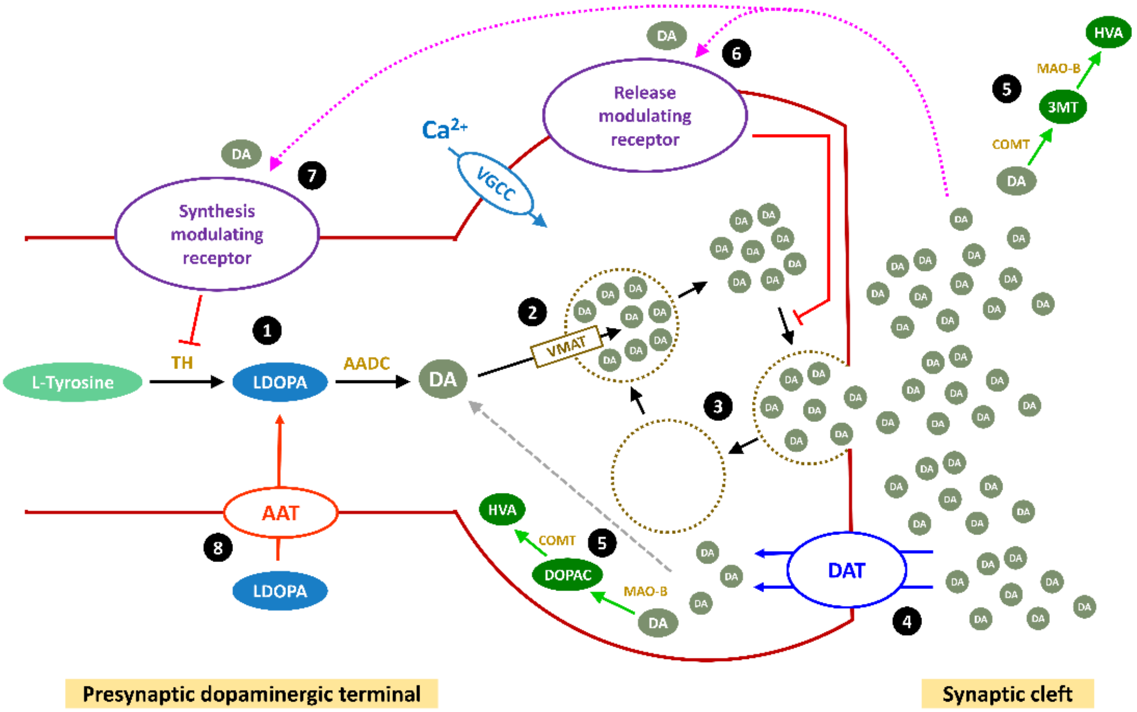

SF-4: Schematic of Dopamine turnover processes in the proposed model of SNc cell[5,6]. *See main article for description of the figure.*

Supplementary Figure-5

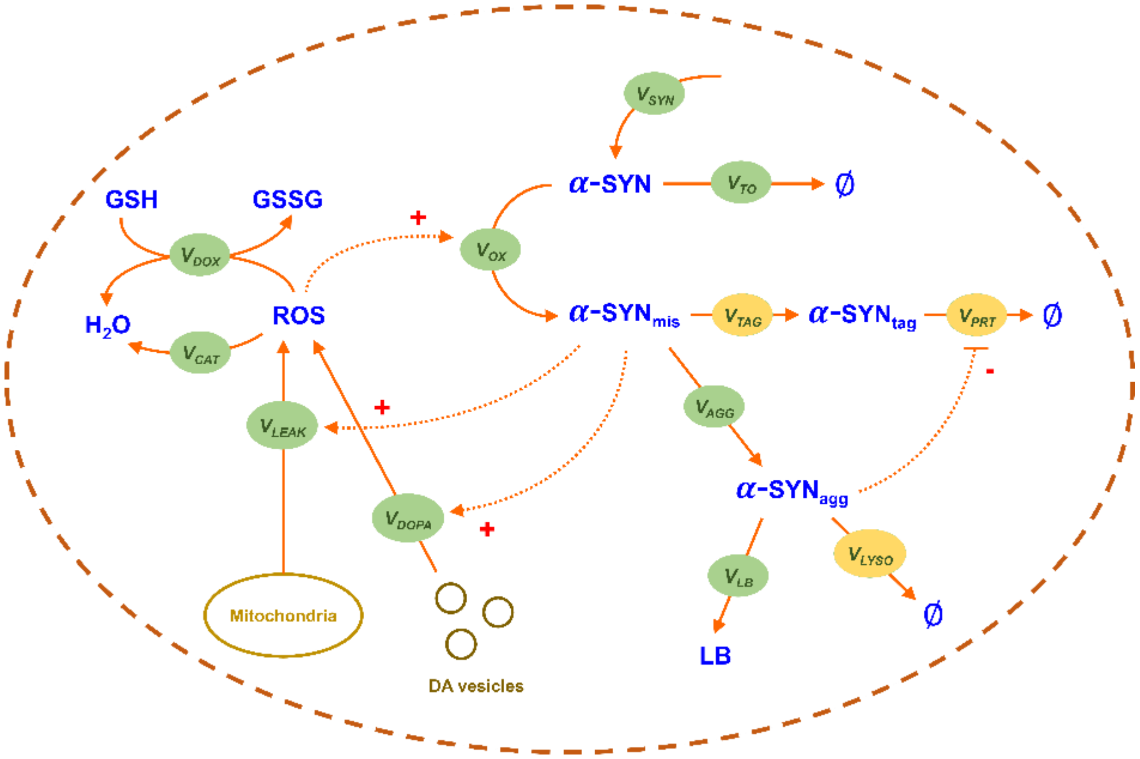

SF-5: Schematic of molecular pathways in PD pathology in the proposed model of SNc cell*[3,4]*. *See main article for description of the figure.*

Supplementary Figure-6

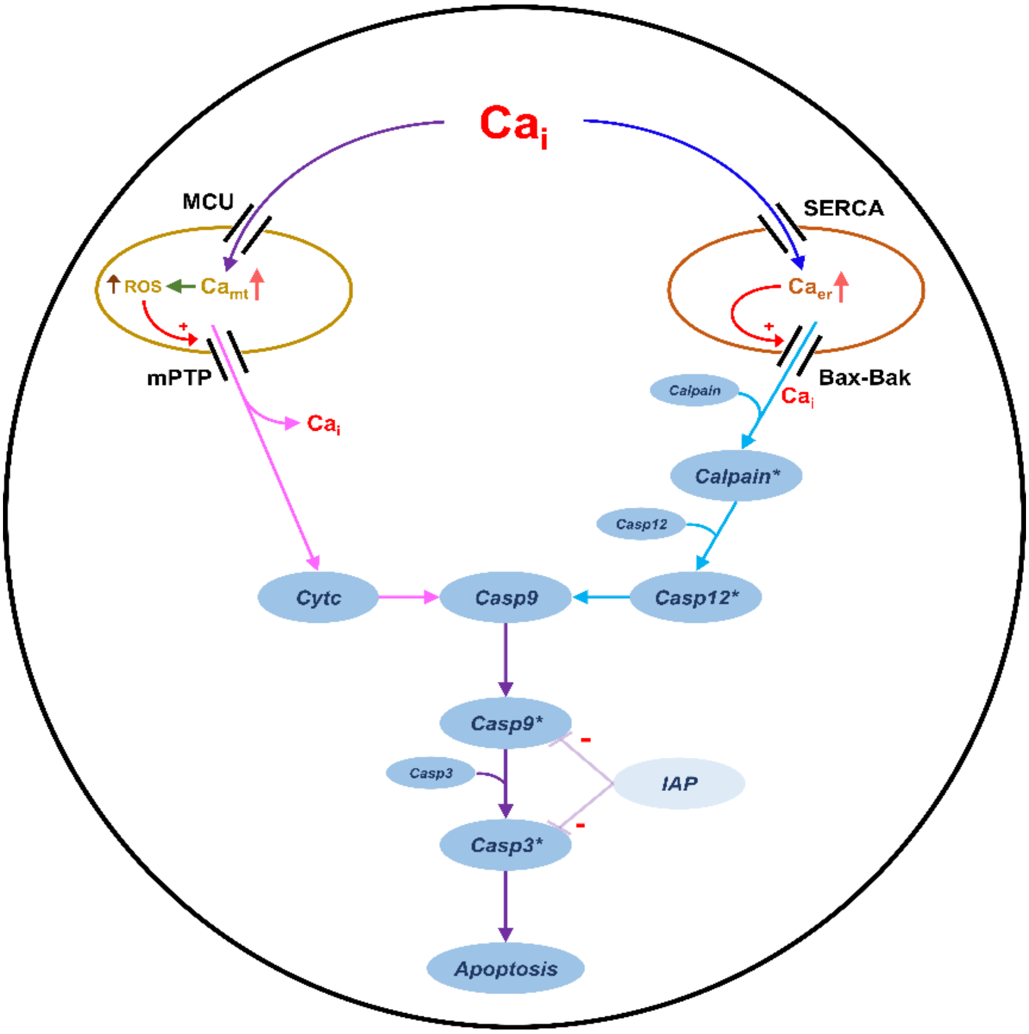

SF-6: Schematic of Apoptotic pathways in the proposed model of SNc cell[2,7]***.*** *See main article for description of the figure.*

Supplementary Figure-7

In the proposed model, ATP production by mitochondria was formulated in a single differential equation (Eq. 77) where electron transport system components were simplified to single parameter $\bar{\eta}_{op}$ which represents maximal electron transport chain efficiency. To study the effect of electron transport system components, this paramter was varied and average basal ATP level in the cytoplasm was monitored in the model (Suppl. Fig. 7). As the electron transport system efficiency decreases, the average basal ATP level also decreases. The basal ATP level stabilizes at $1.22\ mM$ for $\bar{\eta}_{op}$ values lower than 0.001 and the non-mitochondrial ATP production pathways (such glycolysis, ATP–creatine phosphate system etc.) contribute to the resultant ATP level.

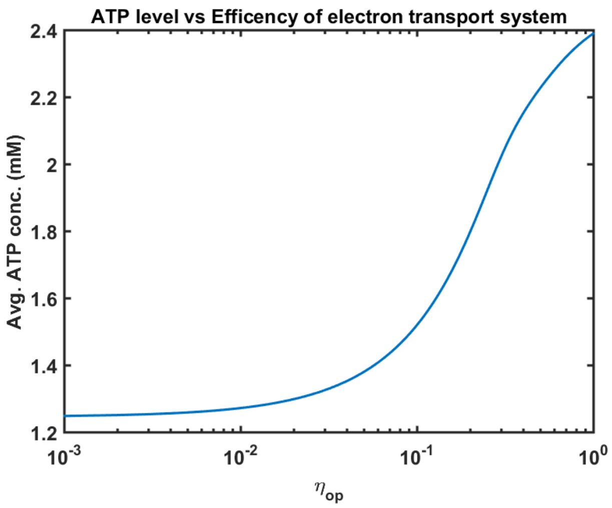

SF-7: Average basal ATP levels as a function $\bar{\eta}_{op}$ in the proposed model of SNc.

Supplementary Figure-8

In the present model, the TH activity is only regulated by extracellular and cytoplasmic dopamine (Eq. 117) and the other regulatory effects were simulated by modulating a parameter $\bar{V}_{synt}$ which represents maximal flux for levodopa (LDOPA) synthesis (where LDOPA is converted to dopamine instantaneously by aromatic l-amino acid decarboxylase). To study the effect of TH activity on dopamine turnover processes, this parameter was varied and different molecular players (cytoplasmic dopamine, vesicular dopamine, extracellular dopamine, cytoplasmic LDOPA and cytoplasmic reactive oxygen species (ROS)) in dopamine turnover processes were monitored in the model (Figure 2). As $\bar{V}_{synt}$ increases, LDOPA increases which leads to increased cytoplasmic dopamine. Increased cytoplasmic dopamine levels result in increased influx of dopamine from cytoplasm into vesicles which leads to increased vesicular dopamine levels resulting in increased release of dopamine into the extracellular space. Due to increased cytoplasmic dopamine, ROS levels increase as a result of excess dopamine. Furthermore, dopamine that did not sequester into vesicles undergoes autooxidation leading to elevated ROS production which results in oxidative stress-induced neuronal death.

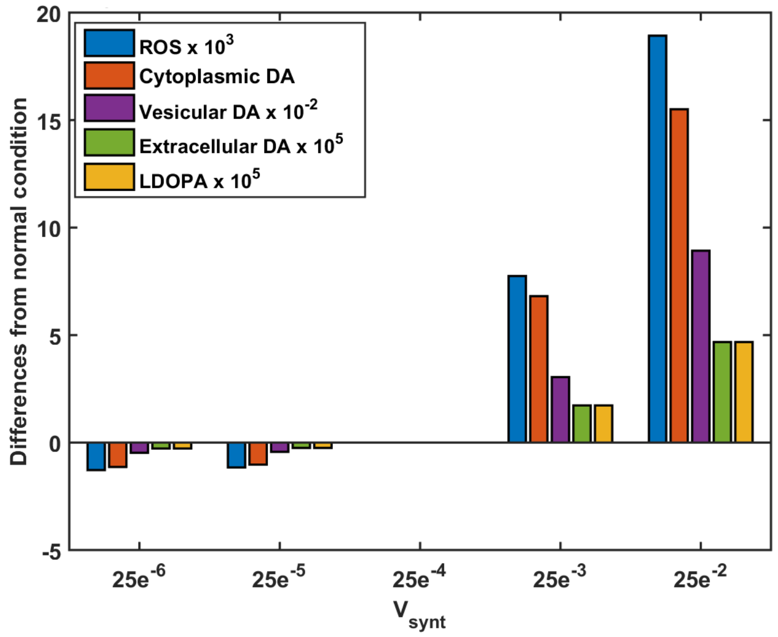

SF-8: Effect of $\overline{V}_{synt}$ on different molecular players in dopamine turnover processes in the proposed model of SNc cell.

Supplementary Figure-9

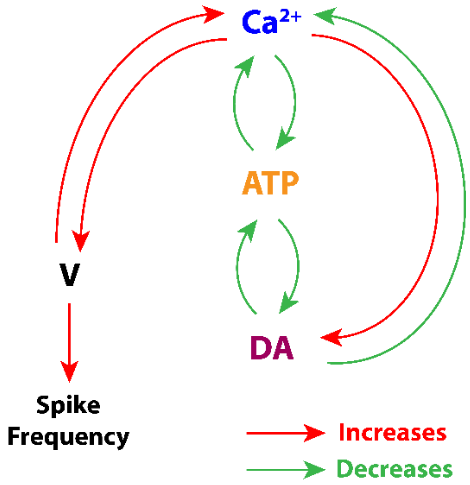

SF-9: Schematic diagram of the interaction between different important players in the proposed model of SNc cell. V, membrane potential voltage; Ca^{2+}, cytoplasmic calcium concentration; ATP, cytoplasmic adenosine triphosphate; DA, extracellular dopamine.

Supplementary Figure-10

At the level of single-cell studies, the neuron model exhibits multiple states, as it is shown in figure 8A; that model exhibits four dynamic regimes (in the model, high basal ATP and low

basal ATP states were observed) in which SNc neuron operates under different energy conditions. The four dynamic regimes are determined by how the basal ATP level behaves under different glucose and oxygen values. The region A was attributed to glucose and oxygen values for which no change in basal ATP level was observed. The region B was attributed to glucose and oxygen values for which there was an initial drop and subsequent return to basal ATP level. The region C was attributed to glucose and oxygen values for which there was an initial drop and a subsequent stabilization at a lower basal ATP level. The region D was attributed to glucose and oxygen values for which basal ATP level fluctuates (between high and low basal ATP levels). The region E was attributed to glucose and oxygen values for which cell undergoes degeneration (Suppl. Fig. 10).

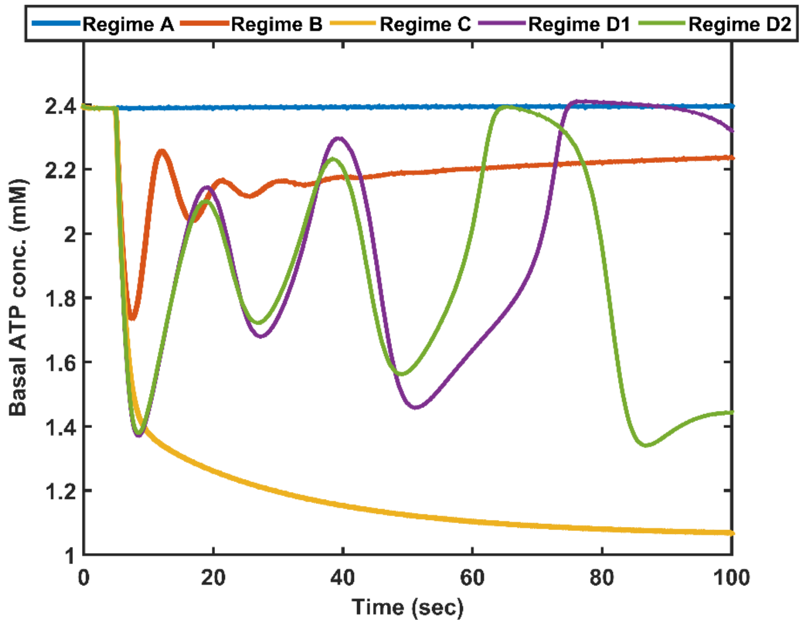

SF-10: Different dynamic regimes in the proposed model of SNc cell under energy deficiency conditions - Basal ATP patterns.

SUPPLEMENTARY TABLES

Supplementary Table-1: Published dopaminergic neuronal models.

**S.No.**	**Model**	**Ion channels**	**Pumps and exchangers (Ionic balance)**	**Synaptic currents**	**Reference(s)**
1.	Two-compartment – soma and dendrite	Soma: $I_{K,DR}$, I_{Na}<br>Dendrite: I_L	Dendrite: I_{NaKP}<br>(sodium)	Dendrite: I_{NMDA}	8
2.	Single-compartment soma with calcium buffering (CBP)	Soma: $I_{Ca,T}$, $I_{Ca,L}$, $I_{Ca,N}$, $I_{Ca,HVA}$, $I_{K,Ca}$, $I_{K,DR}$, $I_{K,A}$, I_H, I_B	Soma: I_{NaKP}, I_{CaP}, I_{NaCaX}<br>(calcium)	-	9
3.	Three compartments – Soma, proximal and distal dendrites	$I_{K,DR}$, I_L	I_{NaKP}<br>(sodium in all)	Distal dendrite: I_{NMDA}, I_{AMPA}, I_{GABAA}	10
4.	[9] model with calcium diffusion (also abstract version)	Soma: I_{Ca}, $I_{K,Ca}$, I_K, I_L	Soma: (calcium)	-	11–13
5.	Two [10] models coupled at distal dendrites	I_{Na}, $I_{K,DR}$, I_L, $I_{K,A}$	I_{NaKP}	Distal dendrite: I_{NMDA}	14
6.	Soma with four identical branched dendrites with a single proximal and two distal branches	Soma: I_{Na}, $I_{K,A}$, $I_{K,DR}$, I_L, $I_{K,Ca}$, $I_{Ca,T}$, $I_{Ca,L}$, $I_{Ca,N}$	Soma: I_{NaKP}, I_{CaP}<br>(calcium)<br>Dendrite: I_{NaKP}	Soma: I_{GABAA}<br>Dendrite: I_{NMDA}, I_{GABAA}	15

		Dendrite: I_{Na}, $I_{K,A}$, $I_{K,DR}$, I_L			
7.	Modified [15] model with I_{AMPA} synaptic current in dendrite	Soma: I_{Na}, $I_{K,A}$, $I_{K,DR}$, $I_{K,Ca}$, I_L, $I_{Ca,T}$, $I_{Ca,L}$, $I_{Ca,N}$<br>Dendrite: I_{Na}, $I_{K,A}$, $I_{K,DR}$, I_L	Soma: I_{NaKP}, I_{CaP}<br>(calcium)<br>Dendrite: I_{NaKP}<br>(sodium)	Soma: I_{GABAA}<br>Dendrite: I_{NMDA}, I_{AMPA}, I_{GABAA}	16
8.	Modified [11] model with I_{AMPA} and I_{NMDA} synaptic currents along with spiking generating ion channels	Soma:I_{Ca}, $I_{K,Ca}$, I_K, I_L, I_{Na}, $I_{K,DR}$	Soma: (calcium)	Soma: I_{NMDA}, I_{AMPA}	17
9.	Single-compartment soma	Soma: $I_{Ca,L}$, $I_{Ca,B}$, $I_{K,ERG}$, $I_{K,Ca}$, I_H, I_L	Soma: I_{CaP}<br>(calcium)	-	18
10.	Modified [15] model with pacemaking mechanism throughout soma and dendrites	Soma: I_{Na}, I_A, $I_{K,DR}$, I_L, $I_{K,Ca}$, $I_{Ca,L}$<br>Dendrite: I_{Na}, I_A, $I_{K,DR}$, I_L, $I_{K,Ca}$, $I_{Ca,L}$	Soma: (calcium)	-	19,20
11.	Single-compartment soma	Soma: $I_{Ca,L}$, I_{Na}, $I_{K,DR}$, $I_{K,Ca}$, I_L	Soma: I_{CaP}<br>(calcium)	-	21

12.	Single-compartment soma which is combines conductance mechanisms from [9] and [17]	Soma: $I_{Ca,L}$, I_{Na}, $I_{K,DR}$, $I_{K,Ca}$, I_L, I_K	Soma: I_{CaP} (calcium)	Soma: I_{NMDA}, I_{GABAA}	[22]
13.	Single-compartment soma with calcium buffering (CBP)	Soma: $I_{Ca,L}$, I_{Na}, $I_{Na,HCN}$, $I_{L,Na}$, $I_{K,DR}$, $I_{L,IR}$, $I_{K,Ca}$	Soma: I_{NaKP}, I_{CaP}, I_{NaCaX} (calcium, sodium, potassium, calbindin, calmodulin)	-	[1]
14.	Modified [17] model with altered NMDA and $I_{K,ERG}$ along with full morphology of dendrite (reduced model)	Soma: $I_{Ca,L}$, I_{Na}, $I_{K,DR}$, $I_{K,Ca}$, I_L, $I_{K,ERG}$	Soma: I_{CaP} (calcium)	Soma: I_{NMDA}, I_{AMPA}	[23,24]
15.	Single-compartment soma	Soma: I_{Na}, $I_{K,DR}$, I_L,	-	Soma: I_{NMDA}, I_{AMPA}	[25]
16.	Single-compartment soma with full morphology of dendrite	Soma: I_{Ca}, I_{Na}, $I_{K,DR}$, $I_{K,Ca}$, $I_{L,Ca}$, $I_{K,ERG}$, I_H, I_L	Soma: I_{CaP} (calcium)	-	[26]
17.	Simple (spiking) dopaminergic neuronal model	Izhikevich (point neuron) – two variable neuronal model	-	-	[27,28]

18.	Modified [23]	Soma: I_{Ca}, I_{Na}, $I_{Na,S}$, $I_{K,DR}$, $I_{K,Ca}$, I_L, I_K, I_H	Soma: I_{CaP} (calcium)	Soma: I_{NMDA}, I_{AMPA}, I_{GABAA}	29,30
19.	Single-compartment soma with calcium buffering (CBP) along $I_{K,ATP}$ mediated bursting	Soma: $I_{Ca,L}$, I_{Na}, $I_{K,DR}$, $I_{K,ATP}$, $I_{L,Ca}$, I_L	Soma: I_{CaP} (calcium)	Soma: I_{NMDA}	31
20.	Modified [19] model	$I_{Ca,L}$, $I_{Ca,T}$, I_{Na}, $I_{Na,HCN}$, $I_{K,DR}$, $I_{K,B}$, $I_{K,Ca}$, $I_{K,A}$, $I_{K,ERG}$, I_L	Soma: I_{CaP} (calcium)	-	32

$I_{Ca,T}$ – T-type calcium current; $I_{Ca,L}$ – L-type calcium current; $I_{Ca,N}$ – N-type calcium current; $I_{Ca,HVA}$ – residual high-voltage activated calcium current; I_{Ca} – calcium current; $I_{K,Ca}$ – calcium-activated (small conductance) potassium current; $I_{K,DR}$ – delayed rectifier potassium current; $I_{K,A}$ –transient outward (4-aminopyridine-sensitive) potassium current; I_H – hyperpolarization-activated cation current; I_B – background current (sodium, potassium, calcium); I_{NaKP} – sodium-potassium pump; I_{CaP} – calcium pump; I_{NaCaX} – sodium-calcium exchanger; I_L – leaky current; I_{Na} – fast spiking (tetrodotoxin-sensitive) sodium current; I_{NMDA} – N-methyl-D-aspartic acid (NMDA) current; I_{AMPA} – alpha-amino-3-hydroxy-5-methyl-4-isoxazolepropionic acid (AMPA) current; I_{GABAA} – gamma-aminobutyric acid A-class (GABAA) current; $I_{K,ERG}$ – ERG (ether-a-go-go-related gene) potassium current; $I_{Ca,B}$ – background calcium leak current; $I_{L,Ca}$ – leaky calcium current; CBP – calcium-binding proteins; $I_{L,Na}$ – leaky sodium current; $I_{K,IR}$ – inward rectifying potassium current; $I_{Na,HCN}$ – hyperpolarization-activated cyclic nucleotide (HCN) sodium current; $I_{Na,S}$ – subthreshold sodium current; I_K – intrinsic potassium current; $I_{K,ATP}$ – ATP-sensitive potassium current; $I_{K,B}$ – large conductance potassium current;

Supplementary Table-2: Published dopaminergic terminal models.

S.No.	Model	Metabolite balance (units)	Autoreceptors	Reference(s)
1.	Two-compartment – cytoplasmic and extracellular	DA_c, DA_v, DA_e, $LDOPA$, $I1$, $I2$<br><br>(nmol/g, min)	DA synthesis	[33]
2.	Two-compartment – cytoplasmic and extracellular	DA_i, DA_e<br><br>(μg/g, min)	DA firing	[34]
3.	Three-compartment – cytoplasmic, vesicular and extracellular	DA_c, DA_v, DA_e, DA_a, DA_g, $3MT$, $LDOPA$, $DOPAC$, HVA<br><br>(mM, ms)	-	[35]
4.	Biochemical systems theory model	DA homeostasis<br><br>(relative units)	-	[36,37]
5.	Three-compartment – cytoplasmic, vesicular and extracellular	DA_c, DA_v, DA_e, TYR, $LDOPA$, BH_2, BH_4, HVA, $TYRPOOL$<br><br>(μM, hr)	DA synthesis	[38,39]
6.	Three-compartment – cytoplasmic, vesicular and extracellular	DA_c, DA_v, DA_e<br><br>(mM, ms)	DA synthesis, DA release	[6]

7.	Modified [38] model with DA and 5HT cell bodies and 5HT terminal	DA terminal: DA_c, DA_v, DA_e, TYR, $LDOPA$, BH_2, BH_4, HVA, $TYRPOOL$<br><br>5HT terminal: $5HT_c$, $5HT_v$, $5HT_e$, $TRYP$, $5HTP$, BH_2, BH_4, HIA, $TRPPOOL$<br><br>(μM, hr)	DA synthesis, DA firing, 5HT synthesis, 5HT firing	5
8.	DA neurotransmission model	Volume transmission<br><br>(μM, sec)	DA firing, DA release	40,41
9.	Systems Biology Markup Language model	Flux balance analysis<br><br>(μM, hr)	-	42
10.	Modified [38] model with spiking neuronal model	DA terminal: DA_c, DA_v, DA_e, TYR, $LDOPA$, BH_2, BH_4, HVA, $TYRPOOL$<br><br>(μM, hr)	DA synthesis, DA firing	27

DA – dopamine; $5HT$ – serotonin; DA_c – cytoplasmic DA; DA_v – vesicular DA; DA_e – extracellular DA; DA_a – inactive DA; DA_g – glial DA; TH – tyrosine hydroxylase; $I1$ – competitive TH inhibitor 1; $I2$ – competitive TH inhibitor 2; $LDOPA$ – 3,4-dihydroxyphenylalanine; $3MT$ – 3-methoxytyramine; $DOPAC$ – 3,4-dihydroxyphenylacetic acid; HVA – homovanillic acid; TYR – tyrosine; BH_2 – dihydrobiopterin; BH_4 – tetrahydrobiopterin; $TYRPOOL$ – tyrosine pool; $5HT_c$ – cytoplasmic 5HT; $5HT_v$ – vesicular 5HT; $5HT_e$ – extracellular 5HT; $5HTP$ – 5-hydroxytryptophan; HIA – 5-hydroxyindoleacetic acid; $TRYP$ – tryptophan; $TRYPPOOL$ – tryptophan pool; μM – micromolar; mM – millimolar;

ms – millisecond; hr – hour; DA_i – intracellular DA; nmol – nanomole; g – gram; min – minute.

Supplementary Table-3:

Supplementary Table-3.1: Parameter values for ion-channel dynamics of SNc cell model[1].

Constant	Symbol	Value	Units
Faraday's constant	F	96485	$coulomb * mole^{-1}$
SNc membrane capacitance	C_{snc}	$9\ x\ 10^7$	$pF * cm^{-2}$
Cytosolic volume	vol_{cyt}	$\phi_{cyt} * vol_{pmu}$	pl
Fraction of cytosolic volume	ϕ_{cyt}	0.5	$dimensionless$
Pacemaking unit (PMU) volume	vol_{pmu}	5	pl
PMU area	AR_{pmu}	$\delta_{pmu} * vol_{pmu}$	$pl * cm^{-1}$
PMU surface area-to-volume ratio	δ_{pmu}	$1.6667\ x\ 10^4$	cm^{-1}
Voltage defined thermodynamic entity	V_D	$\frac{V}{V_\tau}$	$dimensionless$
Temperature defined thermodynamic entity	V_τ	$\frac{R * T}{F}$	mV
Universal gas constant	R	8314.472	$mJ * mol^{-1} * K^{-1}$
Physiological temperature	T	310.15	K
Maximal conductance of calcium channel	$\bar{g}_{Ca,L}$	2101.2	$pA * mM^{-1}$
Extracellular calcium concentration	$[Ca_e]$	1.8	mM
Reversal potential for calcium ion	V_{Ca}	$\frac{1}{2} * \log\left(\frac{[Ca_e]}{[Ca_i]}\right)$	$dimensionless$
Valence of calcium ion	z_{Ca}	2	$dimensionless$

Maximal conductance of sodium channel	$\bar{g}_{Na}$	907.68	$pA * mM^{-1}$
Extracellular sodium concentration	$[Na_e]$	137	mM
Reversal potential for sodium ion	V_{Na}	$\log\left(\frac{[Na_e]}{[Na_i]}\right)$	$dimensionless$
Valence of sodium ion	z_{Na}	1	$dimensionless$
Maximal conductance of sodium HCN channel	$\bar{g}_{NaHCN}$	51.1	$pA * mM^{-1}$
Maximal conductance of leaky sodium channel	$\bar{g}_{Nalk}$	0.0053	$pA * mM^{-1}$
Cyclic adenosine monophosphate concentration	$[cAMP]$	$1\ x\ 10^{-5}$	mM
Maximal conductance of delayed rectifying potassium channel	$\bar{g}_{Kdr}$	31.237	nS
Extracellular potassium concentration	$[K_e]$	5.4	mM
Reversal potential for potassium ion	V_K	$\log\left(\frac{[K_e]}{[K_i]}\right)$	$dimensionless$
Valence of potassium ion	z_K	1	$dimensionless$
Maximal conductance of inward rectifying potassium channel	$\bar{g}_{Kir}$	13.816	nS
Maximal conductance of small conductance potassium channel	$\bar{g}_{Ksk}$	2.2515	$pA * mM^{-1}$
Maximal conductance for sodium-potassium ATPase	K_{nak}	1085.7	pA
Reaction rates of I_{NaK}	$k_{2,nak}$	0.04	ms^{-1}

	$k_{3,nak}$	0.01	ms^{-1}
	$k_{4,nak}$	0.165	ms^{-1}
Dissociation constants of I_{NaK}	$K_{nak,nae}$	69.8	mM
	$K_{nak,nai}$	4.05	mM
	$K_{nak,ke}$	0.258	mM
	$K_{nak,ki}$	32.88	mM
Maximal conductance for calcium ATPase	k_{pmca}	2.233	$pA * ms^{-1}$
Reaction rates of I_{pmca}	$k_{2,pc}$	0.001	ms^{-1}
	$k_{3,pc}$	0.001	ms^{-1}
	$k_{4,pc}$	1	ms^{-1}
Dissociation constants of I_{pmca}	$K_{pc,e}$	2	mM
Maximal conductance for sodium-calcium exchanger	k_{xm}	0.0166	$pA * ms^{-1}$
Energy barrier parameter of I_{NaCaX}	δ_{xm}	0.35	$dimensionless$
Denominator factor of I_{NaCaX}	$\mathcal{D}_{xm}$	0.001	$dimensionless$

Supplementary Table-3.2: Steady state values of ion-channel dynamics of SNc cell model[1].

Symbol	Value	Symbol	Value
V	$-49.42\ mV$	h_{Na}	0.1848
$[Ca_i]$	$1.88\ x\ 10^{-4}\ mM$	O_{NaHCN}	0.003
$[Na_i]$	$4.69\ mM$	$m_{K,dr}$	0.003
$[K_i]$	$126.06\ mM$	y_{nak}	0.6213

m_{Na}	0.0952	y_{pc}	0.483

Supplementary Table-3.3: Parameter values of calcium buffering mechanisms of SNc cell model[1,2].

Constant	Symbol	Value	Units
Calbindin reaction rates	$k_{1,calb}$	10	$mM^{-1} * ms^{-1}$
	$k_{2,calb}$	$2\ x\ 10^{-3}$	ms^{-1}
Total cytosolic calbindin concentration	$[Calb_{tot}]$	0.005	mM
Calmodulin reaction rates	k_{cam}^{cb}	12000	$mM^{-2} * ms^{-1}$
	k_{cam}^{nb}	$3.7\ x\ 10^{6}$	$mM^{-2} * ms^{-1}$
	k_{cam}^{cd}	$3\ x\ 10^{-3}$	ms^{-1}
	k_{cam}^{nd}	3	ms^{-1}
Total cytosolic calmodulin concentration	$[Cam_{tot}]$	0.0235	mM
The maximal rate constant of SERCA	$k_{serca,er}$	0.02	$mM^{-1} * ms^{-1}$
Maximal permeability of calcium channels in the ER membrane	$k_{ch,er}$	3	ms^{-1}
Half saturation for calcium	$K_{ch,er}$	0.005	mM
Maximal rate constant for calcium leak flux through the ER membrane	$k_{leak,er}$	$5\ x\ 10^{-5}$	ms^{-1}
Ratio of free calcium to total calcium concentration in ER	β_{er}	0.0025	$dimensionless$

Volume ratio between the ER and cytosol	ρ_{er}	0.01	$dimensionless$
Maximal permeability of MCUs	$k_{mcu,mt}$	$3\ x\ 10^{-4}$	$mM * ms^{-1}$
Half saturation for calcium	$K_{mcu,mt}$	$8\ x\ 10^{-4}$	mM
Maximal rate of calcium flux through $[Na^{+}]/[Ca^{2+}]$ exchangers and mPTPs	$k_{out,mt}$	0.125	ms^{-1}
Half saturation for calcium	$K_{out,mt}$	0.005	mM
Maximal rate constant for calcium leak flux through the MT membrane	$k_{leak,mt}$	$6.25\ x\ 10^{-6}$	ms^{-1}
Ratio of free calcium to total calcium concentration in MT	β_{mt}	0.0025	$dimensionless$
Volume ratio between the MT and cytosol	ρ_{mt}	0.01	$dimensionless$

Supplementary Table-3.4: Steady state values of calcium buffering mechanisms of SNc cell model [1,2].

Symbol	Value	Symbol	Value
$[Ca_{er}]$	$1\ x\ 10^{-3}\ mM$	$[Calb]$	$26\ x\ 10^{-4}\ mM$
$[Ca_{mt}]$	$4\ x\ 10^{-4}\ mM$	$[Cam]$	$222\ x\ 10^{-4}\ mM$

Supplementary Table-3.5: Parameter values of energy metabolism of SNc cell model[3,4].

Constant	Symbol	Value	Units
Extracellular glucose concentration	$[GLC_e]$	1	mM
Hexokinase maximal flux	$\bar{v}_{hk}$	$2.5\ x\ 10^{-3}$	$mM * ms^{-1}$

Affinity constant for ATP	$K_{m,ATP,hk}$	0.5	mM
Inhibition constant for F6P	$K_{i,F6P}$	0.068	mM
Phosphofructokinase maximal flux	$\bar{v}_{pfk}$	$3.8\ x\ 10^{-3}$	$mM * ms^{-1}$
Affinity constant for F6P	$K_{m,F6P,pfk}$	0.18	mM
Affinity constant for ATP	$K_{m,ATP,pfk}$	0.05	mM
Affinity constant for F26P	$K_{m,F26P,pfk}$	0.01	mM
Activation constant for AMP	$K_{a,AMP,pfk}$	0.05	mM
Inhibition constant for ATP	$K_{i,ATP}$	1	mM
Coefficient constant for AMP	$nAMP$	0.5	$dimensionless$
Coefficient constant for ATP	$nATP$	0.4	$dimensionless$
Total energy shuttles concentration	$[ANP]$	2.51	mM
Coefficient constant for ADP	Q_{adk}	0.92	$dimensionless$
Phosphofructokinase-2 maximal forward flux	$\bar{v}_{pfk2,f}$	$2\ x\ 10^{-7}$	$mM * ms^{-1}$
Phosphofructokinase-2 maximal reverse flux	$\bar{v}_{pfk2,r}$	$1.036\ x\ 10^{-7}$	$mM * ms^{-1}$
Affinity constant for F6P	$K_{m,F6P,pfk2}$	0.01	mM
Affinity constant for ATP	$K_{m,ATP,pfk2}$	0.05	mM
Affinity constant for F26P	$K_{m,F26P,pfk2}$	0.0001	mM
Activation constant for AMP	$K_{a,AMP,pfk2}$	0.005	mM
Pyruvate kinase maximal flux	$\bar{v}_{pk}$	$5\ x\ 10^{-3}$	$mM * ms^{-1}$
Affinity constant for GAP	$K_{m,GAP,pk}$	0.4	mM
Affinity constant for ADP	$K_{m,ADP,pk}$	0.005	mM

Oxidative phosphorylation maximal flux	$\bar{v}_{op}$	$1\ x\ 10^{-3}$	$mM * ms^{-1}$
Maximal electron transport chain efficiency	$\bar{\eta}_{op}$	0.995	$dimensionless$
Maximal fraction of $asyn^{*}$ effect on the oxidative phosphorylation	$\beta_{op,asyn_{mis}}$	0.08	$dimensionless$
Affinity constant for $asyn^{*}$	$K_{asyn_{mis}}$	$8.5\ x\ 10^{-3}$	mM
Affinity constant for PYR	$K_{m,PYR,op}$	0.5	mM
Affinity constant for ADP	$K_{m,ADP,op}$	0.005	mM
Forward reaction constant of LDH	$k_{ldh,f}$	$12.5\ x\ 10^{-3}$	ms^{-1}
Reverse reaction constant of LDH	$k_{ldh,r}$	$2.5355\ x\ 10^{-3}$	ms^{-1}
Maximal lactate fermentation efficiency	$\bar{\eta}_{ldh}$	1	$dimensionless$
Maximal fraction of ROS effect on the lactate fermentation	$\beta_{ldh,ROS}$	0.25	$dimensionless$
Affinity constant for ROS	$K_{ldh,ROS}$	$10\ x\ 10^{-3}$	mM
MCT maximal influx	$\bar{v}_{lac}$	$3.55\ x\ 10^{-4}$	$mM * ms^{-1}$
Coefficient constant for MCT influx	$K_{lac,inf}$	0.641	$dimensionless$
Reaction constant for lactate efflux	$K_{lac,eff}$	$7.1\ x\ 10^{-4}$	ms^{-1}
ATPase maximal flux	$\bar{v}_{ATPase}$	$9.355\ x\ 10^{-4}$	$mM * ms^{-1}$
Affinity constant for ATP	$K_{m,ATP}$	0.5	mM
PPP maximal flux	$\bar{v}_{ppp}$	$3.972\ x\ 10^{-4}$	$mM * ms^{-1}$
Inhibition constant for $\left(\frac{NADPH}{NADP}\right)$	$K_{i,NADPH}$	20	$dimensionless$

Total NADPH and NADP concentration	$[NADPH_{tot}]$	0.25	mM
GR forward reaction constant	$k_{gr,f}$	$1.8\ x\ 10^{-4}$	$mM^{-1} * ms^{-1}$
GR reverse reaction constant	$k_{gr,r}$	$3.472\ x\ 10^{-7}$	$mM^{-1} * ms^{-1}$
Total GSH and GSSG concentration	$[GSH_{tot}]$	2.5	mM
Reaction constant of DOX	$K_{dox,ROS}$	$7.5\ x\ 10^{-8}$	ms^{-1}
CK forward reaction constant	$k_{ck,f}$	$3\ x\ 10^{-3}$	$mM^{-1} * ms^{-1}$
CK reverse reaction constant	$k_{ck,r}$	$1.26\ x\ 10^{-3}$	$mM^{-1} * ms^{-1}$
Total PCr and Cr concentration	$[PCr_{tot}]$	20	mM

Supplementary Table-3.6: Steady state values of energy metabolism of SNc cell model[3,4].

**Symbol**	**Value**	**Symbol**	**Value**
$[F6P]$	$0.176\ mM$	$[LAC]$	$0.598\ mM$
$[F26P]$	$2.2\ x\ 10^{-3}\ mM$	$[PCr]$	$18.04\ mM$
$[GAP]$	$8.25\ x\ 10^{-2}\ mM$	$[NADPH]$	$0.25\ mM$
$[PYR]$	$0.124\ mM$	$[GSH]$	$2.5\ mM$
$[ATP_i]$	$2.4\ mM$		

Supplementary Table-3.7: Parameter values for DA turnover processes of SNc cell model[5,6].

**Constant**	**Symbol**	**Value**	**Units**
Average release flux per vesicle	ψ	17.4391793	$mM * ms^{-1}$
Initial vesicular DA concentration	DA_{v_o}	500	mM

Sensitivity to vesicular DA concentration	DA_{v_s}	0.01	mM
Affinity constant of DA binding to receptors	DA_{R_a}	$5\ x\ 10^{-5}$	mM
Binding sensitivity	DA_{R_s}	0.01	mM
Activation constant for ATP	$K_{a,RRP}$	1.4286	mM
Vesicle recycling maximal flux	$\bar{v}_{nrrp}$	$1\ x\ 10^{-3}$	$mM * ms^{-1}$
Maximal vesicle recycling efficiency	$\bar{\eta}_{nrrp}$	0.995	$dimensionless$
Maximal fraction of $asyn^*$ effect on the vesicle	$\beta_{nrrp,asyn_{mis}}$	0.08	$dimensionless$
Affinity constant for $asyn^*$	$K_{asyn_{mis}}$	$8.5\ x\ 10^{-3}$	mM
Reaction constant of DA_e clearance	k_{comt}	0.0083511	ms^{-1}
Tyrosine concentration	$[TYR]$	$126\ x\ 10^{-3}$	mM
Affinity constant for TYR	K_{TYR}	$46\ x\ 10^{-3}$	mM
Inhibition constant for DA_c	$K_{i,cda}$	$11\ x\ 10^{-2}$	mM
Inhibition constant for DA_e	$K_{i,eda}$	$46\ x\ 10^{-3}$	mM
Maximal velocity of DA synthesis	$\bar{V}_{synt}$	$25\ x\ 10^{-6}$	$mM * ms^{-1}$
Affinity constant for Ca_i	K_{synt}	$35\ x\ 10^{-4}$	mM
Maximal velocity of VMAT	$\bar{V}_{cda}$	$4.67\ x\ 10^{-6}$	ms^{-1}
Affinity constant for DA_c	K_{cda}	$238\ x\ 10^{-4}$	mM
Scaling factor for VMAT	α_{vmat}	$1\ x\ 10^{-3}$	$dimensionless$
Scaling factor for ATP_i	β_{vmat}	3	$dimensionless$
Reaction constant of DA_c clearance	k_{mao}	0.00016	ms^{-1}
Maximal velocity of AADC	$\bar{V}_{aadc}$	$9.73\ x\ 10^{-5}$	$mM * ms^{-1}$

Affinity constant for $LDOPA$	K_{aadc}	0.13	mM
Maximal velocity of AAT	$\bar{V}_{aat}$	$5.11\ x\ 10^{-7}$	$mM * ms^{-1}$
Affinity constant for $LDOPA_e$	K_{ldopa_e}	$3.2\ x\ 10^{-4}$	mM
Affinity constant for TYR_e	K_{tyr_e}	$6.4\ x\ 10^{-4}$	mM
Affinity constant for TRP_e	K_{trp_e}	$1.5\ x\ 10^{-4}$	mM
Serum concentration of TYR	$[TYR_e]$	$6.3\ x\ 10^{-4}$	mM
Serum concentration of TRP	$[TRP_e]$	$8.2\ x\ 10^{-4}$	mM
Serum concentration of LDOPA	$[sLD]$	$3.6\ x\ 10^{-3}$	mM

Supplementary Table-3.8: Steady state values of DA turnover processes of SNc cell model[5,6].

Symbol	Value	Symbol	Value
$[DA_e]$	$4\ x\ 10^{-6}\ mM$	$[DA_v]$	$500\ mM$
$[DA_c]$	$1\ x\ 10^{-4}\ mM$	$[LDOPA]$	$3.6\ x\ 10^{-4}\ mM$

Supplementary Table-3.9: Parameter values of PD pathology pathways of SNc cell model[4].

Constant	Symbol	Value	Units
Activation constant for ATP	$K_{a,leak}$	0.5282	mM
Reaction constant for ROS production due to excess dopamine	k_{dopa}	$4.167\ x\ 10^{-4}$	$mM^{-1} * ms^{-1}$
Affinity constant for $[DA_c]$	K_{dopa}	8.5	mM
Reaction constant for catalase	k_{cat}	$2.35\ x\ 10^{-5}$	ms^{-1}
Reaction constant for alpha-synuclein oxidation	k_{syn}	$1.39\ x\ 10^{-8}$	$mM * ms^{-1}$

Reaction constant for alpha-synuclein consumption	k_{to}	$1.39\ x\ 10^{-7}$	ms^{-1}
Reaction constant for alpha-synuclein aggregation	k_{agg}	$2.08\ x\ 10^{-10}$	ms^{-1}
Affinity constant for $ASYN_{mis}$	K_{agg}	$7.5\ x\ 10^{-3}$	mM
Reaction constant for tagging of damaged protein	k_{tag}	$7.64\ x\ 10^{-11}$	$mM^{-1} * ms^{-1}$
Total ubiquitin concentration	$[Ub_{tot}]$	$10.5\ x\ 10^{-3}$	mM
Reaction constant for damaged protein disposal by the proteasome	k_{prt}	$2.08\ x\ 10^{-10}$	ms^{-1}
Affinity constant for $ASYN_{agg}$	K_{prt}	$5\ x\ 10^{-3}$	mM
Fraction reduction of proteasome activity by $ASYN_{agg}$	β_{prt}	0.25	$dimensionless$
Reaction constant for $ASYN_{agg}$ disposal by lysosome	k_{lyso}	$2.08\ x\ 10^{-11}$	ms^{-1}
Reaction constant for Lewy bodies from $ASYN_{agg}$	k_{lb}	$2.08\ x\ 10^{-11}$	ms^{-1}
Affinity constant for $ASYN_{agg}$	K_{lb}	$5\ x\ 10^{-3}$	mM

Supplementary Table-3.10: Steady state values of PD pathology pathways of SNc cell model[4].

Symbol	Value	Symbol	Value
$[ROS]$	$1\ x\ 10^{-3}\ mM$	$[ASYN_{tag}]$	$1\ x\ 10^{-5}\ mM$
$[ASYN]$	$0.1\ mM$	$[ASYN_{agg}]$	$0\ mM$
$[ASYN_{mis}]$	$1\ x\ 10^{-3}\ mM$	$[LB]$	$0\ mM$

Supplementary Table-3.11: Parameter values of apoptotic pathways of SNc cell model[7].

Constant	Symbol	Value	Units
Forward reaction constant for $[Ca_i.Calpain]$	k_1^+	1	$mM^{-1} * ms^{-1}$
Reverse reaction constant for $[Ca_i.Calpain]$	k_1^-	$1\ x\ 10^{-3}$	ms^{-1}
Forward reaction constant for $[Calpain^*]$	k_2^+	$1\ x\ 10^{-3}$	ms^{-1}
Forward reaction constant for $[Calpain^*.Casp12]$	k_3^+	1	$mM^{-1} * ms^{-1}$
Reverse reaction constant for $[Calpain^*.Casp12]$	k_3^-	$1\ x\ 10^{-3}$	ms^{-1}
Forward reaction constant for $[Casp12^*]$	k_4^+	$1\ x\ 10^{-3}$	ms^{-1}
Forward reaction constant for $[Casp12^*.Casp9]$	k_5^+	10	$mM^{-1} * ms^{-1}$
Reverse reaction constant for $[Casp12^*.Casp9]$	k_5^-	$5\ x\ 10^{-4}$	ms^{-1}
Forward reaction constant for $[Casp9^*]$	k_6^+	$1\ x\ 10^{-3}$	ms^{-1}
Forward reaction constant for $[Casp9^*.Casp3]$	k_7^+	10	$mM^{-1} * ms^{-1}$
Reverse reaction constant for $[Casp9^*.Casp3]$	k_7^-	$5\ x\ 10^{-4}$	ms^{-1}
Forward reaction constant for $[Casp3^*]$	k_8^+	$1\ x\ 10^{-4}$	ms^{-1}
Forward reaction constant for $[Apop]$	k_9^+	1	$mM^{-1} * ms^{-1}$
Forward reaction constant for $[Casp9^*]$	k_{10}^+	$1\ x\ 10^{-3}$	ms^{-1}
Forward reaction constant for $[Casp9^*.IAP]$	k_{11}^+	5	$mM^{-1} * ms^{-1}$
Reverse reaction constant for $[Casp9^*.IAP]$	k_{11}^-	$35\ x\ 10^{-7}$	ms^{-1}
Forward reaction constant for $[Casp3^*.IAP]$	k_{12}^+	5	$mM^{-1} * ms^{-1}$
Reverse reaction constant for $[Casp3^*.IAP]$	k_{12}^-	$35\ x\ 10^{-7}$	ms^{-1}
Forward reaction constant for $[ROS_{mit}]$	k_{13}^+	0.5	$mM^{-1} * ms^{-1}$
Forward reaction constant for $[PTP_{mit}^*]$	k_{14}^+	0.5	$mM^{-1} * ms^{-1}$
Forward reaction constant for $[Cytc]$	k_{15}^+	1	$mM^{-1} * ms^{-1}$

Forward reaction constant for $[Cytc.Casp9]$	k_{16}^{+}	1	$mM^{-1} * ms^{-1}$
Reverse reaction constant for $[Cytc.Casp9]$	k_{16}^{-}	$1\ x\ 10^{-3}$	ms^{-1}

Supplementary Table-3.12: Steady state values of energy metabolism of SNc cell model[7].

Symbol	Value	Symbol	Value
$[Calpain]$	1	$[ROS_{mit}]$	0
$[Ca_i.Calpain]$	0	$[PTP_{mit}^{*}]$	1
$[Calpain^{*}]$	0	$[Cytc_{mit}]$	1
$[Casp12]$	1	$[Cytc]$	0
$[Calpain^{*}.Casp12]$	0	$[Cytc.Casp9]$	0
$[Casp12^{*}]$	0	$[Casp9]$	1
$[Casp12^{*}.Casp9]$	0	$[Casp9^{*}]$	0
$[Casp3]$	1	$[Casp9^{*}.Casp3]$	0
$[Casp3^{*}]$	0	$[IAP]$	1
$[Casp9^{*}.IAP]$	0	$[Casp3^{*}.IAP]$	0
$[Apop]$	0		

Supplementary Table-3.13: Parameters for energy consumption processes of SNc cell model.

Constant	Symbol	Value	Units
Faraday's constant	F	96485	$coulomb * mole^{-1}$
Cytosolic volume	v_{cyt}	$\phi_{cyt} * v_{pmu}$	pl
Pacemaking unit (PMU) volume	v_{pmu}	5	pl
Fraction of cytosolic volume	ϕ_{cyt}	0.5	$dimensionless$

Scaling factor for synaptic recycling	λ_{sr}	100	$dimensionless$
Scaling factor for neurotransmitter packing	λ_{np}	1	$dimensionless$
Ratio of free calcium to total calcium concentration in ER	β_{er}	0.0025	$dimensionless$
Volume ratio between the ER and cytosol	ρ_{er}	0.01	$dimensionless$
Scaling factor for proteasome	λ_{prt}	25	$dimensionless$
Scaling factor for ubiquitination	λ_{tag}	3	$dimensionless$
Scaling factor for lysosome	λ_{lyso}	10	$dimensionless$

SUPPLEMENTARY MATERIALS

Supplementary Material-1

Receptor Modeling[43]

AMPA/Kainate Receptors

The simplest model that approximates the kinetics of the fast AMPA/kainate type of glutamate receptors can be represented by the two-state diagram:

$$C + T \overset{(\alpha/\beta)}{\Longleftrightarrow} O \quad (1)$$

where, α and β are voltage-independent forward and backward rate constants, C is the closed state of the receptor, O is the open state of the receptor, and T is the neurotransmitter. If r is defined as the fraction of the receptors in the open state, it is then described by the following first-order kinetic equation:

$$\frac{d(r)}{dt} = \alpha * [T] * (1 - r) - \beta * r \quad (2)$$

and the postsynaptic current (I_{AMPA}) is given by,

$$I_{AMPA} = \bar{g}_{AMPA} * r * (V - E_{AMPA}) \quad (3)$$

where, $\bar{g}_{AMPA}$ is the maximal conductance, E_{AMPA} is the reversal potential, V is the postsynaptic membrane potential, $[T]$ is the neurotransmitter, and r is the fraction of the receptors in the open state.

NMDA Receptors

The slower NMDA type of glutamate receptors can be represented with a two-state model similar to AMPA/kainate receptors, with a voltage-dependent term representing magnesium block. Using the scheme in Eqs. 1 and 2, the postsynaptic current is given by

$$I_{NMDA} = \bar{g}_{NMDA} * r * B(V) * (V - E_{NMDA}) \quad (4)$$

where, $\bar{g}_{NMDA}$ is the maximal conductance, E_{NMDA} is the reversal potential, $B(V)$ is the magnesium block, V is the postsynaptic membrane potential, and r is the fraction of the receptors in the open state.

$$B(V) = \frac{1}{1 + \left(\frac{[Mg^{2+}]}{3.57} * e^{-0.062 * V}\right)} \tag{5}$$

where, $[Mg^{2+}]$ is the external magnesium concentration, and V is the postsynaptic membrane potential.

$GABA_A$ Receptors

$GABA_A$ receptors can also be represented by the scheme in Eqs. 1 and 2, with the postsynaptic current given by

$$I_{GABA_A} = \bar{g}_{GABA_A} * r * \left(V - E_{GABA_A}\right) \tag{6}$$

where, $\bar{g}_{GABA_A}$ is the maximal conductance, E_{GABA_A} is the reversal potential, V is the postsynaptic membrane potential, and r is the fraction of the receptors in the open state.

$GABA_B$ Receptors

The stimulus dependency of $GABA_B$ responses, unfortunately, cannot be handled correctly by a two-state model. The simplest model of $GABA_B$-mediated currents has two variables:

$$\frac{d(r)}{dt} = K_1 * [T] * (1 - r) - K_2 * r \tag{7}$$

$$\frac{d(s)}{dt} = K_3 * r - K_4 * s \tag{8}$$

and the postsynaptic current $\left(I_{GABA_B}\right)$ is given by,

$$I_{GABA_B} = \bar{g}_{GABA_B} * \frac{s^n}{s^n + K_d} * \left(V - E_{GABA_B}\right) \tag{9}$$

where, $\bar{g}_{GABA_B}$ is the maximal conductance, E_{GABA_B} $(= V_K)$ is the reversal potential, V is the postsynaptic membrane potential, r is the fraction of the receptors in the open state, s is the fraction of activated G-proteins, K_d is the dissociation constant of the binding of s on the K^+ channels, K_1 and K_2 are voltage-independent forward and backward rate constants for r, K_3 and K_4 are voltage-independent forward and backward rate constants for s, and $[T]$ is the neurotransmitter.

Overall Synaptic Current

The overall synaptic input current flux (J_{syn}) to SNc neuron is given by,

$$J_{syn} = -\frac{1}{F * vol_{cyt}} * \left(I_{AMPA} + I_{NMDA} + I_{GABA_A} + I_{GABA_B}\right) \tag{10}$$

where, I_{AMPA} is the excitatory AMPA synaptic current, I_{NMDA} is the excitatory NMDA synaptic current, I_{GABA_A} is the inhibitory $GABA_A$ synaptic current, I_{GABA_B} is the inhibitory $GABA_B$ synaptic current, F is the Faraday's constant, and vol_{cyt} is the cytosolic volume.

Table-1: Parameter values of receptor models

**Constant**	**Symbol**	**Value**	**Units**
Faraday's constant	F	96485	$coulomb * mole^{-1}$
Cytosolic volume	vol_{cyt}	$\phi_{cyt} * vol_{pmu}$	pl
Fraction of cytosolic volume	ϕ_{cyt}	0.5	$dimensionless$
Pacemaking unit (PMU) volume	vol_{pmu}	5	pl
Maximal conductance of AMPA receptor	$\bar{g}_{AMPA}$	$0.35 - 1$	nS
Maximal conductance of NMDA receptor	$\bar{g}_{NMDA}$	$0.01 - 0.6$	nS
Concentration of Magnesium	$[Mg^{2+}]$	$1 - 2$	mM

Maximal conductance of $GABA_A$ receptor	$\bar{g}_{GABA_A}$	$0.25 - 1.2$	nS
Maximal conductance of $GABA_B$ receptor	$\bar{g}_{GABA_B}$	0.06	nS
Dissociation constant of the binding of s on the K^+ channels	K_d	100	μM^4
Voltage-independent forward rate constant for r of $GABA_B$	K_1	$9\ x\ 10^4$	$M^{-1} * sec^{-1}$
Voltage-independent backward rate constant for r of $GABA_B$	K_2	1.2	sec^{-1}
Voltage-independent forward rate constant for s of $GABA_B$	K_3	180	sec^{-1}
Voltage-independent backward rate constant for s of $GABA_B$	K_4	34	sec^{-1}
Cooperativity constant (binding sites)	n	4	$dimensionless$
Reversal potential of AMPA	E_{AMPA}	0	mV
Reversal potential of NMDA	E_{NMDA}	0	mV
Reversal potential of $GABA_A$	E_{GABA_A}	-80	mV
Reversal potential of $GABA_B$	E_{GABA_B}	-95	mV
Voltage-independent forward rate constant for r (α)	AMPA	$1.1\ x\ 10^6$	$M^{-1} * sec^{-1}$
	NMDA	$7.2\ x\ 10^4$	$M^{-1} * sec^{-1}$
	$GABA_A$	$5\ x\ 10^6$	$M^{-1} * sec^{-1}$
Voltage-independent backward rate constant for r (β)	AMPA	190	sec^{-1}
	NMDA	6.6	sec^{-1}

	$GABA_A$	180	sec^{-1}

References:

1. Francis, F., García, M. R. & Middleton, R. H. A single compartment model of pacemaking in dissasociated substantia nigra neurons: stability and energy analysis. *J. Comput. Neurosci.* **35**, 295–316 (2013).

2. Marhl, M., Haberichter, T., Brumen, M. & Heinrich, R. Complex calcium oscillations and the role of mitochondria and cytosolic proteins. *BioSystems* **57**, 75–86 (2000).

3. Cloutier, M. & Wellstead, P. The control systems structures of energy metabolism. *J. R. Soc. Interface* **7**, 651–665 (2010).

4. Cloutier, M. & Wellstead, P. Dynamic modelling of protein and oxidative metabolisms simulates the pathogenesis of Parkinson's disease. *IET Syst. Biol.* **6**, 65–72 (2012).

5. Reed, M. C., Nijhout, H. F. & Best, J. A. Mathematical Insights into the Effects of Levodopa. *Front. Integr. Neurosci.* **6**, 1–24 (2012).

6. Tello-Bravo, D. A Mathematical Model of Dopamine Neurotransmission. *ASU Libraries* **Thesis**, (Arizona State University, 2012).

7. Hong, J.-Y. *et al.* Computational modeling of apoptotic signaling pathways induced by cisplatin. *BMC Syst. Biol.* **6**, 122 (2012).

8. Li, Y., Bertram, R. & Rinzel, J. Modeling N-methyl-D-aspartate-induced bursting in dopamine neurons. *Neuroscience* **71**, 397–410 (1996).

9. Amini, B., Clark, J. W. & Canavier, C. C. Calcium dynamics underlying pacemaker-like and burst firing oscillations in midbrain dopaminergic neurons: a computational study. *J. Neurophysiol.* **82**, 2249–61 (1999).

10. Canavier, C. C. Sodium dynamics underlying burst firing and putative mechanisms for the regulation of the firing pattern in midbrain dopamine neurons: A computational approach. *J. Comput. Neurosci.* **6**, 49–69 (1999).

11. Wilson, C. J. & Callaway, J. C. Coupled oscillator model of the dopaminergic neuron of the substantia nigra. *J. Neurophysiol.* **83**, 3084–3100 (2000).

12. Medvedev, G. S., Wilson, C. J., Callaway, J. C. & Kopell, N. Dendritic synchrony and transient dynamics in a coupled oscillator model of the dopaminergic neuron. *J. Comput. Neurosci.* **15**, 53–69 (2003).

13. Medvedev, G. S. & Kopell, N. Synchronization and Transient Dynamics in the Chains of Electrically Coupled Fitzhugh--Nagumo Oscillators. *SIAM J. Appl. Math.* **61**, 1762–1801 (2001).

14. Komendantov, A. O. & Canavier, C. C. Electrical coupling between model midbrain dopamine neurons: effects on firing pattern and synchrony. *J. Neurophysiol.* **87**, 1526–1541 (2002).

15. Komendantov, A. O., Komendantova, O. G., Johnson, S. W. & Canavier, C. C. A modeling study suggests complementary roles for GABAA and NMDA receptors and

the SK channel in regulating the firing pattern in midbrain dopamine neurons. *J. Neurophysiol.* **91**, 346–357 (2004).

16. Canavier, C. C. & Landry, R. S. An increase in AMPA and a decrease in SK conductance increase burst firing by different mechanisms in a model of a dopamine neuron in vivo. *J. Neurophysiol.* **96**, 2549–2563 (2006).

17. Kuznetsov, A. S., Kopell, N. J. & Wilson, C. J. Transient high-frequency firing in a coupled-oscillator model of the mesencephalic dopaminergic neuron. *J. Neurophysiol.* **95**, 932–947 (2006).

18. Canavier, C. C., Oprisan, S. a, Callaway, J. C., Ji, H. & Shepard, P. D. Computational model predicts a role for ERG current in repolarizing plateau potentials in dopamine neurons: implications for modulation of neuronal activity. *J. Neurophysiol.* **98**, 3006–3022 (2007).

19. Kuznetsova, A. Y., Huertas, M. A., Kuznetsov, A. S., Paladini, C. A. & Canavier, C. C. Regulation of firing frequency in a computational model of a midbrain dopaminergic neuron. *J. Comput. Neurosci.* **28**, 389–403 (2010).

20. Yu, N., Tucker, K. R., Levitan, E. S., Shepard, P. D. & Canavier, C. C. Implications of Cellular Models of Dopamine Neurons for Schizophrenia. in *Progress in molecular biology and translational science* **123**, 53–82 (2014).

21. Drion, G., Massotte, L., Sepulchre, R. & Seutin, V. How Modeling Can Reconcile Apparently Discrepant Experimental Results: The Case of Pacemaking in Dopaminergic Neurons. *PLoS Comput. Biol.* **7**, e1002050 (2011).

22. Oster, A. M. & Gutkin, B. S. A reduced model of DA neuronal dynamics that displays quiescence, tonic firing and bursting. *J. Physiol. Paris* **105**, 53–58 (2011).

23. Ha, J. & Kuznetsov, A. Interaction of NMDA Receptor and Pacemaking Mechanisms in the Midbrain Dopaminergic Neuron. *PLoS One* **8**, e69984 (2013).

24. Zakharov, D., Lapish, C., Gutkin, B. & Kuznetsov, A. Synergy of AMPA and NMDA Receptor Currents in Dopaminergic Neurons: A Modeling Study. *Front. Comput. Neurosci.* **10**, 1–11 (2016).

25. Qian, K., Yu, N., Tucker, K. R., Levitan, E. S. & Canavier, C. C. Mathematical analysis of depolarization block mediated by slow inactivation of fast sodium channels in midbrain dopamine neurons. *J. Neurophysiol.* **112**, 2779–2790 (2014).

26. Yu, N. & Canavier, C. C. A Mathematical Model of a Midbrain Dopamine Neuron Identifies Two Slow Variables Likely Responsible for Bursts Evoked by SK Channel Antagonists and Terminated by Depolarization Block. *J. Math. Neurosci.* **5**, 5 (2015).

27. Cullen, M. & Wong-Lin, K. Integrated dopaminergic neuronal model with reduced intracellular processes and inhibitory autoreceptors. *IET Syst. Biol.* **9**, 245–258 (2015).

28. Muddapu, V. R., Mandali, A., Chakravarthy, V. S. & Ramaswamy, S. A Computational Model of Loss of Dopaminergic Cells in Parkinson's Disease Due to Glutamate-Induced Excitotoxicity. *Front. Neural Circuits* **13**, 11 (2019).

29. Morozova, E. O., Zakharov, D., Gutkin, B. S., Lapish, C. C. & Kuznetsov, A. Dopamine Neurons Change the Type of Excitability in Response to Stimuli. *PLOS Comput. Biol.* **12**, e1005233 (2016).

30. Morozova, E. O. *et al.* Contribution of synchronized GABAergic neurons to dopaminergic neuron firing and bursting. *J. Neurophysiol.* **116**, 1900–1923 (2016).

31. Knowlton, C., Kutterer, S., Roeper, J. & Canavier, C. C. Calcium dynamics control K-ATP channel-mediated bursting in substantia nigra dopamine neurons: a combined experimental and modeling study. *J. Neurophysiol.* **119**, 84–95 (2018).

32. Rumbell, T. & Kozloski, J. Dimensions of control for subthreshold oscillations and spontaneous firing in dopamine neurons. *PLOS Comput. Biol.* **15**, e1007375 (2019).

33. Porenta, G. & Riederer, P. A Mathematical Model of the Dopaminergic Synapse: Stability and Sensitivity Analyses, and Simulation of Parkinson's Disease and Aging Processes. *Cybern. Syst.* **13**, 257–274 (1982).

34. King, R., Barchas, J. D. & Huberman, B. A. Chaotic behavior in dopamine neurodynamics. *Proc. Natl. Acad. Sci.* **81**, 1244–1247 (1984).

35. Justice, J. B., Nicolaysen, L. C. & Michael, A. C. Modeling the dopaminergic nerve terminal. *J. Neurosci. Methods* **22**, 239–252 (1988).

36. Qi, Z., Miller, G. W. & Voit, E. O. A mathematical model of presynaptic dopamine homeostasis: Implications for schizophrenia. *Pharmacopsychiatry* **41**, S89–S98 (2008).

37. Qi, Z., Miller, G. W. & Voit, E. O. Computational systems analysis of dopamine metabolism. *PLoS One* **3**, e2444 (2008).

38. Best, J. A., Nijhout, H. F. & Reed, M. C. Homeostatic mechanisms in dopamine synthesis and release: a mathematical model. *Theor. Biol. Med. Model.* **6**, 21 (2009).

39. Reed, M. C., Best, J. & Nijhout, H. F. Passive and active stabilization of dopamine in the striatum. *Biosci. Hypotheses* **2**, 240–244 (2009).

40. Dreyer, J. K., Herrik, K. F., Berg, R. W. & Hounsgaard, J. D. Influence of phasic and tonic dopamine release on receptor activation. *J. Neurosci.* **30**, 14273–14283 (2010).

41. Dreyer, J. K. & Hounsgaard, J. Mathematical model of dopamine autoreceptors and uptake inhibitors and their influence on tonic and phasic dopamine signaling. *J. Neurophysiol.* **109**, 171–182 (2013).

42. Büchel, F. *et al.* Parkinson's disease: dopaminergic nerve cell model is consistent with experimental finding of increased extracellular transport of α-synuclein. *BMC Neurosci.* **14**, 136 (2013).

43. Destexhe, A., Mainen, Z. F. & Sejnowski, T. J. Kinetic models of synaptic transmission. *Methods Neuronal Model.* 1–25 (1998).
